# A cold shock protein promotes high-temperature microbial growth through binding to diverse RNA species

**DOI:** 10.1038/s41421-021-00246-5

**Published:** 2021-03-16

**Authors:** Zikang Zhou, Hongzhi Tang, Weiwei Wang, Lige Zhang, Fei Su, Yuanting Wu, Linquan Bai, Sicong Li, Yuhui Sun, Fei Tao, Ping Xu

**Affiliations:** 1 State Key Laboratory of Microbial Metabolism, and School of Life Sciences & Biotechnology, Shanghai Jiao Tong University, Shanghai, 200240 People’s Republic of China; 2 Key Laboratory of Combinatorial Biosynthesis and Drug Discovery (Wuhan University), Ministry of Education, and Wuhan University School of Pharmaceutical Sciences, Wuhan, Hubei 430071 People’s Republic of China

**Keywords:** Transcriptional regulatory elements, Cell growth

## Abstract

Endowing mesophilic microorganisms with high-temperature resistance is highly desirable for industrial microbial fermentation. Here, we report a cold-shock protein (CspL) that is an RNA chaperone protein from a lactate producing thermophile strain (*Bacillus coagulans* 2–6), which is able to recombinantly confer strong high-temperature resistance to other microorganisms. Transgenic *cspL* expression massively enhanced high-temperature growth of *Escherichia coli* (a 2.4-fold biomass increase at 45 °C) and eukaryote *Saccharomyces cerevisiae* (a 2.6-fold biomass increase at 36 °C). Importantly, we also found that CspL promotes growth rates at normal temperatures. Mechanistically, bio-layer interferometry characterized CspL’s nucleotide-binding functions in vitro, while in vivo we used RNA-Seq and RIP-Seq to reveal CspL’s global effects on mRNA accumulation and CspL’s direct RNA binding targets, respectively. Thus, beyond establishing how a cold-shock protein chaperone provides high-temperature resistance, our study introduces a strategy that may facilitate industrial thermal fermentation.

## Introduction

It is essential for microbial survival to maintain functional homeostasis despite changes in the external environment. Temperature is one of the most important environmental factors affecting the growth and survival of microbes. Higher temperatures bring various benefits in industrial fermentation, such as accelerating chemical reaction rates, facilitating downstream product recovery, and avoiding risk of microbial contamination. However, higher temperatures are deleterious for many types of cells because of damage to cellular structures such as lipid membranes and disruption of the processing and/or functions of biomolecules such as proteins and RNA^1–4^. Consequently, high temperatures reduce cell viability and fermentation capability, and further reduce production stability and yield.

Thermophilic microorganisms are of scientific and industrial interests due to their unique cellular and metabolic processes enabling them to remain viable and even thrive at high temperatures^5,6^. While there are numerous and diverse mechanisms conferring high temperature tolerance, they are typically grouped into conceptual categories such as membrane metabolism (e.g., increased sterol content)^7^, enzyme properties (e.g., increased numbers of disulfide bonds to improve protein stability)^8^, post-translational processing of protein biosynthesis (e.g., phosphorylation for regulating the activity of enzymes)^9^ and post-transcriptional regulation of RNA metabolism (e.g., molecular chaperones including certain heat shock and cold shock proteins)^10–14^. Several of the high temperature tolerance mechanisms discovered and characterized from thermophiles have been successfully exploited as novel strategies to facilitate the growth of mesophilic organisms (e.g., *E. coli* and *S. cerevisiae*) at elevated temperatures, but progress in this area has been slow^15,16^. Increased tolerance for growth at elevated temperatures offers potential applicable benefits such as decreasing the risk of contamination in non-sterile open fermentation, thereby substantially reducing production costs over traditional sterile fermentation^5,17–20^. Moreover, elevated fermentation temperatures can in some cases cause faster microbial growth and increased overall biomass production^21^, making increased tolerance for high temperature growth an industrially attractive trait.

At high temperatures, heat shock proteins have been repeatedly reported to prevent aggregation and assist in folding of numerous proteins, resulting in a balance of protein homeostasis in general. However, changes at the mRNA level during heat shock response are not well understood. CspA, an intensively studied member of the cold shock protein family in *E. coli*, protects the secondary structures of mRNA, which heavily affects the transcription process of cells following sudden decreases in temperature. The amino acid sequence of CspA shows 43% identity to the eukaryotic Y-box protein family^22^, both of which contain the highly conserved “cold shock domain” (CSD). Several proteins containing CSD show varied nucleic acid-binding activities that affect gene transcription, DNA replication, and DNA repair, while others interact with mRNA to affect translational efficiency^12,13,23,24^. Recently, the cold shock proteins have been highlighted the likely importance in adaptation to stress that were not previously considered to be part of the heat-shock stimulon in *Streptomyces coelicolor*^25^. Thus, we hypothesized that proteins containing CSD from thermophilic bacteria, which already show adaptability to high temperatures, might serve as updated stress-response elements to improve high temperature tolerance of other microbes.

To explore this concept, we first identified and confirmed candidate genes associated with high temperature response and tolerance in a high yield (of l-lactate acid) thermophile strain *Bacillus coagulans* 2-6 (DSM 21869) using integrated multi-omics methods. We next heterologously expressed a gene in *E. coli* encoding the cold shock protein CspL, which increased growth rates and biomass production at both 37 °C and 45 °C; cells expressing *cspL* had a 2.4-fold increase in biomass at 45 °C, and the cell morphology changed. We used bio-layer interferometry assays to demonstrate the nucleotide-binding function of CspL; by using RNA sequencing (RNA-Seq), RNA-immunoprecipitation sequencing (RIP-Seq) and the isobaric Tags for Relative and Absolute Quantification (iTRAQ), characterize the global effects of CspL on mRNA transcript and protein accumulation, and the in vivo RNA targets of this nucleotide-binding protein, respectively. We also showed that a nucleotide-binding-dead variant form of CspL did not increase growth or biomass production at high temperatures. Moreover, growth increases at elevated temperatures were also observed when we expressed *cspL* in *S. cerevisiae* (2.7-fold biomass increase) and *Pseudomonas putida* (1.4-fold increase). The GFP-expression assay showed that the CspL has no deleterious effects on GFP expression or function. The fermentation validations demonstrated significant improvement in the growth and fermentation performance of two industrially relevant microorganisms. In summary, our study revealed that CspL as an RNA chaperone may significantly contribute to global transcriptional and post-transcriptional regulation and the establishment of a new proteostasis.

## Results

### A cold shock protein improves growth of *E. coli*

**Fig. 1:**
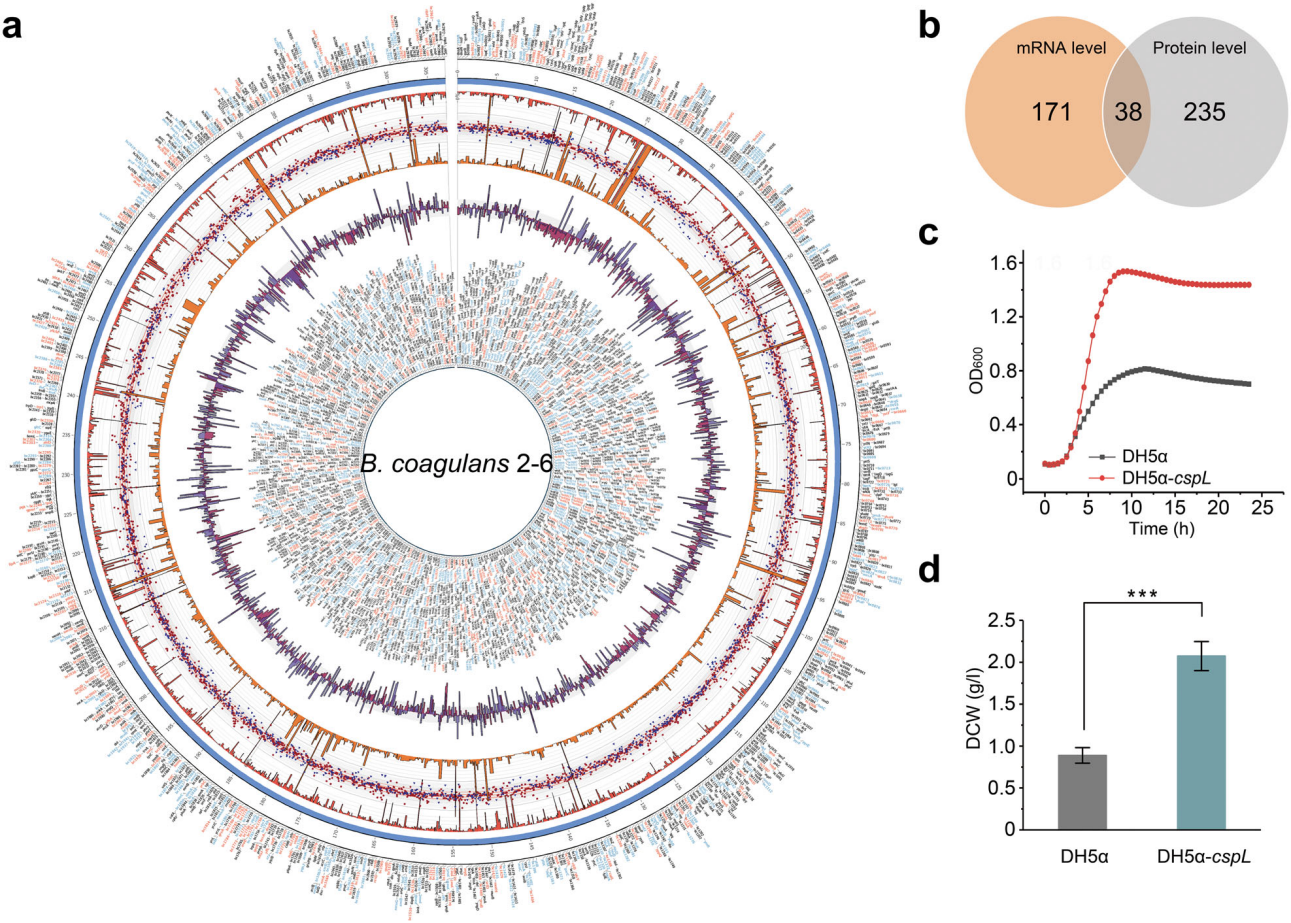
Screening and identifying candidate genes associated with high temperature growth. **a** Global view of *B. coagulans* 2-6 transcriptome and proteome analysis at 37 °C and 60 °C. Concentric circles from the periphery to the core represent the following: (i) differential proteome analysis; the font color of the protein names indicates whether a protein is downregulated (blue), upregulated (red), or shows no significant change (gray) at 60 °C; (ii) chromosomal location; (iii) bar chart in red (inner orientation) representing gene expression levels at 60 °C; (iv) scatter diagram representing protein expression at different conditions (red squares and blue triangles represent 60 °C and 37 °C, respectively); (v) bar chart in orange (outer orientation) representing gene expression levels at the 37 °C condition; (vi) bar chart representing differential expression of the same gene at the mRNA (purple) and protein (blue) levels; (vii) transcriptome analysis; the font color of the gene names indicates whether a gene is downregulated (blue), upregulated (red), or shows no significant changes (gray) at 60 °C. **b** Venn diagram depicting the numbers of genes upregulated at the mRNA and protein levels in *B. coagulans* 2-6. **c** Functional analysis of DH5α-*cspL* at 45 °C. Compared with the control group, DH5α-*cspL* shows a growth advantage. **d** Differences in dry cell weight (DCW) between DH5α-*cspL* and the control at the 45 °C culture condition. ***P < 0.001 (two-tailed Student’s *t*-test).

We previously isolated a *B. coagulans* strain 2-6 (DSM 21869) from a milk processing plant in Beijing by culturing samples from soil at 55 °C^26^. This thermophile can produce optically pure l-lactic acid when cultured at 60 °C, but despite having sequenced its genome, we to date know relatively little about the mechanisms that drive the high-temperature productivity of this strain^27^. We, therefore, observed the growth of *B. coagulans* 2-6 at 37 °C and 60 °C, then used RNA-Seq and iTRAQ proteomics method to investigate how exposure to high temperature affects, respectively, its transcriptome and proteome (Fig. 1a). At the mRNA level, there were 170 differentially accumulated mRNA transcripts (*P* < 0.05, ≥ 2-fold change) between the cells grown at 37 °C and 60 °C (106 mRNAs increased and 64 decreased for the 60 °C samples) (Fig. 1b; Supplementary Tables S1 and S2). Prediction using the Multiple Em for Motif Elicitation (MEME) program indicated that there were no significantly different transcriptional start site structures in the genes encoding the increased vs decreased transcripts (Supplementary Fig. S1a). The iTRAQ experiment identified 275 proteins with differential accumulation (*P* < 0.05, ≥ 2-fold change) between *B. coagulans* 2-6 cells grown at 37 °C or 60 °C (122 proteins increased and 144 decreased for the 60 °C samples; Fig. 1b; Supplementary Tables S3 and S4).

There were 38 transcripts/proteins that were differentially expressed in both the RNA-Seq and iTRAQ datasets (24 increased and 14 decreased in the 60 °C samples; Fig. 1b, Supplementary Table 5). Both KEGG, GO, and protein interaction network analyses suggested enrichment among these candidate thermotolerant gene transcripts/proteins for functional roles related to stress responses and post-transcriptional modification processes (e.g., the global stress regulators RsbV and GsiB and the molecular chaperones Hsp20 and GroEL) (Supplementary Fig. S1b, c). Although extensive efforts were made to delete the 38 candidate genes in *B. coagulans* 2–6, there was no deletion mutants generated. Thus, we subsequently used *E. coli* DH5α cells to heterologously express the 38 candidate thermotolerance-related genes and monitored the growth of the *E. coli* cells at 45 °C (Supplementary Fig. S2).

Expression of several candidate genes resulted in an obvious improvement in the growth of *E. coli* at 45 °C. A cold shock protein L (*BCO26_1317*; hereafter “*cspL*”) was heterologously expressed in *E. coli* DH5α using vector pUC19. Since the detection of CspL by SDS-PAGE was problematic, we confirmed its expression by nano-HPLC-MS/MS analysis (Supplementary Figs. S3 and S4).

**Fig. 2:**
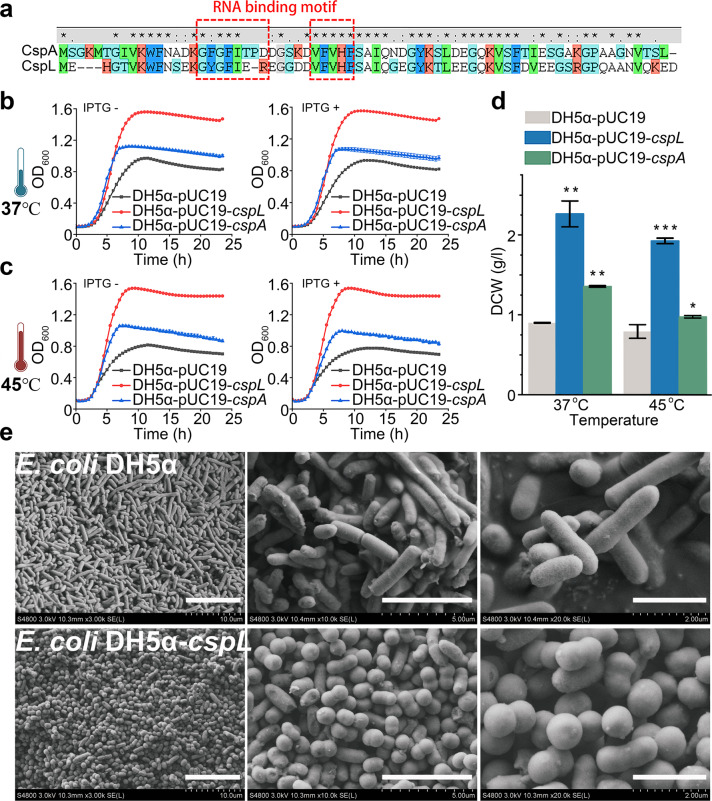
Functional comparison with homologous gene *cspA* in *E. coli*. **a** Amino acid BLAST reveals that *E. coli* CspA and *B. coagulans* 2-6 CspL have 66% identity and share the same RNA-binding motifs. These two binding motifs are highly conserved. **b**, **c**, Growth curves of CspL and CspA expressed in *E. coli* under different treatment conditions, including being induced by IPTG and cultured at 37 °C or 45 °C. **d** DCW of CspL and CspA expressed in *E. coli* at 37 °C and 45 °C. Both DH5α-*cspL* and DH5α-*cspA*’s DCW had significantly improved. **e** Scanning electron microscopy images of the control (upper panel) and CspL (lower panel) strains cultured at 45 °C.

The *cspL* expressed cells exhibited the most pronounced increases in growth (2.5-fold increase) and biomass production (2.4-fold increase in dry cell weight) when grown at 45 °C (Fig. 1c, d). Moreover, three other predictive cold shock genes were found in *B. coagulans* 2-6, listed as *BCO26_1484*, *BCO26_1235* and *BCO26_0628*, respectively. After aligning amino acid sequences, we found that CspL shares 66% identity with its closest *E. coli* homolog CspA (Fig. 2a). We also overexpressed *cspA* in *E. coli* DH5α and this caused significantly increased growth at 45 °C compared to the empty vector control strain; however, the growth increases resulting from overexpression of *cspA* were not as pronounced as those resulting from *cspL* expression. Interestingly, we observed similar increases in growth and biomass production when we grew the strain heterologously expressing *cspL* and *cspA* at 37 °C (Fig. 2b–d) whether induced by IPTG or not. More intriguingly, scanning electron microscopy (SEM) analysis revealed a striking morphological phenotype: for samples grown at both 37 °C and 45 °C, log-phase cells expressing *cspL* were shorter than WT cells and had a ‘peanut-like’ shape (Fig. 2e), but not shown in *cspA*-expressing cells; note that a subsequent RNA-seq analysis showed that expression of *cspL* in *E. coli* resulted in the differential expression of multiple genes associated with cell size/shape, including *ftsZ*, *merC*, and *merD*^28^ (Supplementary Table S6).

### CspL binds RNA in vitro and its RNA-binding activity confers improved growth

As the structure of CspA has been characterized by X-ray diffraction and nuclear magnetic resonance spectroscopy^29,30^, we used the CspA structure (PDB ID: 1MJC) as a template to show that the structure of CspL was also a β-barrel comprising four flexible loops between five β-sheets that act as linkers to form a hollow structure (Supplementary Fig. S5). As CspA is known to bind RNA molecules^22,23^, this structural similarity suggested that CspL may function as an mRNA chaperone to confer the growth- and biomass accumulation-promoting effects that we observed in *E. coli*.

**Fig. 3:**
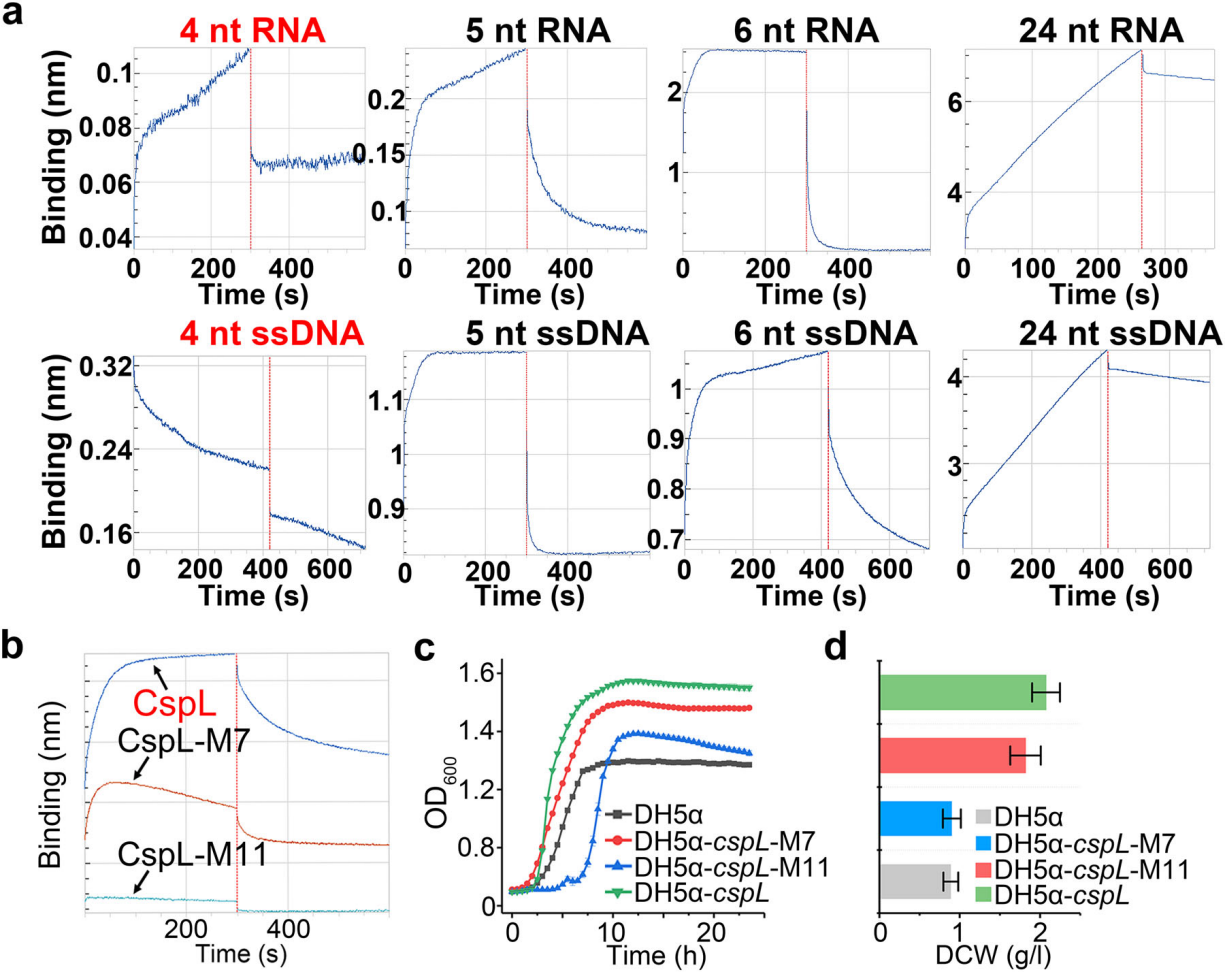
Validation of the function of CspL in vitro. **a** Binding of variously-sized RNA and ssDNA fragments to CspL. The vertical red line separates the association and disassociation steps. An absolute binding value <0.05 nm indicates no binding signal. **b** Bio Layer Interferometer (BLI) for testing seven conserved amino acid-mutations. **c**, **d** The growth curves (**c**) and DCWs (**d**) of mutants compared with the WT and the empty vector control. ** *P* < 0.01 and ***P < 0.001 (two-tailed Student’s *t*-test).

We, therefore, used in vitro bio-layer interferometry (BLI) assays to biochemically assess the potential nucleotide-binding functions of CspL. In this analysis, we tested the interactions of HIS-tag purified CspL (20 μg/ml) with variously sized 5′-biotin-labeled RNA and DNA oligonucleotides that were immobilized to a scaffold using streptavidin. Representative data for the association and dissociation phases of these assays are presented in Fig. 3a and Supplementary Fig. S6. We found that CspL could bind both RNA and single-stranded (ss)DNA, but it could not bind to double-stranded DNA (Supplementary Fig. S6c–f). CspL could bind RNA and ssDNA probes of 24, 6, and 5 nucleotides, but it could not bind RNA and ssDNA probes of only 4 nucleotides (Fig. 3a). As the sequences of the scaffold-bound oligonucleotide targets were randomized, we speculate that CspL may function as a global chaperone for mRNA and/or ssDNA.

Using the conserved domain analysis tools available at NCBI, we identified 11 amino acids located in the putative nucleotide-binding domain of CspL and mutated all of them to alanine. BLI analysis revealed that this variant form of CspL lost its nucleotide-binding capacity (Fig. 3b). Consistent with the hypothesis that the nucleotide-binding function of CspL is responsible for its growth-promoting effects, we observed no significant increases in growth rates or biomass production effects compared with control cells when we expressed this 11 amino acid mutation of CspL in *E. coli* and grew the cells at 45 °C (Fig. 3c, d). We also generated a variant form of CspL with seven residues mutated to alanine, and BLI assays showed that the nucleotide-binding function of this variant was partially impaired (Fig. 3b). Growth assays at 45 °C showed that the growth and biomass production of this seven-mutated-residue variant were intermediate between wild-type CspL and the 11-mutated-residue variant (Fig. 3c, d). We predicted that the structures of mutant proteins with both mutations could not form a stable binding pocket (Supplementary Fig. S7). These findings further support our conclusion that the nucleotide-binding function of CspL confers its observed effects and raises the prospect that it may be possible to tailor the nucleotide-binding functions of CspL to further promote growth at elevated temperatures.

### RIP-seq analysis reveals interaction of CspL and hundreds of mRNA transcripts in vivo

Based on growth, cell morphology, and nucleotide-binding results, we speculate that expressed CspL in *E. coli* may have a global effect at the whole genome level. Having demonstrated the nucleotide-binding functions of CspL and having shown that the observed growth increases were mediated by such binding in vitro, we next used functional genomics profiling methods to further probe the function(s) of CspL in living cells. Global transcriptome profiling with RNA-Seq revealed that *cspL* expression resulted in increased accumulation of transcripts for numerous genes (Supplementary Fig. S8a): at 45 °C, transcripts for 1160 genes (27% of all genes in the *E. coli* genome) were more abundant in *cspL-*expressing cells than in empty-vector control cells. Transcripts for 383 genes were less abundant in the *cspL*-expressing strain.

In the *cspL*-expressing cells, the mRNA accumulation of *cspA* was almost 18-fold more abundant than the empty-vector cells at 45 °C; however, only one-tenth of the empty-vector cell abundance could be detected at 37 °C. This result suggested that CspL could trigger *cspA* transcriptional expression, while *cspA* homologs had the same trends. The interaction network shows that some genes had a close connection with *cspA* (Supplementary Fig. S8b). For example, *rhlE* encodes a type of ATP-dependent RNA helicase^31,32^, suggesting that RNA processing is regulated energetically under the dual stress of temperature and presence of CspL at that exact phase. This is the first time that CspA was found to participate in the high-temperature response. In addition, CspL expression also mediates global regulator gene *fis* and membrane biosynthesis-related genes, such as *lamB*, *malE*, *malM*, and *malK*, were upregulated.

To explore translation level changes, we analyzed 359 proteins identified in the iTRAQ experiment to have differential accumulation (*P* < 0.05, ≥ 2-fold change) between *E. coli* cells with or without expressing CspL grown at 45 °C (69 proteins increased and 294 decreased for the 45 °C samples). The GO analysis suggested that the most changes occurred in biological process and cellular component categories (Supplementary Fig. S8c). It is reasonable to connect these changes with cell morphology changes in *E. coli* DH5α. For example, the proteins DnaK, DnaJ, HtpG, and related DnaK/DnaJ proteins were involved in the high temperature stress response.

**Fig. 4:**
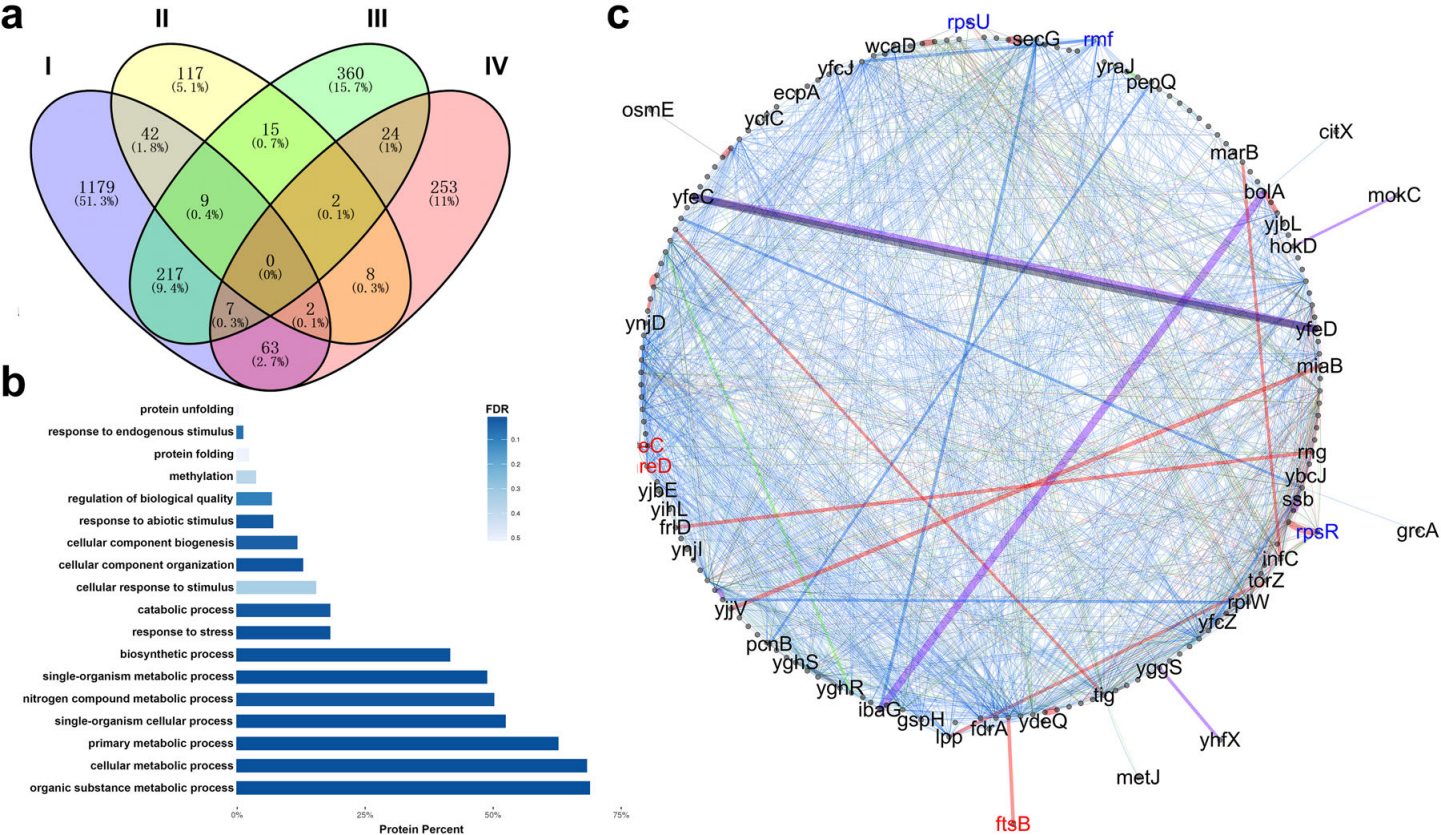
The integration function of CspL in vivo. **a** Venn diagram showing transcriptionally upregulated genes (I), mRNA binding targets of CspL at 37 °C (II) and 45 °C (III), and proteomic level differences (IV). **b** GO Mapper analysis performed on the label-free proteome datasets to reveal the global regulation at 45 °C in *cspL*-expressing cells. **c** The regulation network of binding targets. The gene names in red (*mreC*, *mreD*, and *ftsB*) were directly related to cell wall synthetic processing, gene names in blue (*rpsR*, *rpsU*, and *rmf*) were responsible for ribosome synthetic processing. Branch color indicates the type of interaction. Blue, genetic interactions; purple, shared protein domain; pink, physical interactions; green, co-expression; and gray, other.

To further explore the effect of CspL at the transcriptional level, we next used RIP-seq to identify the RNA binding targets of CspL in *E. coli* cells grown at 37 °C and 45 °C (Supplementary Fig. S8d). Consistent with the idea that CspL is a global chaperone of mRNA, the RIP-seq data demonstrated that, in vivo, CspL binds with the mRNA transcripts of 206 and 662 genes at 37 °C and 45 °C, respectively (Fig. 4a, II&III). The expression of 1605 transcripts (35% of 4583 genes) in *E. coli* were significantly affected by CspL, and the data of BLI binding assay for randomized RNA sequences support the function of CspL as a global chaperone for mRNA transcripts.

**Fig. 5:**
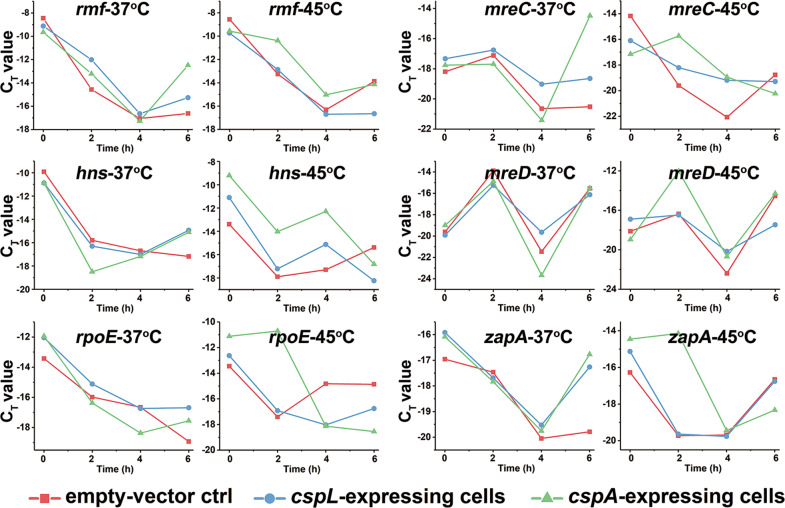
mRNA degradation ratios. Different mRNA degradation ratios show different binding preferences for CspL and CspA.

We were cautious in our interpretation of these data sets with regard to possible trends of enrichment for particular annotation categories among the differentially accumulated transcripts or the CspL targets. We noted significant enrichment for genes related to ribosomes in the RIP-Seq data (Supplementary Fig. S8e). The determination of mRNA level showed that many binding targets of CspL were with varying degrees protected at different temperatures, and *rmf* and *mreC* showed a clear effect at 45 °C (Fig. 5). Apparently, CspL and CpsA reduced mRNA degradation to a certain extent, resulting in a different protective effect.

### CspL improves growth and production capacity of various microorganisms

**Fig. 6:**
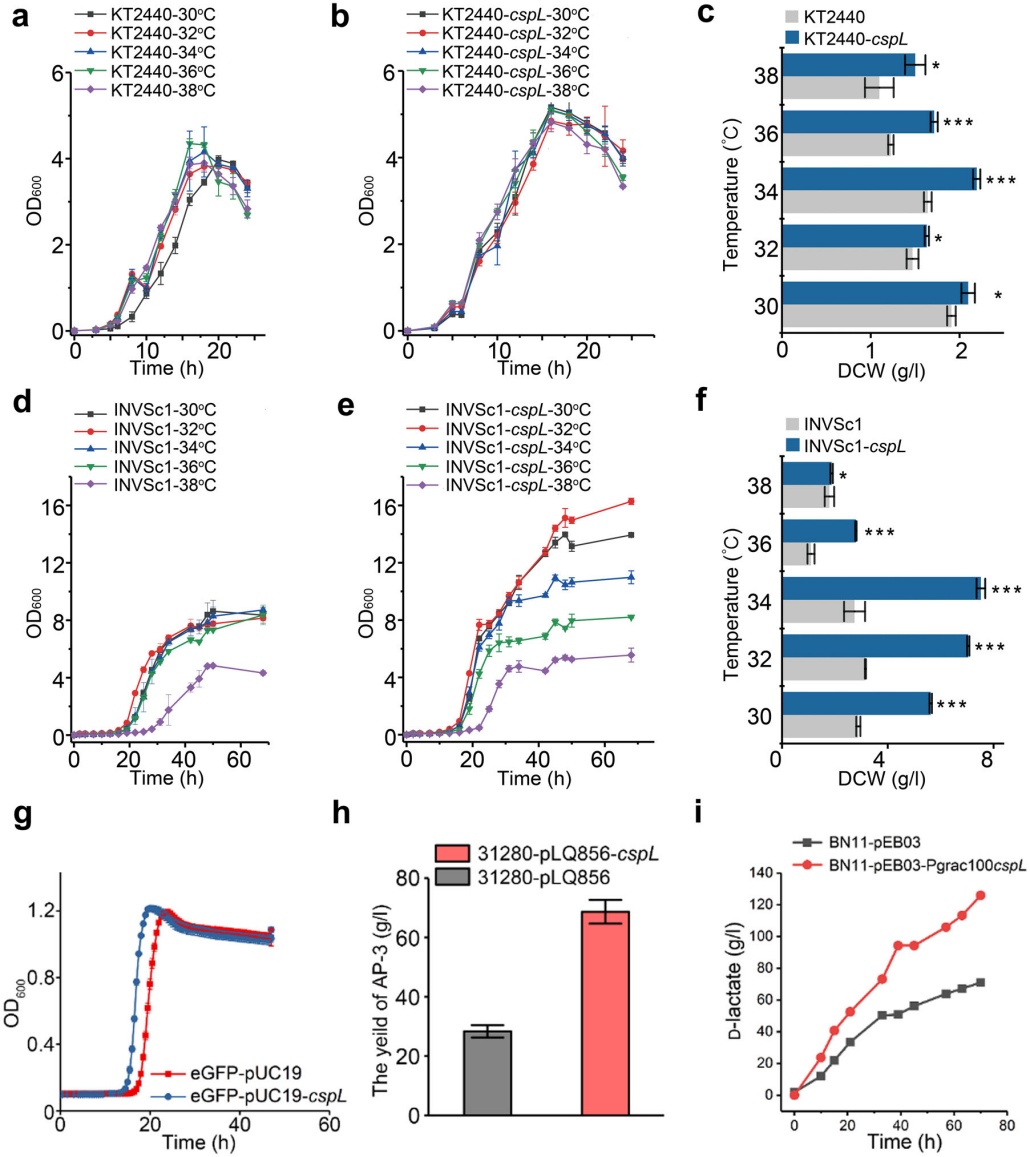
Expression of the *cspL* gene promotes the growth of bacteria and fungi at high temperatures. **a**, **b** Cell growth curves of *P. putida* at different temperatures. **c** DCW of *P. putida* at different temperatures. Statistically significant differences between *P. putida* KT2440 and KT2440-*cspL* were observed at all temperatures tested. *P < 0.05 and ***P < 0.001 (two-tailed Student’s *t*-test). **d**, **e** Cell grow*t*h curves of *S. cerevisiae* at different temperatures. **f** DCW of *S. cerevisiae* at different temperatures. Statistically significant differences between *S. cerevisiae* INVSc1-*cspL* and the control were observed at all temperatures tested. *P < 0.05 and ***P < 0.001 (two-tailed Student’s *t*-test). **g** *cspL* improved growth of the eGPF expressing strain at 45 °C. The strain carrying *cspL* had a shorter lag phase. **h** HPLC analysis indicated that the production of AP-3 was increased by 160% from 29 ± 3 mg/l to 71 ± 6 mg/l in the culture of 31280::pLQ856-*cspL*. **i** Comparison of D-lactate production between *B. licheniformis* BN11-pEB03 (filled square) and BN11-pEB03-Pgrac100*cspL* (filled circle). The growth of BN11-pEB03-Pgrac100*cspL* showed an obvious advantage over BN11-pEB03. The fed-batch time of BN11-pEB03-Pgrac100*cspL* was almost 5 h ahead of the BN11-pEB03.

To explore the possibility that *cspL* may also help increase the high-temperature growth of other microorganisms, we heterologously expressed *cspL* in *S. cerevisiae* INVSc1 and *P. putida* KT2440 and conducted growth assays at elevated temperatures (30 °C to 38 °C in 2 °C increments). In *P. putida* KT2440, both the growth rates and final biomass production of the *cspL-*expressing cells significantly outperformed those of the wild-type cells at all the tested temperatures (Fig. 6a, b). At 36 °C, the *cspL-*expressing cells accumulated 1.4-fold more biomass than the empty-vector control cells (Fig. 6c).

The high-temperature growth-promoting effects of *cspL* were even more obvious in *S. cerevisiae* INVSc1. Not only did the *cspL*-expressing cells significantly outperform the empty-vector control cells at all temperatures tested, there was also an obvious ‘step’ trend in the growth of *cspL*-expressing cells at each increase in temperature (Fig. 6d, e). These results suggest that the growth-promoting effects of CspL in yeast are tightly linked to temperature. The most pronounced increase was observed at 34 °C, with the *cspL*-expressing cells accumulating 2.7-fold more biomass than the empty-vector control cells (Fig. 6f). We did not observe any “peanut-like” phenotypes for *S. cerevisiae* or *P. putida* strains expressing CspL (Supplementary Fig. S9).

We further explored the practical potential of CspL in industrial biotechnology for several applications. First, when enhanced GFP and CspL were simultaneously expressed in *E. coli* at 45 °C, the co-expression of both recombinant proteins significantly increased growth (reduced lag phase) without any deleterious effects on GFP expression or function (Fig. 6g). Second, the expression of CspL in *Actinosynnema pretiosum* ATCC 31280^33^ significantly improved the production of the potent antitumor agent ansamitocin P-3 (a 1.6-fold increase) (Fig. 6h). Third, the expression of CspL in high-temperature (50 °C) cultures of a recently engineered *Bacillus licheniformis* ATCC 14580^34^ strain (BN11) significantly increased both D-lactate production (1.4-fold increase) and glucose consumption (1.33-fold increase) (Fig. 6i). These additional uses of CspL in fermentation applications highlight the strong industrial biotechnological promise of CspL.

## Discussion

Temperature increases can damage cellular homeostasis, interfere with essential functions of cells, and change cellular structures. These phenomena are a concentrated manifestation of changes in protein levels^35^, however, changes at the mRNA levels remained uncharacterized. Over the past decade, several global engineering approaches, including global transcriptional machinery engineering (gTME) and artificial transcription factors^36–38^ have been increasingly explored in reprogramming cellular phenotypes, including tolerances to substrates and products. Most recently, we developed a global regulator engineering (GRE) method based on an exogenous global regulator, IrrE, from *Deinococcus radiodurans* R1^39^. When the *irrE* gene is transferred into hosts, it enhances tolerance to acidic stress. In addition, IrrE endows different hosts with different tolerances, including radiation, osmotic stress, and oxidative stress for *E. coli*^40^; acid stresses for *Zymomonas mobilis*^41^; and salt stress for *Brassica napus*^42^. These results show that a single, global regulatory gene transferred into non-native hosts endows particular tolerances to the recombinant hosts. Practically, it also provides a new insight of synthetic biological strategy at global regulation for improving stress tolerance in prokaryotic and eukaryotic cells.

To comprehensively understand global changes at the mRNA level under high-temperature stress, we first screened and identified several candidate genes associated with high temperature response and tolerance from multi-omic datasets of the excellent industrial strain *B. coagulans* 2-6. Then, we precisely confirmed that a novel cold shock protein, CspL, which acts as an mRNA chaperone to modulate the global gene expressions, affected a signal transduction pathway appropriately in stress conditions. CspL is different from other chaperones at the protein level, reflecting a new function of cold shock protein that could be used to improve the cellular high-temperature tolerance at the global transcriptional level.

Many mechanistic aspects of cold shock proteins are unclear. Based on our findings, the regulatory range of CspL is not limited to responding to temperature stresses, since other pathways, including transcriptional regulation, stress responses, energy metabolism, signal transduction, and protein turnover, were also significantly altered compared to empty-vector cells. More specifically, in our study, the mRNA remaining for *dps* was different in three groups (Supplementary Fig. S10). The *dps* gene encodes for DNA-binding protein from starved cells. Dps becomes the dominant nucleoid-organizing protein in stationary-phase *E. coli* cells and is required for robust survival under stress conditions, including carbon or nitrogen starvation, oxidative stress, metal exposure, and irradiation^43^. In *cspL*-expressing cells, the *dps* gene was captured by CspL in vivo, and the fold-change of enrichment was 1.532 (*p*-value 0.01289651, FDR 0.052) based on the RIP-seq result. At the transcriptional level, we could see that the *dps* transcripts were increased by 1.63- and 2.82-fold in *cspL*-expressing cells compared to empty vector cells at 37 °C and 45 °C, respectively. Under 45 °C conditions, empty vector cells and *cspL*-expressing cells were reduced by 0.19- and 0.38-fold compared to 37 °C. This result shows that *dps* has more transcripts in *cspL*-expressing cells at 45 °C. However, we could not completely confirm that the *dps* transcript accumulation was only affected by the nucleotide binding function of CspL. In this case, we speculate that on the one hand, CspL binds and protects the *dps* mRNA under high temperature conditions; while on the other hand, cells expressing *cspL* might trigger other stress response regulators including Dps in vivo to protect themselves against high ambient temperature stress. From the view of synthetic biology, *cspL* can be considered an evolvable “part” for various stresses. Furthermore, this GRE approach can be extended to exploit other exogenous global regulators from natural or artificial sources for eliciting industrially useful phenotypes.

Future research will likely focus on the implementation and optimization of CspL expression as a tool to promote high-temperature growth, as well as delineating the range of microorganisms that can receive growth-promoting benefits from the transgenic expression of CspL. The large number of targets identified by our RIP-seq analysis makes it clear that CspL functions as a global RNA chaperone that somehow promotes high temperature growth. It will be interesting to determine the extent to which CspL targets particular subsets of transcripts based on some sequence motifs, or other higher-order structural features. Our findings highlight the fact that transgenic *cspL* expression improved *E. coli* growth at its normal 37 °C culture temperature, which underscores the importance of identifying the particular positive contribution of RNA binding by CspL to bacterial growth.

The functions of CspL coincide with the core idea of current synthetic biology. It has long been desired to directly manipulate and modify the core metabolic network or basic functional cellular processing in order to achieve a particular goal more easily for implementing in industrial bacteria. Design from the top level in synthetic biology could result in many advantages compared with normal metabolic engineering. Likewise, when CspL is present, regulation at the transcriptional and post-transcriptional level may alter the natural robustness of metabolic flux distribution in industrial microbes, without increasing the metabolic burden for cells.

## Materials and methods

### Bacterial strains and primers

Detailed information about the bacterial and yeast strains, as well as the plasmids and oligonucleotide primers used in this study is provided in the Supplementary content (Supplementary Tables S7–S9).

### Bacterial growth conditions and quantification of cell density

To evaluate the growth of bacteria under different temperatures, single colonies were grown overnight at 37 °C (or other temperature conditions when required). The detailed information is described in Supplementary Materials and Methods.

### Preparation of proteomes, 2D-LC/MS analysis, and protein identification

The differential proteomics and protein identification were described previously^44^; see Supplementary Materials and Methods for details.

### RNA deep-sequencing and identification of differentially expressed mRNA

Total RNA was extracted from *B. coagulans* 2–6 and *E. coli* DH5α with the RNAiso Plus kit (Takara, Japan). RNA deep-sequencing was performed by a commercial sequencing company (Novogene, China), and data processing were described previously^44^ with slight modifications; see Supplementary Materials and Methods for details.

### Bio-layer interferometry

The interactions between CspL and DNA/RNA fragments were performed by using bio-layer interferometry (BLI). ForteBio Octet RED 96 was used for real‐time analysis of interactions. The measure method was described previously^45,46^ with slight modifications; see Supplementary Materials and Methods for details.

### RIP-seq experiment

RNA-immunoprecipitation sequencing (RIP-seq) was performed to identify RNA targets bound by CspL. The method was described previously^47^ with slight modifications; see Supplementary Materials and Methods for details.

### mRNA level assay

To investigate the ratio of mRNA remaining in *E. coli*, we performed the RT-qPCR to determinate the mRNA level, and the method was modified from previous publication^48^; see Supplementary Materials and Methods for details.

### Heterologously expressing *B. coagulans* 2-6 genes in bacteria and fungi

The gene function validation was performed in *E. coli*, *P. putida*, and *Saccharomyces cerevisiae* under different temperature conditions; see Supplementary Materials and Methods for details.

### Scanning electron microscopy

For scanning electron microscopy, cell cultures were grown in the appropriate medium with antibiotics. Cells were collected by centrifugation and washed three times with 0.1 M PBS buffer (pH 7.4). The cells were then fixed for 1 h at room temperature in 0.1 M PBS buffer containing 2.5% glutaraldehyde. Samples were dehydrated through a graded ethanol series up to absolute ethanol, critical point dried with liquid CO_2_, mounted on aluminum stubs and sputter coated with gold-palladium. Imaging was carried out on a Hitachi S-4800 scanning electron microscope.

### Fermentation validation

To obtain CspL function in industrial microbes, the fermentations were performed in appropriate media with antibiotics. Details are available in Supplementary Materials and Methods.

## Supplementary information

Suppletmentary Information

## Data Availability

The data that support the findings in this study are available within the article, Supplementary information or from the corresponding author upon reasonable request. Source data are provided with this paper.
