# Supplementary material for: A cold shock protein promotes high-temperature microbial growth through binding to diverse RNA species
Source: Cell Discov. 2021 Mar 16;7:15. doi: 10.1038/s41421-021-00246-5 (PMC7966797; doi:10.1038/s41421-021-00246-5)
Supplement: Supplementary file 1 — Suppletmentary Information [file 41421_2021_246_MOESM1_ESM.pdf]

Supporting Information

A cold shock protein promotes high-temperature microbial growth through binding to diverse RNA species

Zikang Zhou[1†], Hongzhi Tang[1†*], Weiwei Wang[1], Lige Zhang[1], Fei Su[1], Yuanting Wu[1], Linquan Bai[1], Sicong Li[2], Yuhui Sun[2], Fei Tao[1], Ping Xu[1*]

[1]*State Key Laboratory of Microbial Metabolism, and School of Life Sciences & Biotechnology, Shanghai Jiao Tong University, Shanghai 200240, People's Republic of China*
[2]*Key Laboratory of Combinatorial Biosynthesis and Drug Discovery (Wuhan University), Ministry of Education, and Wuhan University School of Pharmaceutical Sciences, Wuhan 430071, People's Republic of China*

[†]Z. K. Zhou and H. Z. Tang contributed equally to this work
*Corresponding author: H. Z. Tang or P. Xu

Mailing address: School of Life Sciences & Biotechnology, Shanghai Jiao Tong University, Shanghai 200240, P. R. China

E-mail: tanghongzhi@sjtu.edu.cn or pingxu@sjtu.edu.cn; Tel: +86-21-34204066; Fax: +86-21-34206723

Keywords: heat shock response, global RNA chaperone, CspL, mRNA binding

SI Materials and Methods

Bacterial growth conditions and quantification of cell density

To evaluate the growth of *Bacillus coagulans* 2-6 under different temperatures, cells were grown overnight in 100 ml of GSY medium (20 g/l glucose, 10 g/l yeast extract, 5 g/l tryptone, 5 g/l $CaCO_3$) without antibiotics in 250 ml Erlenmeyer flasks at 37 °C and 60 °C on a rotary shaker (200 rpm). *E. coli* and *Pseudomonas putida* were grown in Luria-Bertani (LB) medium with appropriate antibiotics at 37 °C, and *Saccharomyces cerevisiae* was grown in yeast extract peptone dextrose medium (YEPD medium: 10 g/l yeast extract, 10 g/l peptone, 20 g/l glucose) with appropriate antibiotics at 37 °C. Cell densities were monitored by measuring optical density at 600 nm using a MAPADA V-1200 spectrophotometer. The overnight cultures were used to seed fresh medium (OD_{600} of 0.1 at the time of transfer). Growth at 37 °C and 60 °C was monitored every 2 hours throughout the incubation period. For dry cell weight measurements, a 30 ml cell suspension was centrifuged at 12,000 g for 8 min in pre-weighed microcentrifuge tubes. The cell pellets were washed twice in water and dried at 50 °C until the mass of each tube remained constant over time (typically after ~48 h).

Preparation of proteomes, 2D-LC/MS analysis, and protein identification

Cells of *B. coagulans* 2-6 grown in GSY medium were harvested by centrifugation, washed twice in PBS buffer, and the cell pellets were lysed by resuspending in buffer (8 M urea, 0.05% SDS, 10 mM DTT, 10 mM Tris, pH 8.0) and grinding under liquid nitrogen. After centrifugation at 12,000 g (10 min, 4 °C), the supernatant was mixed with precooled acetone at a volume-to-volume ratio of 1:4. Following overnight incubation at -20 °C, the mixture was centrifuged at 12,000 g (10 min, 4 °C). The pellets were washed with precooled acetone three times, and resuspended in buffer containing 6 M Gu-HCl and 100 mM Tris, pH 8.3. The protein content was measured using a modified Bradford protocol. Total protein (100 μg) was resuspended in buffer containing 10 mM DTT and incubated at 56 °C for 0.5 h. Then 50 mM IAA was added, and the sample was incubated at 25 °C for 40 min. After 3 K ultrafiltration, membrane ultrafiltration, and flushing of the membrane with 100 mM NH_4HCO_3, the pH of the solution was adjusted to 8.0-8.5. Next, 40 μg of sequencing-grade modified trypsin was added to the extract and digestion was carried out overnight at 37 °C with gentle rotation (protein : trypsin ratio = 50 : 1).

In order to separate and analyze the peptides in the samples, we used multi-dimensional liquid chromatography with an Agilent 1100 LC system. Separation in the first dimension

began with the elution of peptides from a strong cation exchange silica column (0.075 mm × 5 cm). Next, a C18 column (0.075 mm × 10 cm) (Column Technology Inc.) was used with a continuous linear salt gradient (0 - 130 min, 2% - 35%; 130 - 135 min, 35% - 90%; 135 - 140 min, 90%; 140 - 141 min, 90% - 2%; 141 - 180 min, 2%); Chromatography conditions: buffer A: H_2O; buffer B: acetonitrile. Finally, a nanospray column was directly interfaced to the orifice of an LTQ Classic ion trap mass spectrometer (ThermoFisher). Nanospray ionization was accomplished with a spray voltage of 3.5 kV and capillary temperature of 200 °C. The m/z scan range was from 400 to 1800.

Database searches for MS and MS/MS spectra were conducted using proteomics discovery software V1.2 (ThermoFisher, CA, USA). Mass spectra were analyzed using Bioworks software. We generated a predicted protein database from the annotated *B. coagulans* 2-6 genome. The peptide matches with an assumed charge state of z = 1 and an XCorr score of > 2.2, or charge state of z = 3 and an XCorr score of > 3.75 were automatically accepted as valid. High scoring peptide matches were automatically identified and retained.

RNA deep-sequencing and identification of differentially expressed mRNA

Total RNA was extracted from *B. coagulans* 2-6 and *E. coli* DH5α with the RNAiso Plus kit (Takara, Japan). RNA deep-sequencing was performed by a commercial sequencing company (Novogene, China). Briefly, sequencing libraries were generated from rRNA-depleted RNA using the NEBNext® Ultra™ Directional RNA Library Prep Kit for Illumina® (NEB, USA) following the manufacturer's recommendations. The libraries were sequenced on the Illumina Hiseq 2000 platform, and 100 bp paired-end reads were generated. Demultiplexed and quality filtered reads were then aligned to *B. coagulans* 2-6 and *E. coli* DH5α reference genome sequence using TopHat (V.2.0.8). The mapped reads from each sample were assembled using Trinity with a reference-based approach.

Cuffdiff (v2.1.1) was used to calculate the RPKM (Reads Per Kilobase per Million mapped reads) of coding genes in each sample. Gene RPKM values were computed by summing the FPKM values of transcripts in each gene group. Cuffdiff provides a statistical method for determining differential expression of digital transcripts or gene expression data using a model based on the negative binomial distribution. Genes with a P value $<$ 0.05, FKPRM $>$ 0.5 and FC $>$ 2 were classified as differentially expressed.

Expressing *B.* coagulans 2-6 genes in *E. coli*, *S. cerevisiae*, and *P. putida*

PCR amplification was carried using the Phanta Super-Fidelity DNA Polymerase (Vazyme, China) according to the manufacturer's instructions. The sequences of all the plasmids produced were verified by restriction mapping and/or DNA sequencing. We cloned 38 genes from *B. coagulans* 2-6 (*Supplementary* Table S5) into the pUC19 vector and then transformed these constructs into *E. coli* DH5α competent cells. Positive transformants were selected on LB medium plates containing ampicillin (50 mg/l) and were confirmed via PCR. We also cloned the *BCO26_cspL* gene into the pYES2 and pME6032 vectors and then transformed these constructs into *S. cerevisiae* and *P. putida* competent cells, respectively. Positive *S. cerevisiae* and *P. putida* transformants were selected on YPD medium containing 50 mg/l ampicillin and LB medium containing 25 mg/l tetracycline, respectively, and were confirmed via PCR.

The growth of the transformants at 37 °C and 45 °C was monitored via absorbance measurements using a Bioscreen C® analyzer (Labsystems, Finland). All transformants were grown overnight at 42 °C and then inoculated into fresh media in Honeycomb plates (10 × 10 wells) containing the appropriate antibiotics. Quintuplicates of each engineered strain were aliquoted into 200 μl wells. The plates were shaken continuously; readings were taken at a wavelength of 600 nm.

Bio-Layer Interferometry (BLI)

Octet RED96 System (ForteBio) was used for BLI studies. Assay was performed in black 96 well plates (NuncF96 MicroWell TM Plates, Thermo Fisher Scientific, Langenselbold, Germany). The total working volume for samples or buffer was 0.2 ml per well. All binding studies were carried out at 25 °C. Prior each assay, streptavidin (SA) biosensor tips (ForteBio) were pre-wetted in 0.2 ml PBS for at least 20 min. In the preparation stage, sequences synthesized by using 5'-biotin modification (sequences are presented in Supplementary Table S10). SA sensors were loaded with biotinylated single strand RNA/DNA (10 μM), in a buffer containing 20 mM PBS, pH 7.4, 100 mM NaCl, 0.1% BSA (w/v) and 0.02% Tween 20 (v/v). After reaching base line in the same buffer, association and dissociation were carried out with purified CspL (20 μg/ml) and buffer respectively. Steady-state binding responses were determined by the overall response (nm) on each sensor sensor. All measurements were performed in triplicates. The association and dissociation responses were baseline corrected processed with the Octet Software (Version 7.0, ForteBio). Interferometry data were globally fit to a simple 1 : 1 Langmuir model calculating the affinities and rate constants (Octet Software, Version 7.0, ForteBio). The association rate constant (k_a) is defined as the rate of

complex formation per second in a 1 molar solution of two reaction partners. The dissociation rate constant (k_d) indicates the stability of this complex. The affinity constant K_D is calculated by the ratio of the k_d/ k_a.

RIP-seq experiment

Cells were cultured overnight in LB medium with ampicillin. After centrifugation the supernatant was discarded, and the pellet was washed twice with 5 ml of ice-cold PBS and lysed with 600 μl of lysis buffer (20 mM Tris-HCl pH 7.4, 150 mM NaCl, 5 mM $MgCl_2$, and 1 mM DTT) containing 1% Triton X-100 and Turbo DNase I (Invitrogen) 25 U/ml. The sample was then clarified by centrifugation for 10 min at 20,000 g, 4 °C. The supernatant was incubated at 4 °C for 30 min with 60 μl of Dynabeads M-270 Streptavidin (Invitrogen) equilibrated with lysis buffer containing 1% Triton X-100. The beads were washed three times with lysis buffer containing 1% Triton X-100 and 1 M NaCl. His-BCO26-CspL and bound RNAs were eluted with 25 μl of lysis buffer containing 5 mM biotin at 4 °C for 30 min. RNAs were extracted with QIAzol (Qiagen) using the Direct-zol RNA miniprep (Zymo Research). Sequencing libraries were prepared using the TruSeq Stranded Total RNA Library Prep Kit with Ribo-Zero Gold (Illumina). Libraries were sequenced on the HiSeq2500 (Illumina) platform.

mRNA level assay

E. coli overnight cultures were diluted 1:100 into 25 ml fresh LB media and incubated at 37 °C or 45 °C until reaching an optical density (OD_{600}) of 0.5. The culture was then placed in a preheated 37 °C shallow water bath to preserve the experiment temperature and 125 μl Rifampicin (100 mg/ml, for a final concentration of 500 μg/ml) were immediately added to the culture to inhibit RNA synthesis. After rifampicin treatment, rifampicin was removed and put the samples back into the incubators. Selected time points were sampled by collecting 1.4 ml from the culture into a pre-chilled tube containing 170 μl of ice-cold stop solution (90% ethanol and 10% saturated phenol) to deactivate cellular processes and RNA-decay. The sample was quickly vortexed and then extracted total RNA by using RNA Easy Fast Kit (TIANGEN, DP451).

Validating the function of CspL in *E. coli* eGFP expressing system, *Actinosynnema pretiosum*, and *Bacillus licheniformis* fermentation

For validating the function of CspL, *E. coli* eGFP expressing system (strain *E. coli* eGFP) was constructed. The gene sequence of eGFP (Pfam: 01353) was synthesized by Sangon (Shanghai) and transferred into *E. coli* DH5α competent cell by harboring plasmid pET28a. Using LB plate and PCR to confirm positive transformants, the plasmid pUC19 harboring *cspL* was then transferred into those competent positive transformants, and confirmed via PCR. The control group used empty pUC19 vector in place of pUC19-*cspL*. Wild type, control, and *E. coli* eGFP-pUC19-*cspL* were grown overnight in LB medium at 45 °C. The overnight culture was inoculated 1 : 100 into fresh LB medium with appropriate antibiotic (20 μg/ml kanamycin and 100 μg/ml ampicillin) to create a culture stock. The 250 ml Erlenmeyer flasks contained 50 ml of culture stock and cultured in the shaker at 45 °C. Every two hours, the cultures were checked for cell density and fluorescence signal. The method of cell density was mentioned previously. An aliquot of the fresh culture stock (200 μl) was transferred into polystyrene 96-well Costar Assay Plate (black with clear flat bottom, Corning Inc., New York). The plate was shaken in linear mode for 30 s and green fluorescence (λ_{ex} = 485 nm, λ_{em} = 535 nm) was monitored using an Infinite F200 multimode reader (TECAN, San Jose, CA).

For evaluating the function of CspL in high value-added industrial microbes, *A. pretiosum* ATCC31280 (used as AP-3 producing strain) and its derivatives were cultured at 30 °C on YMG agar (0.4% yeast extract, 1.0% malt extract, 0.4% glucose, 2.0% agar (w/v), pH 7.2-7.3). For metabolites analysis, the first seed medium (3.0% tryptone soya broth powder, 0.5% yeast extract and 5.0% sucrose (w/v), pH 7.5) was inoculated with agar-grown mycelia and cultivated at 30 °C, 220 rpm for 24h. Subsequently, the second medium (3.0% tryptone soya broth powder, 0.5% yeast extract and 2.5% sucrose, 1.0% soluble starch (w/v), 0.05% isobutanol and 0.05% isopropanol (v/v), pH 7.5) was inoculated with 1 ml of the first seed culture, inoculated for another 24 h at 30 °C. Fermentation medium (yeast extract 0.8%, malt extract 1.0%, sucrose 1.5%, soluble starch 2.5% (w/v), isobutanol 0.5%, isopropanol 1.2% (v/v), pH 7.5) was inoculated with the second seed culture at 10% (v/v) and inoculated at 25 °C and 220 rpm for 7 days. *E. coli* DH10B and *E. coli* ET12567/pUZ8002 were used for plasmid construction and intergeneric conjugation, respectively. For overexpression of the gene *cspL*, the plasmid pLQ856 with the kasOp* promoter cloned into BamHI/SpeI-digested plasmid pDR3 was used. The sequenced *cspL* encoding gene was inserted into plasmid pLQ856 under the control of kasOp* promoter, generating plasmid pLQ856-*cspL*. The recombinant plasmid was introduced into ATCC31280 from *E. coli* ET12567/pUZ8002 through intergeneric conjugation. Additionally, the plasmid pLQ856 was introduced into

ATCC31280 generating a control strain ATCC31280::pLQ856. To quantify AP-3 production, the supernatant of the fermentation broth was extracted with 2 volume of ethyl acetate and evaporated. The residues were dissolved in methanol, passed through 0.22-μm filters, and applied to HPLC. The HPLC analysis was operated on Agilent series 1260 (Agilent Technology, USA) with an Agilent Eclipse Plus-C18 column (4.6 × 150 mm, 5 μm). AP-3 analyzed at a flow rate of 0.5 ml/min, with the following gradient: 0-5 min 10%-50% B, 5-10 min 50%-60% B, 10-20 min 60%-75% B, 20-30 min 75%-95% B, 30-38 min 95% B, 38-39 min 95%-10% B, 39-48 min 10% B (solvent A: water, solvent B: methanol), and detected at 236 nm and 254 nm.

The synthetic DNA fragment with the inducible promoter Pgrac100 and the codon-optimized gene *cspL* was ligated into the *E. coli*-*B. subtilis* shuttle vector pEB03 to generate the plasmid pEB03-Pgrac100*cspL*. The plasmid pEB03-Pgrac100*cspL* and pEB03 were transformed into *B. licheniformis* BN11 using the method as described previously. *B. licheniformis* were statically cultivated in GSY medium at 50 °C for 24 h. The D-lactate fermentation medium is composed of 100 g/l glucose, 40 g/l peanut meal 0.3 g/l neutral protease. The fermentation temperature was controlled at 50 °C and the inoculum volume was 10% (v/v). The pH was maintained at 7.0 by the automated addition of 25% (w/v) $Ca(OH)_2$. The glucose concentration was maintained between 20 g/l and 120 g/l by adding glucose powder. After 10 h of fermentation, 0.1 mM IPTG was added. Glucose concentration was estimated by the SBA-40D biosensor analyzer. Lactate concentration was determined by HPLC using a Bio-Rad Aminex HPX-87H column.

Nano-HPLC-MS/MS analysis for CspL expression

Lysis buffer (1% SDS, 7 M urea, 1x Protease Inhibitor Cocktail (Roche Ltd. Basel, Switzerland)) was added into the samples, which were vibrated and milled for 400 s three times. The samples were then lysed on ice for 30 min and centrifuged at 15,000 rpm for 15min at 4 °C. The supernatant was collected and transferred to a new Eppendorf tube. A total of 100 μg of protein per condition was transferred into a new Eppendorf tube and the final volume was adjusted to 100 μL with 8 M urea. TCEP (2 μL of 0.5 M) was added and the sample was incubated at 37 °C for 1 h, and then 4 μL of 1 M iodoacetamide was added to the sample and the incubated for 40 minutes at room temperature and protected from light. After that, five volumes of -20 °C pre-chilled acetone were added to precipitate the proteins overnight at -20 °C. The precipitates were washed twice using 1 mL pre-chilled 90% acetone aqueous solution and then re-dissolved in 100 μL 100 mM TEAB. Sequence grade modified

trypsin (Promega, Madison, WI) was added at the ratio of 1:50 (enzyme: protein, weight : weight) to digest the proteins at 37 °C overnight. The peptide mixture was desalted by C18 ZipTip and then lyophilized using a SpeedVac. The sample was analyzed by on-line nanospray LC-MS/MS on Orbitrap Fusion™ Lumos™ Tribrid™ mass spectrometer (Thermo Fisher Scientific, MA, USA) coupled to an EASY-nanoLC 1000 system (Thermo Fisher Scientific, MA, USA). A total of 2 μL peptide was loaded (analytical column: Acclaim PepMap C18, 75 μm × 25 cm) and separated with a 60 min gradient. The column flow rate was maintained at 600 nL/min with the column temperature of 40 °C. The electrospray voltage of 2 kV versus the inlet of the mass spectrometer was used. The mass spectrometer was run under data dependent acquisition mode, and automatically switched between MS and MS/MS mode. The parameters were: (1) MS: scan range (m/z) = 350–1500; resolution = 120,000; AGC target = 8e5; maximum injection time = 50 ms; include charge states = 2-7; (2) HCD-MS/MS: resolution = 15,000; isolation window = 4; AGC target = 5e4; maximum injection time = 25 ms; collision energy = 25, 30, 35.

Tandem mass spectra were processed by PEAKS Studio version X+ (Bioinformatics Solutions Inc., Waterloo, Canada). PEAKS DB was set up to search the uniprot_Escherichia_coli&origin database assuming trypsin as the digestion enzyme. PEAKS DB were searched with a fragment ion mass tolerance of 0.02 Da and a parent ion tolerance of 7 ppm. Carbamidomethylation (C) was specified as the fixed modification. Oxidation (M), Deamidation (NQ), QY(X) 314.00, and acetylation (protein N-term) were specified as the variable modifications. The peptides with $-10\lg P \geq 20$ and the proteins with $-10\lg P \geq 20$ and containing at least one unique peptide were filtered.

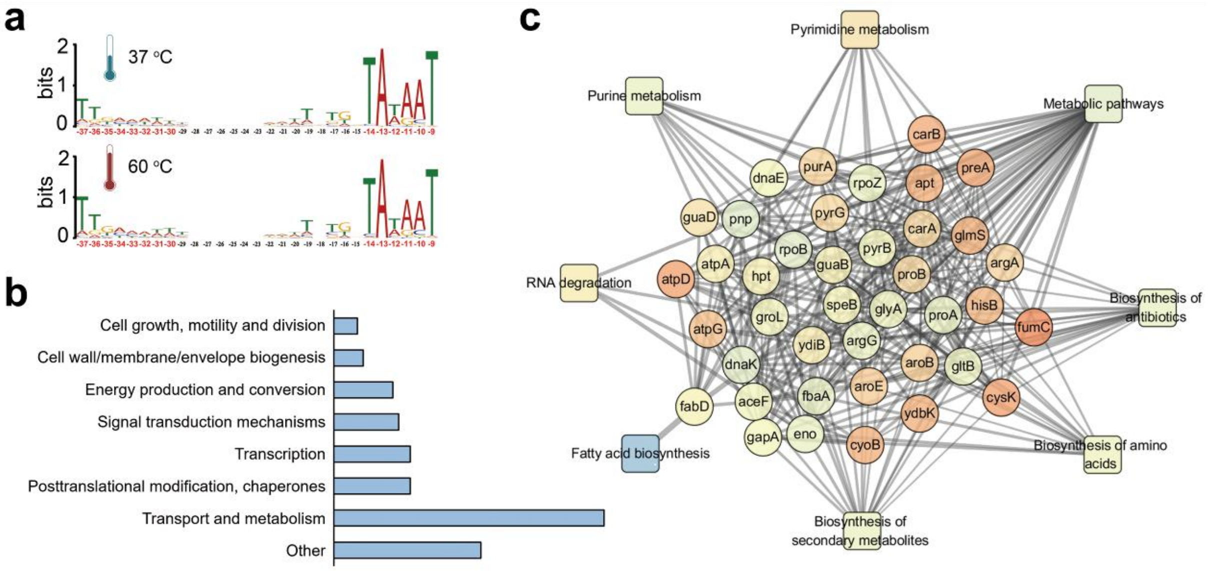

Fig. S1. Transcriptome and proteome analysis of *B. coagulans* 2-6. a Sequence elements of promoters under different conditions. Culturing at 37 °C and 60 °C, the transcriptional start sites, operon structure and the length of 5’ UTR does not show significant difference. The regions -14 to -9 and -37 to -30 are highly conservation under different conditions. **b** GO Slim Mapper analysis was performed on the label-free proteome data set to identify the processes of up-regulated protein expressions at 60 °C. **c** Protein-protein interaction network of *B. coagulans* 2-6. Circle node color indicates genetic neighborhood connectivity. Light blue, low; light yellow, medium; light orange, high. Round rectangle nodes color indicates different classification of GO analysis. Under different conditions of *B. coagulans* 2-6, a subset of highly connected protein nodes involved in key cellular processes undergoes temperature fluctuation.

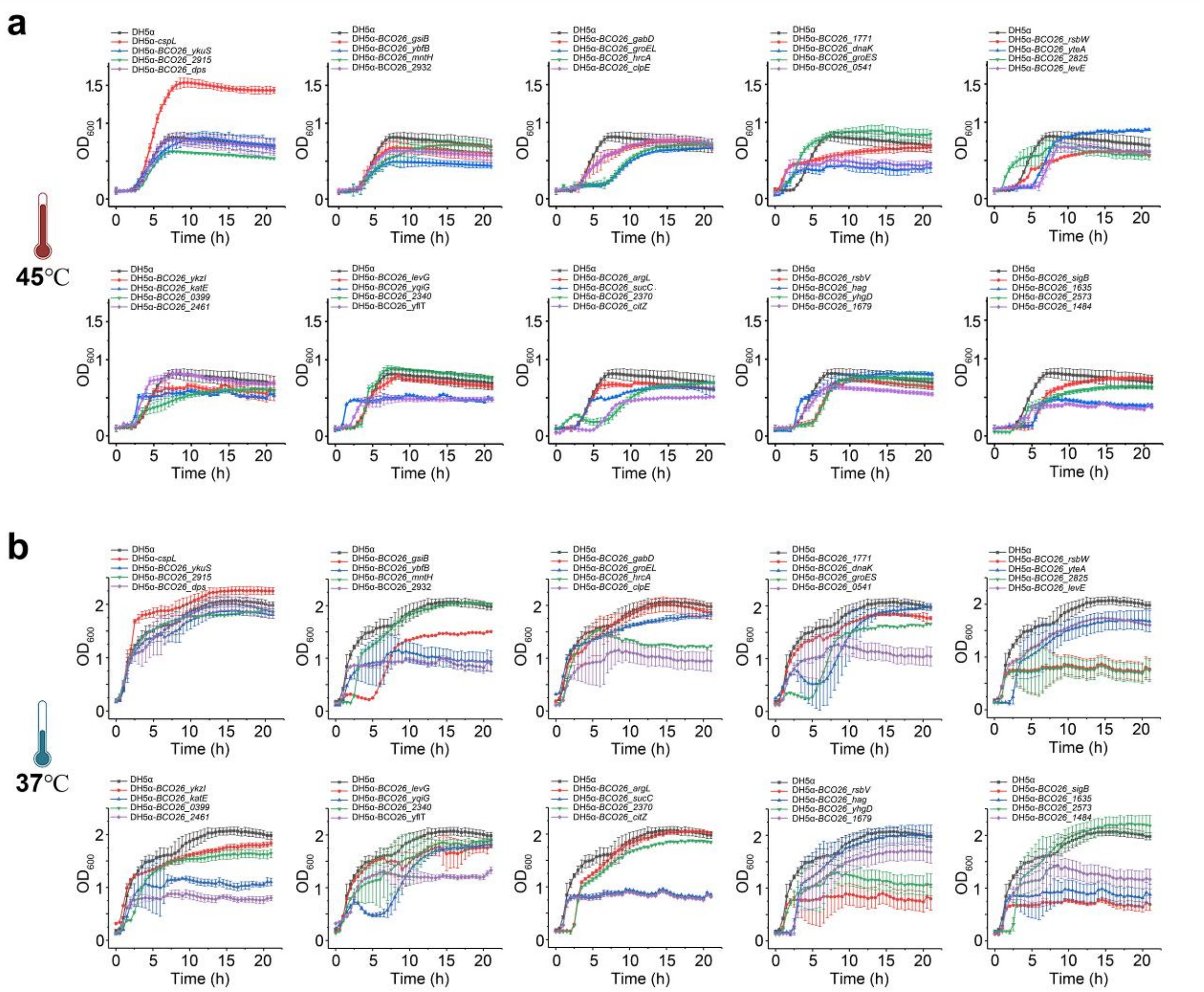

Fig. S2. Growth curves of 38 candidate genes associated with high temperature growth.

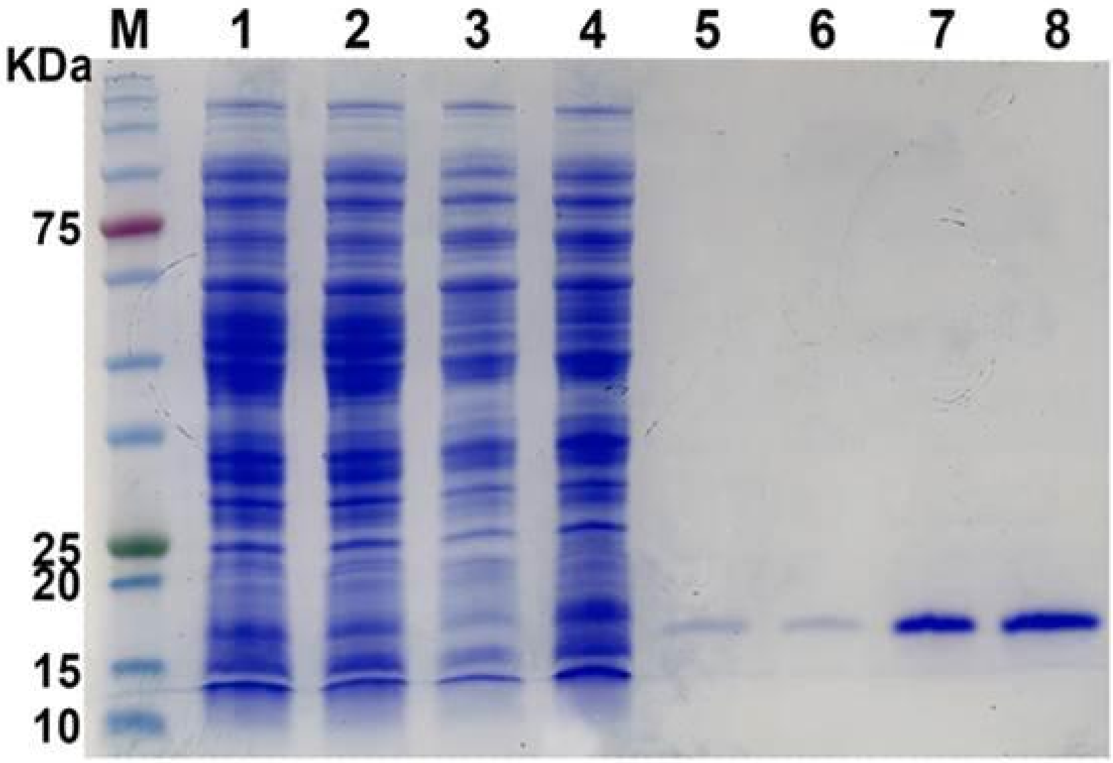

Fig. S3. SDS-PAGE of CspL in *E. coli* DH5α. Lane M, marker proteins; Lanes 1 to 4 represent the whole cell lysate samples and Lanes 5 to 8 represent the purification samples of protein CspL (Ni-NTA affinity, elution by 130 mM imidazole). Lane 1, whole cell lysate, 37 °C, IPTG-; Lane 2, whole cell lysate, 45 °C, IPTG-; Lane 3, whole cell lysate, 37 °C, IPTG+; Lane 4, whole cell lysate, 45 °C, IPTG+; Lane 5, purified protein CspL, 37 °C, IPTG-; Lane 6, purified protein CspL, 45 °C, IPTG-; Lane 7, purified protein CspL, 37 °C, IPTG+; Lane 8, purified protein CspL, 45 °C, IPTG+.

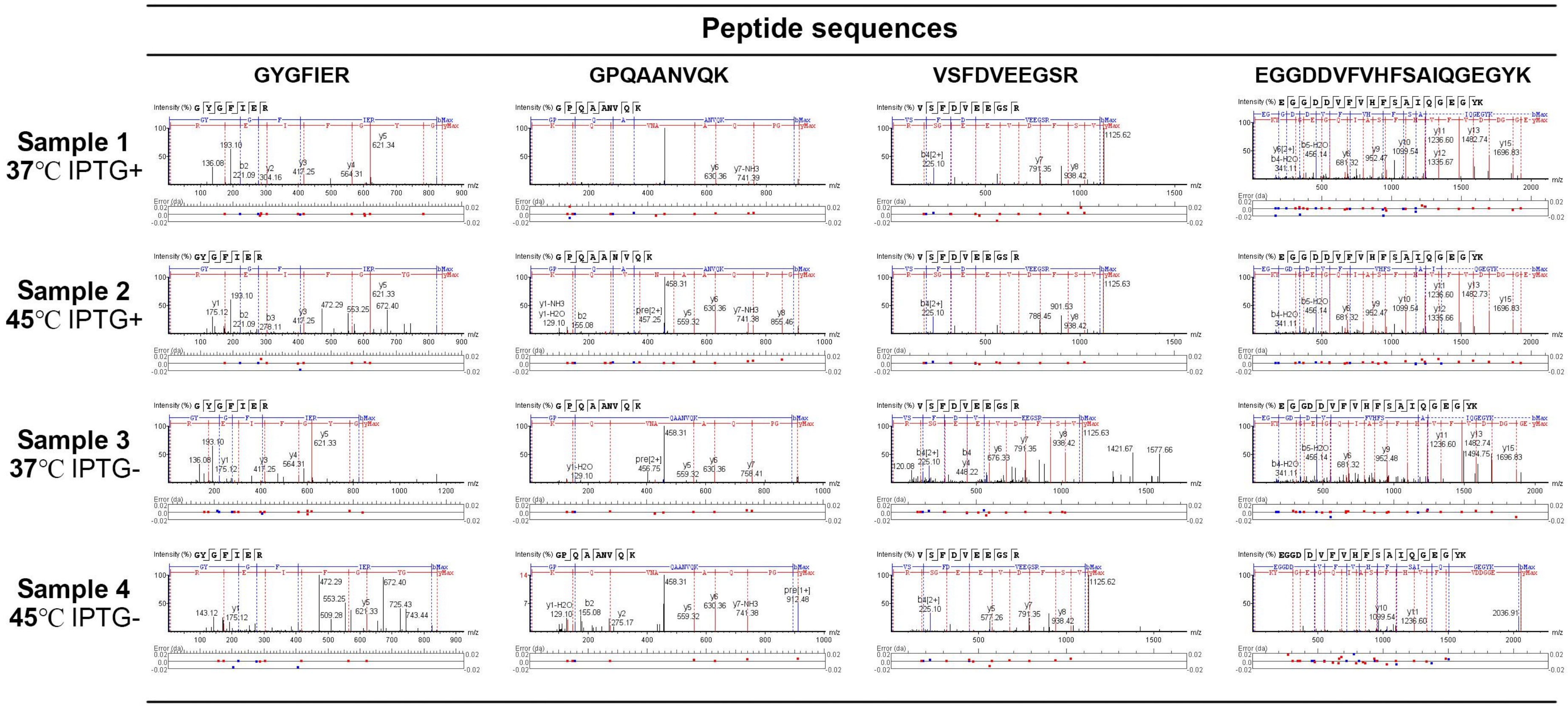

Fig. S4. CspL peptide identification in nano-HPLC-MS/MS. The spectrum showed that four unique peptides of target protein were identified by mass spectrometry under different conditions.

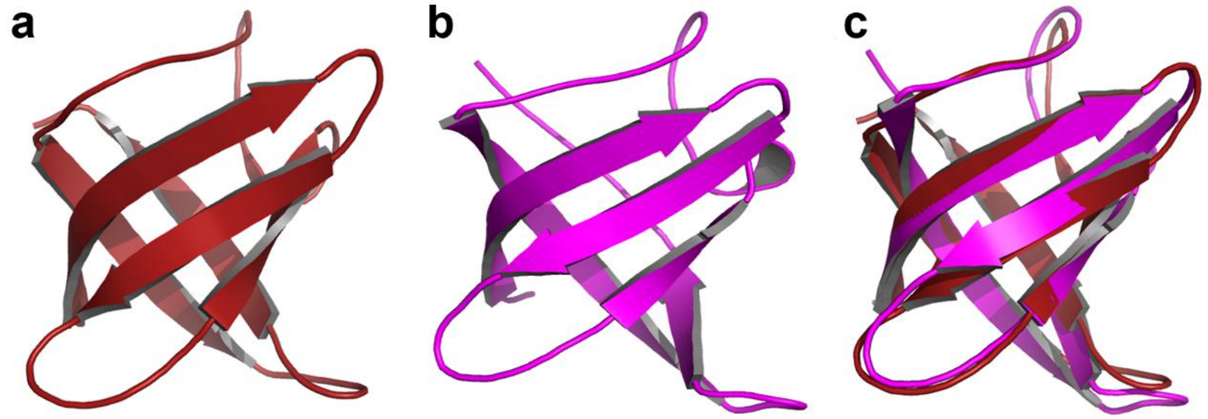

Fig. S5. The predicted structure of CspL. a The predicted structure of CspL. **b** The structure of *E. coli* CspA (PDB: 1mjc.1). **c** Comparison of the predicted CspL structure with the structure reported for CspA, RMSD: 0.97.

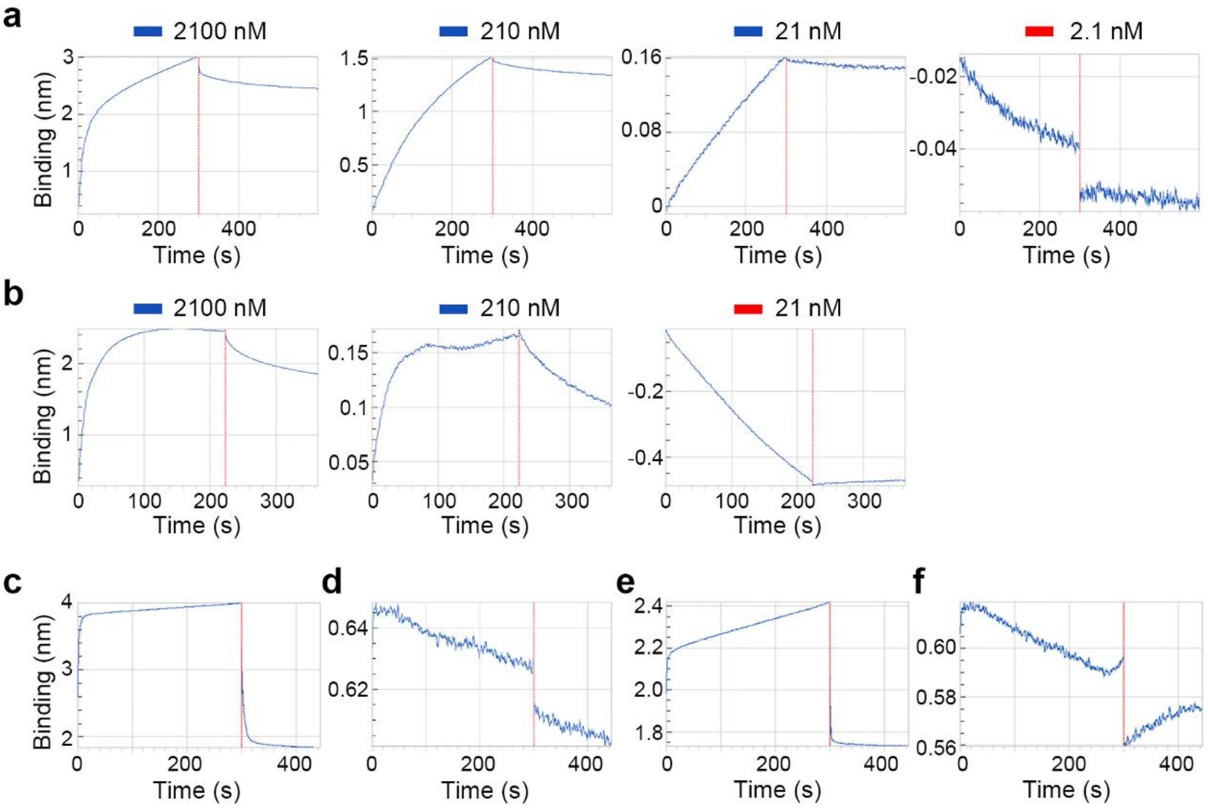

Fig. S6. Concentration limitation of CspL binds to RNA and ssDNA *in vitro*. a Concentration limitation of CspL binds to 18 nt RNA fragment. The concentration gradient sets as 2100 nM, 210 nM, 21 nM, and 2.1 nM. When the concentration of CspL set as 2.1 nM, it lost the binding capacity. **b** Concentration limitation of CspL binds to 18 nt ssDNA fragment. The concentration gradient sets as 2100 nM, 210 nM, and 21 nM. When the concentration of CspL set as 21 nM, it lost the binding capacity. **c** Using ssDNA *gaaC* (biotin-CCGCAGAGAACGACGAGAGC) to bind to CspL, it showed positive binding signal. **d** Using complementary double strand of *gaaC* instead of ssDNA, it showed negative binding signal. **e** Using ssDNA random (biotin-CCGCAGATCCAGACGAGAGC) to bind to CspL, it showed positive binding signal. **f** Using complementary double strand of random gene instead of random ssDNA, it showed negative binding signal.

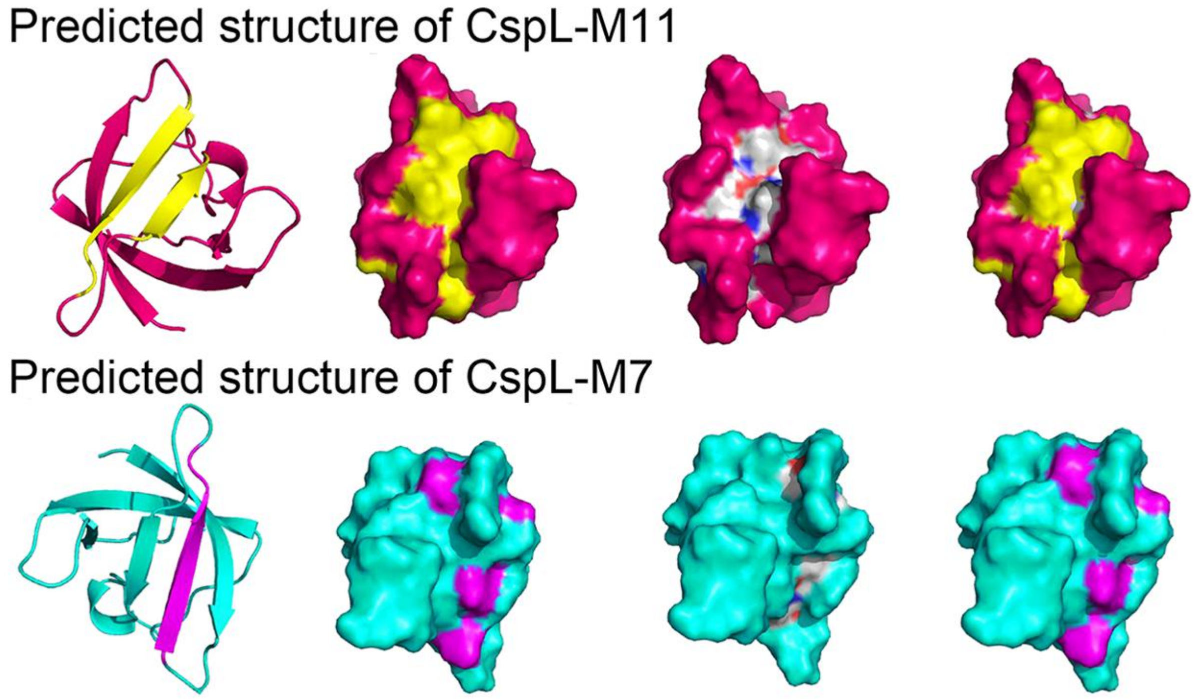

Fig. S7. The mutants of CspL. Two amino acid mutations were synthesized by a commercial company (Sunny, China). Sequences are listed in Supplementary Table S11. The 11 amino acid mutations predicted structure (row in red). The structure of CspL (hot pink) was predicted and a pair of anti-parallel β-sheets considered as putative ligand-binding domains (yellow) were found. When G14, Y15, G16, F17, I18, E19, R20, V26, F27, V28, and H29 in the putative ligand-binding domain were mutated to Ala (CspL-M11), the area of the ligand-binding domain significantly decreased. The ligand-binding domain mutated is colored in gray. The ligand-binding domain of CspL almost completely covered that of CspL-M11, which further demonstrated the shrink in size of the ligand-binding domain after mutation. The G14, Y15, G16, F17, I18, E19, and R20 in the putative ligand-binding domain mutated to Ala (CspL-M7, colored in blue), which slightly decreased the area of the ligand-binding domain. The ligand-binding domain mutated is colored in gray. The ligand-binding domain of CspL completely covered that of CspL-M7, which further demonstrated the shrink in size of ligand-binding domain after mutation.

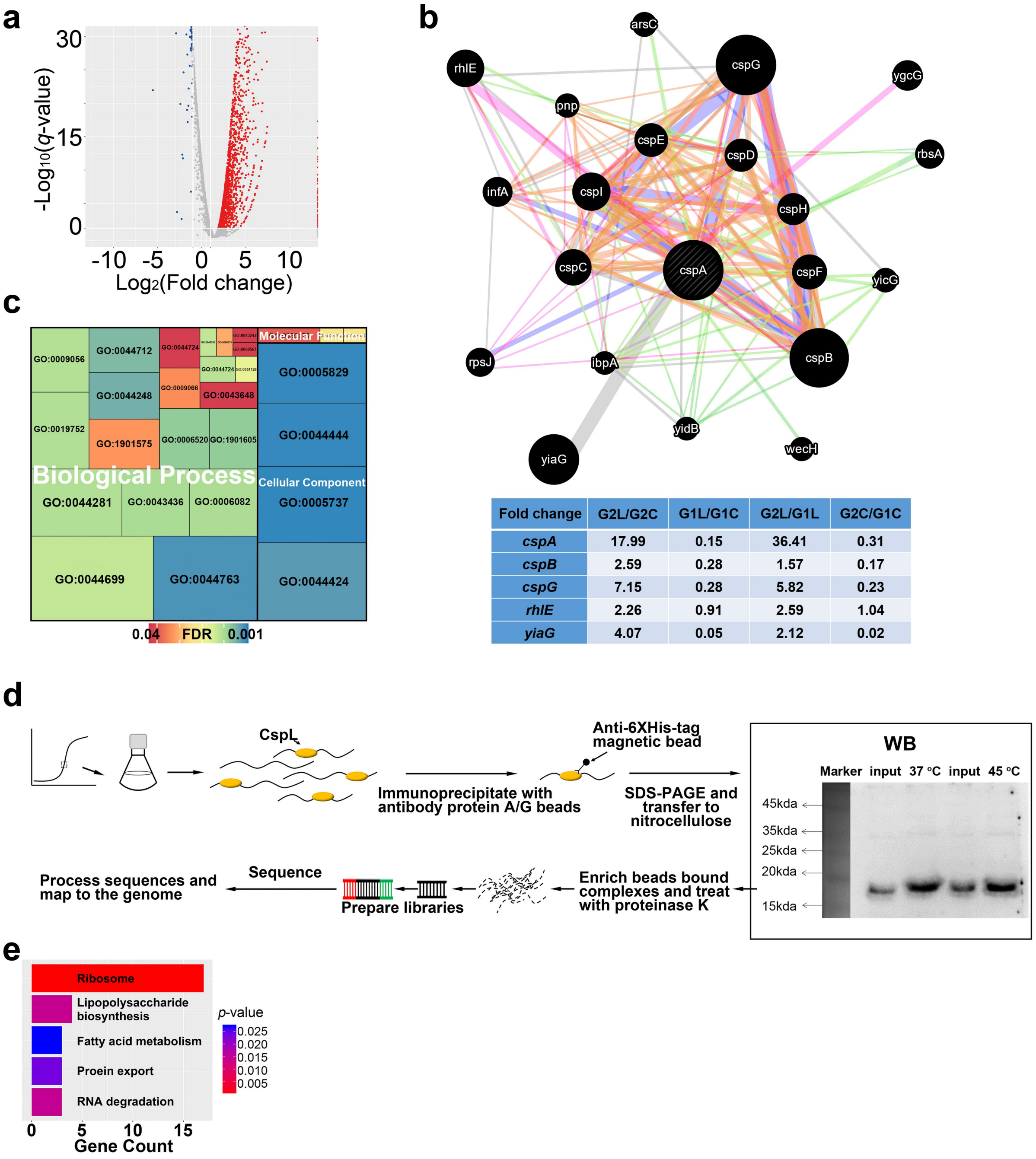

Fold change	G2L/G2C	G1L/G1C	G2L/G1L	G2C/G1C
cspA	17.99	0.15	36.41	0.31
cspB	2.59	0.28	1.57	0.17
cspG	7.15	0.28	5.82	0.23
rhlE	2.26	0.91	2.59	1.04
yiaG	4.07	0.05	2.12	0.02

Fig. S8. The integration function of CspL *in vivo*. a Volcano plot showing differentially expressed genes at the 45 °C condition. The red and blue dots indicate significantly up-regulated and down-regulated genes, respectively, and the gray dots indicate no significant difference. **b** Network of *cspL* gene interaction in *cspL*-expressing *E. coli* cells. The size of nodes represents the importance of the network interaction. Branch color indicates the type of the network. Green, genetic interactions; purple, co-expression; light blue, other; light orange, shared protein domains; and pink, physical interactions. The *cspA* was upregulated by CspL and heat stress. The table shows the closely connected genes' expression level in different cells. G1 and G2 represent 37°C and 45°C culture condition; L and C represent *cspL*-expressing cells and empty-vector cells. **c** GO slim Mapper analysis showing significant different in cellular component. **d** The work flow of RIP-seq. **e** COG analysis of CspL binding targets *in vivo*. The analysis revealed that 17 mRNA targets were related to ribosome category. The color of bars represents various *p*-values.-

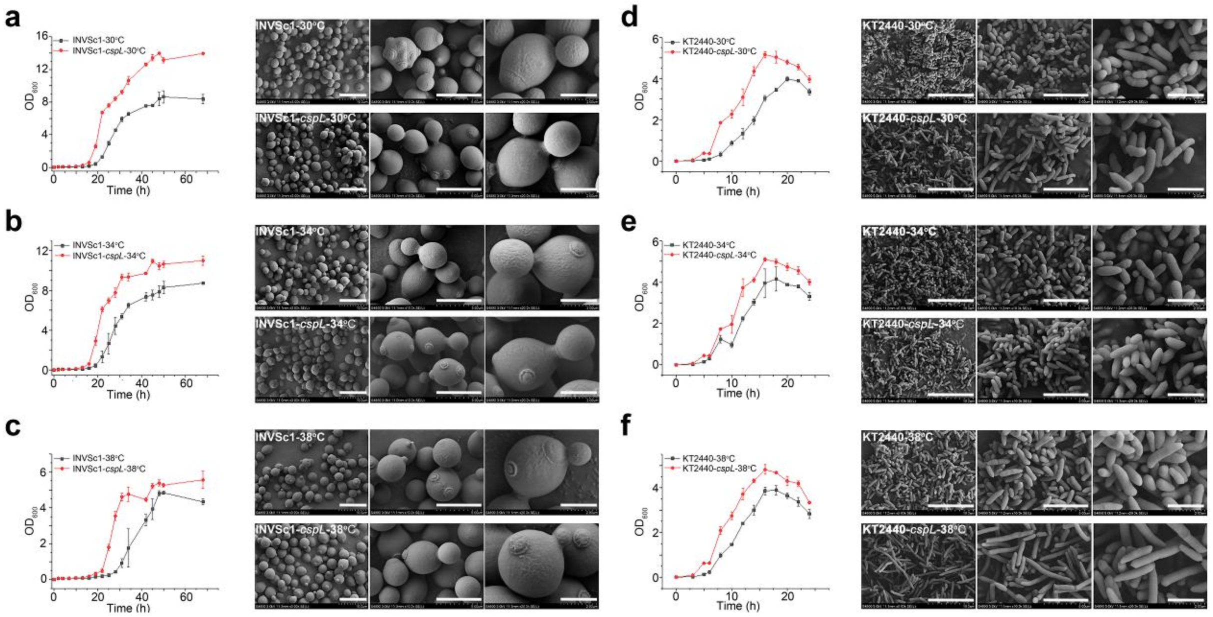

Fig. S9. Growth curves and SEM images of *S. cerevisiae* and *P. putida* at three temperature gradients at 30 °C, 34 °C, and 38 °C. In *S. cerevisiae* INVSc1, **a**, **b**, and **c**, showed CspL expressing grows better than control at different temperature conditions. All three groups have no significant difference in cell morphology, except, the surface texture of CspL expressing strain showed obvious smoother than control. In *P. putida* KT2440, **d**, **e**, and **f**, showed CspL expressing strain grows better than control at different temperature conditions. Two groups of 30 °C and 34 °C have no significant difference. In 38 °C group, the CspL expressing strain seemed slightly longer than control. (scare bar from left to right: 10 µm, 5 µm, and 2 µm)

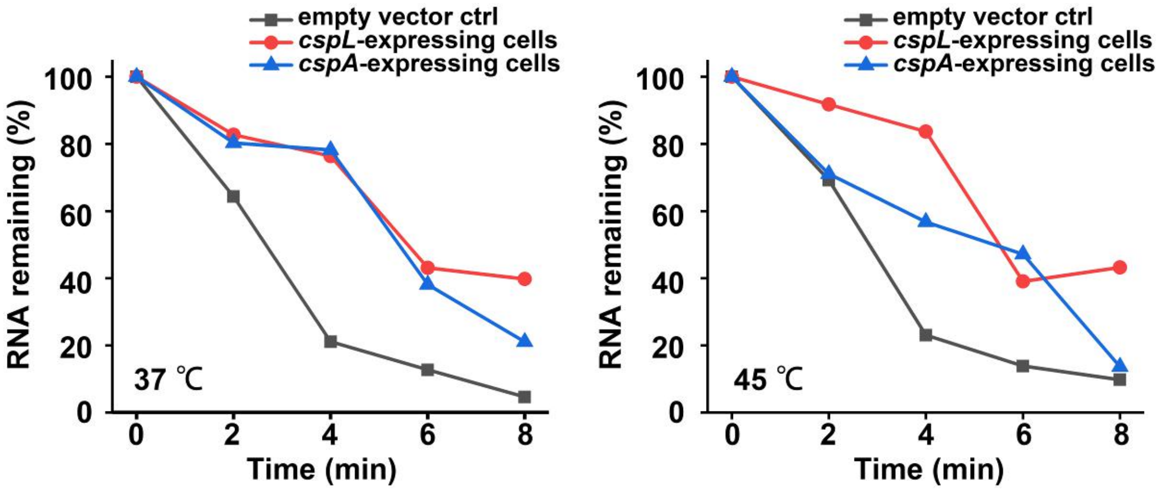

Fig. S10. RNA remaining assays. The RNA remaining assays showed CspL and CspA had the RNA protecting ability compared with empty vector control. Left panel, all samples were cultured at 37 °C; right panel, samples were cultured at 45 °C.

Supplementary Table 1. Up-regulated genes in *B. coagulans* 2-6 by RNA-seq

Locus	Gene	Log_2FC^*	Q-value**	Product
BCO26_0715	*uvrB*	1.3477	0.0488	excinuclease ABC subunit B
BCO26_1892	*clpX*	1.4211	0.0479	ATP-dependent Clp protease, ATP-binding subunit ClpX
BCO26_2971	*trmE*	1.4927	0.0289	tRNA modification GTPase TrmE
BCO26_2177	*yutG*	1.5406	0.0377	phosphatidylglycerophosphatase A
BCO26_1589	*resD*	1.5476	0.0199	winged helix family two component transcriptional regulator
BCO26_0096	*gltX*	1.5499	0.0111	glutamyl-tRNA synthetase
BCO26_0306	*yqiG*	1.6094	0.0296	NADH:flavin oxidoreductase/NADH oxidase
BCO26_2190	*sufS*	1.6196	0.0230	cysteine desulfurase
BCO26_0964	*pdhC*	1.6202	0.0363	hypothetical protein
BCO26_2951	*rplI*	1.6710	0.0290	50S ribosomal protein L9
BCO26_2197	*yusI*	1.7026	0.0200	arsenate reductase and-like protein
BCO26_1748	*hrcA*	1.7120	0.0134	heat-inducible transcription repressor HrcA
BCO26_2975	*rpmH*	1.7207	0.0492	50S ribosomal protein L34
BCO26_0063	*folB*	1.7284	0.0340	dihydroneopterin aldolase
BCO26_1185	*ylxY*	1.7293	0.0117	sporulation protein, polysaccharide deacetylase family
BCO26_1352	*citB*	1.7431	0.0276	aconitate hydratase 1
BCO26_2932	-	1.7449	0.0194	malate/quinone oxidoreductase
BCO26_0951	*ykuF*	1.7638	0.0302	short-chain dehydrogenase/reductase SDR
BCO26_0090	*mcsB*	1.7676	0.0036	ATP:guanido phosphotransferase
BCO26_0833	*mecA*	1.7769	0.0045	Negative regulator of genetic competence
BCO26_1746	*dnaK*	1.7928	0.0036	chaperone protein DnaK
BCO26_0837	*yjbI*	1.7956	0.0116	globin
BCO26_1704	*pbpA*	1.8446	0.0015	penicillin-binding protein transpeptidase
BCO26_2943	*yycJ*	1.8537	0.0272	beta-lactamase domain-containing protein
BCO26_2952	*yybT*	1.8883	0.0019	diguanylate cyclase and phosphoesterase
BCO26_0091	*clpC*	1.8921	0.0019	ATPase AAA-2 domain-containing protein
BCO26_2331	*gltD*	1.9013	0.0189	glutamate synthase, NADH/NADPH, small

				subunit
BCO26_2036	*ytpP*	1.9092	0.0200	Thioredoxin domain-containing protein
BCO26_0601	-	1.9444	0.0340	glycoside hydrolase clan GH-D
BCO26_0973	*suhB*	1.9543	0.0036	inositol monophosphatase
BCO26_0963	*pdhB*	1.9953	0.0049	transketolase central region
BCO26_0714	-	2.0601	0.0007	hypothetical protein
BCO26_1117	*sucC*	2.0623	0.0075	succinyl-CoA synthetase subunit beta
BCO26_2480	*ywaC*	2.0796	0.0004	RelA/SpoT domain-containing protein
BCO26_0753	*yhaR*	2.1031	0.0021	enoyl-CoA hydratase/isomerase
BCO26_2608	*kipA*	2.1125	0.0087	urea amidolyase-like protein
BCO26_1276	*odhB*	2.1401	0.0019	2-oxoglutarate dehydrogenase, E2 subunit, dihydrolipoamide succinyltransferase
BCO26_2370	-	2.1486	0.0070	anti-sigma-factor antagonist
BCO26_2499	*mtbP*	2.1487	0.0436	Modification methylase
BCO26_0089	*mcsA*	2.1513	0.0467	UvrB/UvrC protein
BCO26_1679	-	2.1551	0.0104	ribonucleoside-diphosphate reductase, adenosylcobalamin-dependent
BCO26_2249	-	2.1588	0.0054	hypothetical protein
BCO26_2942	*htrC*	2.2230	0.0073	peptidase S1 and S6 chymotrypsin/Hap
BCO26_1769	*ysfB*	2.2363	0.0246	transcriptional regulator CdaR
BCO26_2562	*bdhA*	2.2874	0.0001	alcohol dehydrogenase zinc-binding domain-containing protein
BCO26_0542	*levF*	2.2885	0.0036	phosphotransferase system PTS sorbose-specific IIC subunit
BCO26_2541	-	2.2965	0.0467	SMC domain-containing protein
BCO26_2741	*sigB*	2.3006	0.0001	Sig B/F/G subfamily RNA polymerase sigma-28 subunit
BCO26_0241	*gabD*	2.3475	0.0002	succinic semialdehyde dehydrogenase
BCO26_0171	*argI*	2.3475	0.0271	arginase
BCO26_1428	*exoAA*	2.3652	0.0105	exodeoxyribonuclease III
BCO26_1353	-	2.3995	0.0194	hypothetical protein
BCO26_2824	*yhcG*	2.4143	0.0351	ABC transporter-like protein
BCO26_1771	-	2.4602	0.0019	heat shock protein Hsp20

BCO26_1419	*rtp*	2.4726	0.0436	replication terminator protein
BCO26_2570	*rbsK*	2.4936	0.0237	ribokinase
BCO26_0895	*yueB*	2.4990	0.0116	hypothetical protein
BCO26_0543	*levG*	2.5090	0.0049	PTS system, mannose/fructose/sorbose family, IID subunit
BCO26_0541	-	2.5409	0.0001	PTS system, mannose/fructose/sorbose family, IIA subunit
BCO26_0590	-	2.6812	0.0481	hypothetical protein
BCO26_0591	-	2.6841	0.0036	NADPH-dependent FMN reductase
BCO26_1635	*yqiW*	2.6885	0.0077	hypothetical protein
BCO26_2944	*yycI*	2.7032	0.0000	YycI protein
BCO26_3000		2.7343	0.0194	putative transposase
BCO26_0474	*yvfI*	2.7422	0.0018	GntR domain-containing protein
BCO26_2742	*rsbW*	2.7626	0.0000	putative anti-sigma regulatory factor, serine/threonine protein kinase
BCO26_2743	*rsbV*	2.8194	0.0000	anti-sigma-factor antagonist
BCO26_2424	*yteA*	2.8282	0.0001	TraR/DksA family transcriptional regulator
BCO26_0740	-	2.8361	0.0000	hypothetical protein
BCO26_2980	-	2.9117	0.0000	hypothetical protein BcoaDRAFT_4102
BCO26_0934	*clpE*	2.9677	0.0234	ATPase AAA-2 domain-containing protein
BCO26_2825	-	2.9723	0.0001	GntR family transcriptional regulator
BCO26_0737	-	2.9815	0.0005	hypothetical protein
BCO26_0739	-	3.0232	0.0000	hypothetical protein
BCO26_0540	*levE*	3.1100	0.0001	PTS system, mannose/fructose/sorbose family, IIB subunit
BCO26_2725	*groES*	3.2119	0.0000	chaperonin Cpn10
BCO26_2375	*ybfB*	3.2466	0.0000	major facilitator superfamily protein
BCO26_2724	*groEL*	3.3122	0.0000	chaperonin GroEL
BCO26_0781	*yhgE*	3.3378	0.0000	YhgE/Pip N-terminal domain-containing protein
BCO26_0400	-	3.3643	0.0000	hypothetical protein
BCO26_2022	*acsA*	3.3911	0.0033	AMP-dependent synthetase and ligase
BCO26_0738	-	3.3949	0.0001	hypothetical protein
BCO26_0780	*yhgD*	3.4007	0.0000	TetR family transcriptional regulator

BCO26_0399	-	3.4057	0.0000	hypothetical protein
BCO26_2638	*gsiB*	3.4856	0.0000	general stress protein
BCO26_1964	*citZ*	3.4935	0.0000	2-methylcitrate synthase/citrate synthase II
BCO26_0880	*ykuS*	3.5367	0.0000	hypothetical protein
BCO26_2535	*katE*	3.5737	0.0132	Catalase
BCO26_1318	-	3.6097	0.0000	hypothetical protein
BCO26_1317	*cspL*	3.6187	0.0000	cold-shock DNA-binding domain-containing protein
BCO26_2573	-	3.6433	0.0000	hypothetical protein
BCO26_2080	*dps*	3.6944	0.0012	Ferritin Dps family protein
BCO26_2371	-	3.7143	0.0000	hypothetical protein
BCO26_0972	*ykzI*	3.8006	0.0000	hypothetical protein
BCO26_1896	-	3.9051	0.0075	hypothetical protein
BCO26_0370	-	3.9154	0.0000	alcohol dehydrogenase GroES domain-containing protein
BCO26_2136	-	3.9586	0.0000	short-chain dehydrogenase/reductase SDR
BCO26_2572	-	4.0593	0.0007	hypothetical protein
BCO26_2079	-	4.0609	0.0004	hypothetical protein
BCO26_2925	-	4.1339	0.0000	hypothetical protein
BCO26_2461	-	4.2752	0.0125	hypothetical protein
BCO26_2926	-	4.3365	0.0000	alanine racemase domain-containing protein
BCO26_2860	*yflT*	4.4587	0.0000	hypothetical protein
BCO26_1328	*yxaB*	4.5939	0.0033	polysaccharide pyruvyl transferase
BCO26_2340	-	4.8911	0.0001	hypothetical protein
BCO26_1065	-	-	0.0488	transposase IS4 family protein

*Log_2FC: the logarithm of fold change, **Q-value: adjustment of *P*-value.

Supplementary Table 2. Down-regulated genes in *B. coagulans* 2-6 by RNA-seq

Locus	Gene	log_2FC*	Q-value**	Product
BCO26_1863	*comC*	-9.4071	0.0036	Prepilin peptidase
BCO26_0208	*gntP*	-8.2333	0.0196	gluconate transporter
BCO26_1379	*yxlA*	-6.6937	0.0202	cytosine/purines uracil thiamine allantoin permease
BCO26_1506	*pbuX*	-6.0585	0.0012	xanthine permease
BCO26_2004	*ycgN*	-5.3972	0.0000	delta-1-pyrroline-5-carboxylate dehydrogenase
BCO26_0804	*yxaH*	-4.8155	0.0144	hypothetical protein
BCO26_2663	*purS*	-4.7138	0.0000	phosphoribosylformylglycinamidine synthase, purS
BCO26_2429	*yuaF*	-4.4123	0.0015	hypothetical protein
BCO26_0140	-	-4.3427	0.0049	hypothetical protein
BCO26_2003	*ycgM*	-4.2934	0.0000	Proline dehydrogenase
BCO26_0828	-	-4.2739	0.0000	binding-protein-dependent transport systems inner membrane component
BCO26_2660	*purF*	-4.1184	0.0013	amidophosphoribosyltransferase
BCO26_2659	*purM*	-4.1062	0.0006	phosphoribosylformylglycinamidine cyclo-ligase
BCO26_1866	-	-3.9611	0.0208	Tfp pilus assembly protein ATPase PilM-like protein
BCO26_0639	*lytE*	-3.9496	0.0064	NLP/P60 protein
BCO26_2658	*purN*	-3.8162	0.0010	phosphoribosylglycinamide formyltransferase
BCO26_0070	-	-3.7475	0.0228	hypothetical protein
BCO26_2664	*purC*	-3.7138	0.0000	phosphoribosylaminoimidazole-succinocarboxamide synthase
BCO26_2661	*purL*	-3.6640	0.0000	phosphoribosylformylglycinamidine synthase II
BCO26_2428	*yuaG*	-3.5600	0.0026	hypothetical protein
BCO26_1865	-	-3.5231	0.0208	Fimbrial assembly family protein
BCO26_0395	-	-3.5059	0.0077	cell envelope-related transcriptional attenuator
BCO26_0982	*ctaB*	-3.3928	0.0004	protoheme IX farnesyltransferase
BCO26_1441	*yhcI*	-3.3455	0.0363	hypothetical protein

BCO26_1869	-	-3.2651	0.0285	twitching motility protein
BCO26_1530	*panB*	-3.1314	0.0000	3-methyl-2-oxobutanoate hydroxymethyltransferase
BCO26_1198	*yufO*	-2.9613	0.0000	ABC transporter-like protein
BCO26_0872	*natB*	-2.9289	0.0031	ABC-2 type transporter
BCO26_2467	*fruR*	-2.8028	0.0234	DeoR family transcriptional regulator
BCO26_2268	*pit*	-2.7363	0.0005	phosphate transporter
BCO26_1235	*cspD*	-2.7183	0.0116	cold-shock DNA-binding domain-containing protein
BCO26_0254	*czcD*	-2.6722	0.0069	cation diffusion facilitator family transporter
BCO26_1529	*panC*	-2.6180	0.0005	pantoate/beta-alanine ligase
BCO26_0376	*lytD*	-2.5334	0.0152	Mannosyl-glycoprotein endo-beta-N-acetylglucosaminidase
BCO26_0312	*yubB*	-2.5161	0.0009	undecaprenol kinase
BCO26_2931	-	-2.5026	0.0214	polar amino acid ABC transporter inner membrane subunit
BCO26_2798	*atpF*	-2.4974	0.0009	ATP synthase F0 subunit B
BCO26_0795	-	-2.4424	0.0010	hypothetical protein
BCO26_1871	-	-2.3884	0.0024	hypothetical protein
BCO26_0871	*natA*	-2.3256	0.0199	ABC transporter-like protein
BCO26_0126	*rplP*	-2.3083	0.0250	50S ribosomal protein L16
BCO26_1980	*ald*	-2.2652	0.0125	alanine dehydrogenase
BCO26_1197	*yufN*	-2.2543	0.0003	basic membrane lipoprotein
BCO26_2802	*glyA*	-2.2229	0.0105	glycine hydroxymethyltransferase
BCO26_0134	*rplR*	-2.1865	0.0018	50S ribosomal protein L18
BCO26_2796	*atpA*	-2.1209	0.0114	ATP synthase F1 subunit alpha
BCO26_1438	*mntH*	-2.0894	0.0056	Mn^{2+}/Fe^{2+} transporter, NRAMP family
BCO26_0981	*ctaA*	-2.0466	0.0481	cytochrome oxidase assembly
BCO26_0640	-	-2.0442	0.0101	methyl-accepting chemotaxis sensory transducer
BCO26_0142	*rpsM*	-2.0159	0.0302	30S ribosomal protein S13
BCO26_0125	*rpsC*	-2.0037	0.0245	30S ribosomal protein S3
BCO26_0443	-	-1.9891	0.0116	glycerol dehydrogenase

BCO26_0827	-	-1.9859	0.0053	family 5 extracellular solute-binding protein
BCO26_1125	*hslU*	-1.9747	0.0015	heat shock protein HslVU, ATPase subunit HslU
BCO26_0132	*rpsH*	-1.9086	0.0228	30S ribosomal protein S8
BCO26_0135	*rpsE*	-1.8813	0.0083	30S ribosomal protein S5
BCO26_0530	-	-1.8492	0.0488	hypothetical protein
BCO26_2267	*ykaA*	-1.8447	0.0281	hypothetical protein
BCO26_1126	*codY*	-1.8339	0.0467	GTP-sensing pleiotropic transcriptional repressor CodY
BCO26_0531	*ldh*	-1.7642	0.0254	L-lactate dehydrogenase
BCO26_1253	-	-1.7337	0.0467	methyl-accepting chemotaxis sensory transducer
BCO26_2665	*purB*	-1.7075	0.0481	adenylosuccinate lyase
BCO26_0660	*hag*	-1.6965	0.0374	flagellin
BCO26_0670	-	-1.6779	0.0458	Gamma-glutamyltransferase

*Log_2FC: the logarithm of fold change, **Q-value: adjustment of *P*-value.

Supplementary Table 3. Up-regulated proteins in *B. coagulans* 2-6 by iTRAQ

Locus	Gene	log_2FC*	Q-value**	Description
BCO26_0136	*rpmD*	7.0227	0.0220	50S ribosomal protein L30
BCO26_2925	-	5.9648	0.0001	Uncharacterized protein
BCO26_0880	-	5.8541	0.0001	UPF0180 protein BCO26_0880
BCO26_1317	*cspL*	5.6614	0.0000	Cold-shock DNA-binding domain protein
BCO26_0255	*glmS*	5.5967	0.3156	Glutamine--fructose-6-phosphate aminotransferase
BCO26_2102	*copZ*	5.5448	0.0001	Copper ion binding protein
BCO26_2080	-	5.5186	0.0000	Ferritin Dps family protein
BCO26_0548	*ysnF*	5.4055	0.0000	Uncharacterized protein
BCO26_0504	-	5.2885	0.0000	Uncharacterized protein
BCO26_1740	*yflL*	5.0693	0.0000	Acylphosphatase (EC 3.6.1.7)
BCO26_0043	-	4.8450	0.0001	Transcriptional regulator, AbrB family
BCO26_0141	*infA*	4.7946	0.0000	Translation initiation factor IF-1
BCO26_0425	-	4.7523	0.0000	Glycine betaine/L-proline ABC transporter, ATPase subunit
BCO26_1491	*yneJ*	4.5668	0.0140	Uncharacterized protein
BCO26_2638	*gsiB*	4.4977	0.0184	General stress protein
BCO26_0441	-	4.4727	0.0025	Uncharacterized protein
BCO26_0164	-	4.4092	0.0000	Cytochrome bd ubiquinol oxidase subunit I
BCO26_2375	-	4.2469	0.0000	Major facilitator superfamily MFS_1
BCO26_0525	-	4.2197	0.0589	Transcriptional regulator
BCO26_1438	*mntH*	4.1401	0.0000	Divalent metal cation transporter MntH
BCO26_1778	-	4.0029	0.0000	Heat shock protein Hsp20
BCO26_1458	*proB*	3.9946	0.0007	Glutamate 5-kinase (EC 2.7.2.11) (Gamma-glutamyl kinase) (GK)
BCO26_0167	-	3.7879	0.0002	ABC transporter, CydDC cysteine exporter (CydDC-E) family, permease/ATP-binding protein CydC
BCO26_0423	-	3.7786	0.0005	Substrate-binding region of ABC-type glycine betaine transport system
BCO26_0505	-	3.7451	0.0002	Quinone oxidoreductase, YhdH/YhfP family
BCO26_0921	*mrgA*	3.5279	0.0005	Ferritin Dps family protein
BCO26_1788	-	3.5157	0.0000	Alkyl hydroperoxide reductase, F subunit
BCO26_2932	*mqo*	3.5035	0.0000	Probable malate:quinone oxidoreductase
BCO26_0241	-	3.5013	0.0000	Aldehyde dehydrogenase
BCO26_0939	*ptsH*	3.4935	0.0000	Phosphotransferase system, phosphocarrier protein HPr
BCO26_1725	*rpoD*	3.4662	0.0004	RNA polymerase sigma factor SigA
BCO26_2724	*groEL*	3.3843	0.0000	60 kDa chaperonin (GroEL protein) (Protein Cpn60)
BCO26_0166	*cydC*	3.2730	0.0000	ABC transporter, CydDC cysteine exporter (CydDC-E) family, permease/ATP-binding protein CydD
BCO26_0424	-	3.2591	0.0002	Binding-protein-dependent transport systems inner membrane component

BCO26_0610	-	3.2583	0.0005	Uncharacterized protein
BCO26_0300	-	3.1971	0.0001	Glycerol-3-phosphate dehydrogenase (EC 1.1.5.3)
BCO26_0207	*treR*	3.1921	0.0007	Transcriptional regulator, GntR family
BCO26_0092	*radA*	3.1826	0.0045	DNA repair protein RadA
BCO26_2962	-	3.0935	0.0029	Uncharacterized protein
BCO26_1721	-	3.0791	0.0011	Uncharacterized protein
BCO26_1748	*hrcA*	3.0552	0.0007	Heat-inducible transcription repressor HrcA
BCO26_1648	-	3.0521	0.0000	DNA repair protein RecN (Recombination protein N)
BCO26_0240	-	3.0461	0.0001	Anti-sigma-factor antagonist
BCO26_2617	*htpG*	3.0134	0.0001	Chaperone protein HtpG (Heat shock protein HtpG) (High temperature protein G)
BCO26_2151	-	2.9273	0.0000	NADH:flavin oxidoreductase/NADH oxidase
BCO26_0934	-	2.9246	0.0000	ATPase AAA-2 domain protein
BCO26_1052	-	2.9075	0.0038	Uncharacterized protein
BCO26_2898	-	2.9038	0.0000	Drug resistance transporter, EmrB/QacA subfamily
BCO26_2515	*argD*	2.9004	0.0742	Multifunctional fusion protein
BCO26_2685	-	2.8904	0.0001	Anion transporter
BCO26_2513	-	2.8499	0.0000	FAD-dependent pyridine nucleotide-disulfide oxidoreductase
BCO26_0308	-	2.8427	0.0003	Metal dependent phosphohydrolase
BCO26_0586	-	2.8244	0.0002	BAAT/Acyl-CoA thioester hydrolase
BCO26_2416	*yfkM*	2.8229	0.0000	Intracellular protease, PfpI family
BCO26_0589	-	2.8102	0.0000	Aldo/keto reductase
BCO26_0735	-	2.7917	0.0001	Phosphotransferase system, phosphocarrier protein HPr
BCO26_1709	-	2.7618	0.0001	Nucleotidase (EC 3.1.3.-)
BCO26_0496	*metC*	2.7363	0.0000	Cystathionine gamma-synthase
BCO26_0298	*glpK*	2.6713	0.0032	Glycerol kinase
BCO26_1429	-	2.6689	0.0000	3-hydroxyisobutyrate dehydrogenase
BCO26_1771	-	2.6197	0.0095	Heat shock protein Hsp20
BCO26_1118	*sucD*	2.5942	0.0000	Succinate--CoA ligase [ADP-forming] subunit alpha (EC 6.2.1.5) (Succinyl-CoA synthetase subunit alpha) (SCS-alpha)
BCO26_2126	-	2.5806	0.0032	PTS system, glucose subfamily, IIA subunit
BCO26_0653	-	2.5640	0.0020	YvyF
BCO26_2482	*yhdN*	2.5571	0.0000	Aldo/keto reductase
BCO26_0731	-	2.5548	0.0000	NUDIX hydrolase
BCO26_1746	*dnaK*	2.5337	0.0000	Chaperone protein DnaK (HSP70) (Heat shock 70 kDa protein) (Heat shock protein 70)
BCO26_2725	*groES*	2.5200	0.0153	10 kDa chaperonin (GroES protein) (Protein Cpn10)
BCO26_2167	-	2.4931	0.0873	Peptidase M17 leucyl aminopeptidase domain protein
BCO26_0541	*ptnA*	2.4777	0.0000	PTS system, mannose/fructose/sorbose family, IIA subunit

BCO26_2743	*rsbV*	2.4404	0.0001	Anti-sigma factor antagonist
BCO26_1223	*mutL*	2.4133	0.0002	DNA mismatch repair protein MutL
BCO26_0186	*cobB*	2.4064	0.0012	NAD-dependent protein deacetylase (EC 3.5.1.-) (Regulatory protein SIR2 homolog)
BCO26_0660	-	2.4053	0.0000	Flagellin
BCO26_0420	*galR*	2.3949	0.0029	Transcriptional regulator, LacI family
BCO26_0213	-	2.3943	0.0030	Flavodoxin/nitric oxide synthase
BCO26_2103	-	2.3803	0.0026	Uncharacterized protein
BCO26_1978	-	2.3657	0.0001	UPF0173 metal-dependent hydrolase BCO26_1978
BCO26_0259	-	2.3632	0.0000	NAD-dependent epimerase/dehydratase
BCO26_0874	-	2.3489	0.0000	Uncharacterized protein
BCO26_0832	*spxA*	2.3374	0.0170	Regulatory protein Spx
BCO26_0587	-	2.3109	0.0041	Esterase/lipase-like protein
BCO26_1784	-	2.2983	0.0001	Uncharacterized protein
BCO26_2957	*ribH*	2.2752	0.0011	6,7-dimethyl-8-ribityllumazine synthase (DMRL synthase) (LS) (Lumazine synthase) (EC 2.5.1.78)
BCO26_0402	*yfmJ*	2.2720	0.0000	Alcohol dehydrogenase zinc-binding domain protein
BCO26_1841	*yrbC*	2.2719	0.0000	Probable transcriptional regulatory protein BCO26_1841
BCO26_1768	-	2.2708	0.0020	Glycolate oxidase, subunit GlcD
BCO26_2495	-	2.2634	0.0000	Nitroreductase
BCO26_1789	-	2.2622	0.0000	Peroxiredoxin
BCO26_0938	-	2.2612	0.0032	Uncharacterized protein
BCO26_2307	-	2.2471	0.0001	Ferric uptake regulator, Fur family
BCO26_1359	*plsY*	2.2396	0.0001	Glycerol-3-phosphate acyltransferase (Acyl-PO4 G3P acyltransferase) (Acyl-phosphate--glycerol-3-phosphate acyltransferase) (G3P acyltransferase) (GPAT) (EC 2.3.1.n3) (Lysophosphatidic acid synthase) (LPA synthase)
BCO26_0780	*yhgD*	2.2154	0.0005	Transcriptional regulator, TetR family
BCO26_1977	-	2.2027	0.0057	Putative signal transduction protein with CBS and DRTGG domains
BCO26_0622	*galK*	2.2010	0.0016	Galactokinase (EC 2.7.1.6) (Galactose kinase)
BCO26_2021	-	2.1981	0.0057	Molybdenum ABC transporter, periplasmic molybdate-binding protein
BCO26_1454	*selD*	2.1598	0.0000	Selenide, water dikinase (EC 2.7.9.3) (Selenium donor protein) (Selenophosphate synthase)
BCO26_1764	*aroE*	2.1254	0.0024	Shikimate dehydrogenase (NADP(+)) (SDH) (EC 1.1.1.25)
BCO26_0165	*cydB*	2.1109	0.0004	Cytochrome d ubiquinol oxidase, subunit II
BCO26_1679	-	2.0960	0.0000	Vitamin B12-dependent ribonucleotide reductase (EC 1.17.4.1)
BCO26_0263	-	2.0749	0.0000	Aminotransferase class I and II
BCO26_0171	-	2.0426	0.0001	Arginase (EC 3.5.3.1)

BCO26_2374	-	2.0188	0.0003	Methionine synthase vitamin-B12 independent
BCO26_2212	-	2.0129	0.0000	Catalase (EC 1.11.1.6)
BCO26_1539	-	2.0036	0.0000	Peptidase membrane zinc metallopeptidase putative
BCO26_1117	*sucC*	1.9921	0.0005	Succinate--CoA ligase [ADP-forming] subunit beta (EC 6.2.1.5) (Succinyl-CoA synthetase subunit beta) (SCS-beta)
BCO26_0544	-	1.9870	0.0002	Uncharacterized protein
BCO26_2279	-	1.9664	0.0042	Extracellular solute-binding protein family 3
BCO26_2070	*ytmA*	1.9553	0.0001	BAAT/Acyl-CoA thioester hydrolase
BCO26_1964	-	1.9500	0.0016	Citrate synthase
BCO26_0190	-	1.9480	0.0003	Methyl-accepting chemotaxis sensory transducer with Cache sensor
BCO26_2636	-	1.9436	0.0000	Diacylglycerol kinase catalytic region
BCO26_2370	-	1.9399	0.0006	Anti-sigma-factor antagonist
BCO26_0767	*yhaH*	1.9251	0.0000	Uncharacterized protein
BCO26_2181	*lipA*	1.9101	0.0002	Lipoyl synthase (EC 2.8.1.8)
BCO26_0434	-	1.8896	0.0002	RmlC-like cupin
BCO26_2878	-	1.8706	0.0000	Cys-tRNA(Pro)/Cys-tRNA(Cys) deacylase (EC 4.2.-.-)
BCO26_0543	*manN*	1.8642	0.0000	PTS system, mannose/fructose/sorbose family, IID subunit
BCO26_2303	-	1.8491	0.0000	Acetolactate synthase, catabolic
BCO26_0306	*yqiG*	1.8249	0.0000	NADH:flavin oxidoreductase/NADH oxidase
BCO26_2556	-	1.8216	0.0001	Heavy metal translocating P-type ATPase
BCO26_2595	*deoC*	1.8132	0.0000	Deoxyribose-phosphate aldolase

*Log_2FC：the logarithm of fold change, **Q-value：adjustment of *P*-value.

Supplementary Table 4. Down-regulated proteins in *B. coagulans* 2-6 by iTRAQ

Locus	Gene	log_2FC*	Q-value**	Description
BCO26_2160	-	-7.5824	0.0058	Thioesterase superfamily protein
BCO26_0352	-	-7.5092	0.0001	dTDP-4-dehydrorhamnose reductase
BCO26_0361	-	-6.9636	0.3778	Uncharacterized protein
BCO26_2488	*ureG*	-6.7583	0.0001	Urease accessory protein UreG
BCO26_0519	-	-6.6545	0.0003	Cell envelope-related transcriptional attenuator
BCO26_2428	-	-6.4803	0.0000	Band 7 protein
BCO26_2275	*queG*	-6.4136	0.0190	Epoxyqueuosine reductase
BCO26_0819	-	-5.9874	0.0077	Oligopeptide/dipeptide ABC transporter, ATPase subunit
BCO26_1048	*pyrD*	-5.9114	0.0000	Dihydroorotate dehydrogenase
BCO26_0034	*spoV G*	-5.7888	0.0000	Putative septation protein SpoVG (Stage V sporulation protein G)
BCO26_2663	*purS*	-5.6376	0.0001	Phosphoribosylformylglycinamidine synthase subunit PurS (FGAM synthase)
BCO26_2368	*ilvD*	-5.5863	0.0004	Dihydroxy-acid dehydratase (DAD) (EC 4.2.1.9)
BCO26_1650	-	-5.5171	0.0000	Hemolysin A
BCO26_2174	-	-5.4611	0.0003	Uncharacterized protein
BCO26_2285	*pflA*	-5.3524	0.0001	Pyruvate formate-lyase-activating enzyme (EC 1.97.1.4)
BCO26_1607	*spoII AB*	-5.0539	0.0028	Anti-sigma F factor (EC 2.7.11.1) (Stage II sporulation protein AB)
BCO26_1393	*yfiR*	-4.9993	0.0045	Transcriptional regulator, TetR family
BCO26_0894	*yfjA*	-4.8611	0.0008	ESAT-6-like protein
BCO26_1282	-	-4.8083	0.0000	UPF0176 protein BCO26_1282
BCO26_0828	-	-4.7767	0.0000	Binding-protein-dependent transport systems inner membrane component
BCO26_1240	-	-4.7517	0.0001	ABC transporter related protein
BCO26_1484	*cspD*	-4.7356	0.0002	Cold-shock DNA-binding domain protein
BCO26_1941	-	-4.6901	0.0043	2-dehydropantoate 2-reductase (EC 1.1.1.169) (Ketopantoate reductase)
BCO26_1608	-	-4.6612	0.0000	Anti-sigma F factor antagonist (Stage II sporulation protein)
BCO26_1403	-	-4.6556	0.0012	Transcriptional regulator, MerR family
BCO26_2618	-	-4.6063	0.0002	Terpenoid cyclases/Protein prenyltransferase
BCO26_1933	-	-4.5611	0.0000	tRNA/rRNA methyltransferase (SpoU)
BCO26_2665	-	-4.5541	0.0001	Adenylosuccinate lyase (ASL) (EC 4.3.2.2) (Adenylosuccinase)
BCO26_1864	-	-4.5241	0.0064	Uncharacterized protein
BCO26_0555	-	-4.4862	0.0001	ABC transporter related protein
BCO26_0347	-	-4.3992	0.0004	Putative glycosyl transferase
BCO26_0280	-	-4.3586	0.0001	TPR-like protein
BCO26_2119	-	-4.3128	0.0000	Polysaccharide deacetylase
BCO26_2310	-	-4.2956	0.0278	ATP/cobalamin adenosyltransferase
BCO26_1507	*xpt*	-4.2842	0.0002	Xanthine phosphoribosyltransferase (XPRTase)

				(EC 2.4.2.22)
BCO26_0443	*ypjH*	-4.2763	0.0000	Glycerol dehydrogenase
BCO26_2156	-	-4.2631	0.0000	Peptidyl-prolyl cis-trans isomerase (PPIase) (EC 5.2.1.8)
BCO26_1894	*ysoA*	-4.1856	0.0001	TPR-like protein
BCO26_0075	-	-4.1131	0.0001	Deoxynucleoside kinase
BCO26_0584	-	-4.1129	0.0000	Cof-like hydrolase
BCO26_1556	*ndk*	-4.0916	0.0915	Nucleoside diphosphate kinase (NDK) (NDP kinase) (EC 2.7.4.6) (Nucleoside-2-P kinase)
BCO26_1044	*pyrC*	-4.0482	0.0004	Dihydroorotase (DHOase) (EC 3.5.2.3)
BCO26_1036	-	-4.0019	0.0027	RNA-binding S4 domain protein
BCO26_1043	*pyrB*	-3.9945	0.0000	Aspartate carbamoyltransferase (EC 2.1.3.2) (Aspartate transcarbamylase) (ATCase)
BCO26_1856	*minC*	-3.9420	0.0001	Probable septum site-determining protein MinC
BCO26_2364	*leuA*	-3.8793	0.0752	2-isopropylmalate synthase (EC 2.3.3.13) (Alpha-IPM synthase) (Alpha-isopropylmalate synthase)
BCO26_0567	-	-3.8746	0.0001	Aldehyde-alcohol dehydrogenase
BCO26_1371	-	-3.8619	0.0029	2-nitropropane dioxygenase NPD
BCO26_0726	-	-3.8503	0.0000	HAD-superfamily hydrolase, subfamily IA, variant 1
BCO26_2917	-	-3.8472	0.0001	TIGR00697: conserved hypothetical integral
BCO26_0670	-	-3.7142	0.0003	Gamma-glutamyltransferase
BCO26_0343	-	-3.7141	0.0002	NAD-dependent epimerase/dehydratase
BCO26_1529	*panC*	-3.6906	0.0001	Pantothenate synthetase (PS) (EC 6.3.2.1) (Pantoate--beta-alanine ligase) (Pantoate-activating enzyme)
BCO26_2768	*malL*	-3.6481	0.0000	Oligo-1,6-glucosidase
BCO26_1408	*acoC*	-3.6025	0.0162	Dihydrolipoamide acetyltransferase component of pyruvate dehydrogenase complex (EC 2.3.1.-)
BCO26_1049	*pyrF*	-3.5786	0.0008	Orotidine 5'-phosphate decarboxylase (EC 4.1.1.23) (OMP decarboxylase) (OMPDCase) (OMPdecase)
BCO26_1045	*carA*	-3.5253	0.0000	Carbamoyl-phosphate synthase small chain (EC 6.3.5.5) (Carbamoyl-phosphate synthetase glutamine chain)
BCO26_1173	*rimP*	-3.4935	0.0012	Ribosome maturation factor RimP
BCO26_0829	*oppC*	-3.4615	0.0000	Binding-protein-dependent transport systems inner membrane component
BCO26_1722	-	-3.4578	0.0028	Uncharacterized protein
BCO26_1652	-	-3.4278	0.0087	Polyprenyl synthetase
BCO26_2619	-	-3.4053	0.0003	Uncharacterized protein
BCO26_2545	-	-3.3487	0.0322	Type I site-specific deoxyribonuclease, HsdR family
BCO26_0296	-	-3.3480	0.0006	Glycerol uptake operon antiterminator regulatory protein
BCO26_0202	-	-3.3374	0.0006	Glycine betaine/L-proline ABC transporter, ATPase subunit

BCO26_1166	*uppS*	-3.3127	0.0014	Isoprenyl transferase (EC 2.5.1.-)
BCO26_1632	-	-3.2619	0.0023	CheW protein
BCO26_0871	-	-3.2586	0.0004	ABC transporter related protein
BCO26_1040	*lspA*	-3.2580	0.0002	Lipoprotein signal peptidase (EC 3.4.23.36) (Prolipoprotein signal peptidase) (Signal peptidase II) (SPase II)
BCO26_1384	*thiE*	-3.2397	0.0000	Thiamine-phosphate synthase (TP synthase) (TPS) (EC 2.5.1.3) (Thiamine-phosphate pyrophosphorylase) (TMP pyrophosphorylase) (TMP-PPase)
BCO26_1942	-	-3.1936	0.0043	NADPH-dependent FMN reductase
BCO26_1831	*yrzD*	-3.1820	0.0392	Uncharacterized protein
BCO26_1720	*ispH*	-3.1819	0.0004	4-hydroxy-3-methylbut-2-enyl diphosphate reductase (EC 1.17.7.4)
BCO26_0831	-	-3.1643	0.0001	ABC transporter related protein
BCO26_0678	-	-3.1311	0.0000	Peptidase M23
BCO26_0568	*deaD*	-3.1212	0.0000	DEAD/DEAH box helicase domain protein
BCO26_1386	*yflK*	-3.1057	0.0050	MOSC domain containing protein
BCO26_2362	*leuC*	-3.1016	0.0001	3-isopropylmalate dehydratase large subunit (EC 4.2.1.33) (Alpha-IPM isomerase) (IPMI) (Isopropylmalate isomerase)
BCO26_2873	-	-3.0291	0.0000	Uncharacterized protein
BCO26_0174	*ybbM*	-3.0113	0.0027	Putative transmembrane anti-sigma factor
BCO26_1761	-	-2.9811	0.0247	Metal dependent phosphohydrolase
BCO26_0532	-	-2.9766	0.0004	3D domain protein
BCO26_1544	*qcrA*	-2.8935	0.0102	Rieske (2Fe-2S) domain protein
BCO26_2123	-	-2.8916	0.0000	Rhodanese domain protein
BCO26_2657	*purH*	-2.8563	0.0008	Bifunctional purine biosynthesis protein PurH [Includes: IMP cyclohydrolase (EC 3.5.4.10) (IMP synthase) (Inosinicase) (ATIC); Phosphoribosylaminoimidazolecarboxamide formyltransferase (EC 2.1.2.3) (AICAR transformylase)]
BCO26_1657	*nusB*	-2.8266	0.0001	N utilization substance protein B homolog (Protein NusB)
BCO26_2903	*deoC*	-2.8003	0.0000	Deoxyribose-phosphate aldolase (DERA) (EC 4.1.2.4) (2-deoxy-D-ribose 5-phosphate aldolase) (Phosphodeoxyriboaldolase) (Deoxyriboaldolase)
BCO26_1314	*msrA*	-2.7925	0.0000	Peptide methionine sulfoxide reductase MsrA (Protein-methionine-S-oxide reductase) (EC 1.8.4.11) (Peptide-methionine (S)-S-oxide reductase) (Peptide Met(O) reductase)
BCO26_0638	-	-2.7451	0.0000	Uncharacterized protein
BCO26_1200	-	-2.7242	0.0001	Inner-membrane translocator
BCO26_0203	-	-2.7112	0.0000	Substrate-binding region of ABC-type glycine betaine transport system
BCO26_1992	-	-2.6967	0.0000	AMP-dependent synthetase and ligase
BCO26_2924	-	-2.6938	0.0002	Carboxynorspermidine decarboxylase

BCO26_0811	-	-2.6897	0.0007	Uncharacterized protein
BCO26_0872	*yhaP*	-2.6678	0.0003	ABC-2 type transporter
BCO26_2688	-	-2.6461	0.0000	Uncharacterized protein
BCO26_0830	-	-2.6101	0.0000	Oligopeptide/dipeptide ABC transporter, ATPase subunit
BCO26_1497	*ynzC*	-2.6075	0.0001	UPF0291 protein BCO26_1497
BCO26_0728	-	-2.5846	0.0022	Tetratricopeptide TPR_2 repeat protein
BCO26_2922	-	-2.5739	0.0001	Anti-sigma-factor antagonist
BCO26_2664	*purC*	-2.5465	0.0000	Phosphoribosylaminoimidazole-succinocarboxamide synthase (EC 6.3.2.6) (SAICAR synthetase)
BCO26_2491	*ureC*	-2.5391	0.0001	Urease subunit alpha (EC 3.5.1.5) (Urea amidohydrolase subunit alpha)
BCO26_0304	-	-2.5149	0.0000	Uncharacterized protein
BCO26_2547	-	-2.4971	0.0004	Type I restriction-modification system, M subunit
BCO26_1936	-	-2.4754	0.0001	Uncharacterized protein
BCO26_2592	*guaC*	-2.4506	0.0000	GMP reductase (EC 1.7.1.7) (Guanosine 5'-monophosphate oxidoreductase) (Guanosine monophosphate reductase)
BCO26_1797	*udk*	-2.4486	0.0129	Uridine kinase (EC 2.7.1.48) (Cytidine monophosphokinase) (Uridine monophosphokinase)
BCO26_1601	-	-2.4359	0.0000	Diaminopimelate decarboxylase (EC 4.1.1.20)
BCO26_2821	-	-2.4351	0.0008	Response regulator receiver protein
BCO26_0554	-	-2.4201	0.0002	NMT1/THI5 like domain protein
BCO26_2649	*pcrA*	-2.4122	0.0003	DNA helicase (EC 3.6.4.12)
BCO26_1775	-	-2.4005	0.0007	SirA family protein
BCO26_0345	-	-2.3925	0.0003	Putative lipopolysaccharide biosynthesis protein
BCO26_1046	*carB*	-2.3867	0.0001	Carbamoyl-phosphate synthase large chain (EC 6.3.5.5) (Carbamoyl-phosphate synthetase ammonia chain)
BCO26_2739	-	-2.3688	0.0000	RNA binding S1 domain protein
BCO26_2384	-	-2.3454	0.0000	Chromosome segregation ATPase-like protein
BCO26_2950	*dnaC*	-2.3363	0.0042	Replicative DNA helicase (EC 3.6.4.12)
BCO26_2606		-2.3146	0.0003	Uncharacterized protein
BCO26_1890	*lonA lon*	-2.3133	0.0000	Lon protease (EC 3.4.21.53) (ATP-dependent protease La)
BCO26_0057	*hprT*	-2.2931	0.0000	Hypoxanthine phosphoribosyltransferase
BCO26_1527	-	-2.2737	0.0126	DnaQ family exonuclease/DinG family helicase
BCO26_1243	-	-2.2705	0.0000	Uncharacterized protein
BCO26_0391	-	-2.2550	0.0013	Arabinogalactan endo-beta-1,4-galactanase (EC 3.2.1.89)
BCO26_2814	*tdk*	-2.2521	0.0000	Thymidine kinase (EC 2.7.1.21)
BCO26_0232	-	-2.2426	0.0001	Periplasmic binding protein
BCO26_0348	-	-2.2171	0.0002	NAD-dependent epimerase/dehydratase
BCO26_1953	*dnaB*	-2.2143	0.0031	Replication initiation and membrane

				attachment family protein
BCO26_2386	-	-2.2108	0.0136	Uncharacterized protein
BCO26_1435	-	-2.2091	0.0004	DSBA oxidoreductase
BCO26_1927	-	-2.1915	0.0004	Cell division protein ZapA
BCO26_1230	*hflX*	-2.1667	0.0000	GTPase HflX (GTP-binding protein HflX)
BCO26_2476	*rlmN*	-2.1596	0.0000	Probable dual-specificity RNA methyltransferase RlmN (EC 2.1.1.192) (23S rRNA (adenine(2503)-C(2))-methyltransferase) (23S rRNA m2A2503 methyltransferase) (Ribosomal RNA large subunit methyltransferase N) (tRNA (adenine(37)-C(2))-methyltransferase) (tRNA m2A37 methyltransferase)
BCO26_2304	-	-2.1399	0.0008	Transcriptional regulator, LysR family
BCO26_2608	-	-2.1276	0.0236	Urea amidolyase related protein
BCO26_2644	-	-2.1254	0.0003	Malate synthase (EC 2.3.3.9)
BCO26_1050	*pyrE*	-2.1166	0.0000	Orotate phosphoribosyltransferase (OPRT) (OPRTase) (EC 2.4.2.10)
BCO26_2693	-	-2.1107	0.0000	Transposase IS4 family protein
BCO26_0427	-	-2.0539	0.0000	Transferase hexapeptide repeat containing protein
BCO26_0083	-	-2.0469	0.0021	Uncharacterized protein
BCO26_1810	*mnmA*	-2.0469	0.0017	tRNA-specific 2-thiouridylase MnmA (EC 2.8.1.13)
BCO26_2648	*pcrA*	-2.0207	0.0001	DNA helicase (EC 3.6.4.12)
BCO26_1922	-	-2.0203	0.0003	Transcriptional regulator, TetR family
BCO26_1101	*smc*	-2.0146	0.0000	Chromosome partition protein Smc
BCO26_1687	*aroK*	-2.0087	0.0011	Shikimate kinase (SK) (EC 2.7.1.71)
BCO26_1998	-	-1.9980	0.0005	Putative GAF sensor protein
BCO26_1636	*fruK*	-1.9887	0.0003	1-phosphofructokinase
BCO26_0019	-	-1.9715	0.0035	Methyltransferase small
BCO26_1509	-	-1.9706	0.0001	Putative RNA methylase
BCO26_2801	*upp*	-1.9023	0.0000	Uracil phosphoribosyltransferase (EC 2.4.2.9) (UMP pyrophosphorylase) (UPRTase)
BCO26_0267	-	-1.8989	0.0008	UPF0210 protein BCO26_0267
BCO26_1112	-	-1.8932	0.0001	Signal peptidase I (EC 3.4.21.89)
BCO26_2472	*yxeH*	-1.8477	0.0000	Cof-like hydrolase
BCO26_1008	*bshC*	-1.7849	0.0000	Putative cysteine ligase BshC (EC 6.-.-.-)

*Log_2FC: the logarithm of fold change, **Q-value: adjustment of *P*-value.

Supplementary Table 5. Commonly differentially accumulated in the RNA-seq and iTRAQ

Locus	Gene	$log_2(37/60)$	Product
BCO26_2925	-	5.96	hypothetical protein
BCO26_0880	*ykuS*	5.85	hypothetical protein
BCO26_1317	*cspL*	5.66	cold-shock DNA-binding domain-containing protein
BCO26_2080	*dps*	5.51	Ferritin dps family protein
BCO26_2638	*gsiB*	4.49	general stress protein
BCO26_2375	*ybfB*	4.24	major facilitator superfamily protein
BCO26_1438	*mntH*	4.14	Mn^{2+}/Fe^{2+} transporter, NRAMP family
BCO26_2932	-	3.50	malate/quinone oxidoreductase
BCO26_0241	*gabD*	3.50	succinic semialdehyde dehydrogenase
BCO26_2724	*groEL*	3.38	chaperonin GroEL
BCO26_1748	*hrcA*	3.05	heat-inducible transcription repressor HrcA
BCO26_0934	*clpE*	2.92	ATPase AAA-2 domain-containing protein
BCO26_1771	-	2.61	heat shock protein Hsp20
BCO26_1746	*dnaK*	2.53	chaperone protein DnaK
BCO26_2725	*groES*	2.51	chaperonin Cpn10
BCO26_0541	-	2.47	PTS system, mannose/fructose/sorbose family, IIA subunit
BCO26_2743	*rsbV*	2.44	anti-sigma-factor antagonist
BCO26_0660	*hag*	2.40	flagellin
BCO26_0780	*yhgD*	2.21	TetR family transcriptional regulator
BCO26_1679	-	2.09	adenosylcobalamin-dependent ribonucleoside-diphosphate reductase
BCO26_0171	*argI*	2.04	arginase
BCO26_1117	*sucC*	1.99	succinyl-CoA synthetase subunit beta
BCO26_1964	*citZ*	1.94	2-methylcitrate synthase/citrate synthase II
BCO26_2370	-	1.93	anti-sigma-factor antagonist
BCO26_0543	*levG*	1.86	PTS system, mannose/fructose/sorbose family, IID subunit
BCO26_0306	*yqiG*	1.82	NADH:flavin oxidoreductase/NADH oxidase
BCO26_2608	*kipA*	-2.12	urea amidolyase-like protein
BCO26_2664	*purC*	-2.54	phosphoribosylaminoimidazole-succinocarboxamide synthase
BCO26_0872	*natB*	-2.66	ABC-2 type transporter
BCO26_0639	*lytE*	-2.74	NLP/P60 protein
BCO26_0871	*natA*	-3.25	ABC transporter-like protein
BCO26_1529	*panC*	-3.69	pantoate/beta-alanine ligase
BCO26_0670	-	-3.71	gamma-glutamyltransferase
BCO26_0443	-	-4.27	glycerol dehydrogenase
BCO26_2665	*purB*	-4.55	adenylosuccinate lyase
BCO26_0828	-	-4.77	binding-protein-dependent transport systems inner membrane component
BCO26_2663	*purS*	-5.63	phosphoribosylformylglycinamidine synthase, purS
BCO26_2428	*yuaG*	-6.48	hypothetical protein

Supplementary Table 6. mRNA targets of CspL in *E. coli* DH5α by RIP-seq

Gene	Description	Foldchange	*P*-Value
acpP	acyl carrier protein (ACP)	3.209574	0.011899
acrE	cytoplasmic membrane lipoprotein	5.40998	0.033148
acrF	multidrug efflux system protein	3.447072	0.02446
actP	acetate transporter	5.017942	0.000673
ahpC	alkyl hydroperoxide reductase, C22 subunit	6.277318	0.00017
aslB	putative AslA-specific sulfatase-maturating enzyme	4.007795	0.010328
aspA	aspartate ammonia-lyase	3.189925	0.01204
atoB	acetyl-CoA acetyltransferase	3.056902	0.021741
atpC	F1 sector of membrane-bound ATP synthase, epsilon subunit	8.418546	1.64E-05
bamB	BamABCDE complex OM biogenesis lipoprotein	5.713884	0.000256
bax	putative glucosaminidase	4.753606	0.000945
bolA	stationary-phase morphogene, transcriptional repressor for mreB; also regulator for dacA, dacC, and ampC	3.849887	0.003751
bssS	biofilm regulator	2.773061	0.025726
chbC	N,N'-diacetylchitobiose-specific enzyme IIC component of PTS	4.538809	0.007841
citX	apo-citrate lyase phosphoribosyl-dephospho-CoA transferase	13.08699	0.000935
creC	sensory histidine kinase in two-component regulatory system with CreB or PhoB	3.46148	0.032593
cspC	stress protein, member of the CspA-family	5.556331	0.000546
cspE	constitutive cold shock family transcription antitermination protein; negative regulator of cspA transcription; RNA melting protein; ssDNA-binding protein	7.54401	4.64E-05
cutC	copper homeostasis protein	6.964267	6.17E-05
cynX	putative cyanate transporter	9.186281	0.00679
ddpC	D,D-dipeptide ABC transporter permease	8.085941	0.004943
diaA	DnaA initiator-associating factor for replication initiation	3.495332	0.019935
dtpC	dipeptide and tripeptide permease	3.676945	0.024268
dusB	tRNA-dihydrouridine synthase B	5.506834	0.00128
ecpA	ECP pilin	31.32438	3.33E-05
elyC	envelope biogenesis factor; DUF218 superfamily protein	3.134626	0.039049
eno	enolase	2.523128	0.042581
fabF	3-oxoacyl-[acyl-carrier-protein	3.019607	0.016262
fabZ	(3R)-hydroxymyristol acyl carrier protein dehydratase	3.161011	0.019625
fdhF	formate dehydrogenase-H, selenopolypeptide subunit	2.570259	0.041695
fdrA	putative NAD(P)-binding acyl-CoA synthetase	10.48652	0.003397
fetB	iron export ABC transporter permease; peroxide resistance protein	4.323343	0.010321
fhuA	ferrichrome outer membrane transporter	3.385106	0.009279
frdD	fumarate reductase (anaerobic), membrane anchor subunit	4.378659	0.003917
frlD	fructoselysine 6-kinase	4.350342	0.016253
frwD	putative enzyme IIB component of PTS	6.585809	0.029994
ftsB	cell division protein	2.929617	0.039821
fucI	L-fucose isomerase	3.423418	0.013042

gadX	acid resistance regulon transcriptional activator; autoactivator	3.434245	0.009939
gfcC	putative O-antigen capsule production periplasmic protein	6.585809	0.029994
glgS	motility and biofilm regulator	4.199755	0.002111
glnB	regulatory protein P-II for glutamine synthetase	4.187669	0.02311
glvBC	arbutin specific enzyme IIBC component of PTS	4.966913	0.020774
grcA	autonomous glycyl radical cofactor	3.77471	0.008271
greB	transcript cleavage factor	7.706497	0.001895
gspH	putative general secretory pathway component, cryptic	62.45439	0.002355
hns	global DNA-binding transcriptional dual regulator H-NS	7.787963	2.09E-05
hofB	T2SE secretion family protein; P-loop ATPase superfamily protein	3.192656	0.041713
hokD	Qin prophage; small toxic polypeptide	833.1739	2.46E-21
hpf	ribosome hibernation promoting factor HPF; stabilizes 100S dimers	3.038436	0.021803
hspQ	heat shock protein involved in degradation of mutant DnaA; hemimethylated oriC DNA-binding protein	2.819931	0.024334
hupB	HU, DNA-binding transcriptional regulator, beta subunit	8.143978	2.46E-05
hybD	maturation protease for hydrogenase 2	3.668497	0.01606
hyfG	hydrogenase 4, subunit	8.62491	9.06E-05
ibaG	acid stress protein; putative BolA family transcriptional regulator	6.585809	0.029994
ibsD	toxic membrane protein	5.40998	0.033148
insA-20	IS1 protein InsA	5.40998	0.033148
insF-2	IS3 element protein InsF	17.00581	3.62E-05
iscX	Fe(2+) donor and activity modulator for cysteine desulfurase	3.46148	0.032593
lpp	murein lipoprotein	20.37404	4.48E-09
lpxA	UDP-N-acetylglucosamine acetyltransferase	5.159657	0.001008
lpxB	tetraacyldisaccharide-1-P synthase	3.344291	0.018232
lpxC	UDP-3-O-acyl N-acetylglucosamine deacetylase	6.350676	0.000112
lpxD	UDP-3-O-(3-hydroxymyristoyl)-glucosamine N-acyltransferase	3.271445	0.013084
lspA	prolipoprotein signal peptidase (signal peptidase II)	4.139157	0.006016
marB	periplasmic mar operon regulator	12.14387	0.009409
mcrC	5-methylcytosine-specific restriction enzyme McrBC, subunit McrC	16.939	0.001795
mepS	murein DD-endopeptidase, space-maker hydrolase, mutational suppressor of prc thermosensitivity, outer membrane lipoprotein, weak murein LD-carboxypeptidase	3.217454	0.042785
metJ	transcriptional repressor, S-adenosylmethionine-binding	3.84058	0.034643
mlaA	ABC transporter maintaining OM lipid asymmetry, OM lipoprotein component	2.742079	0.031826
mltD	putative membrane-bound lytic murein transglycosylase D	6.158456	0.000168
mokB	regulatory peptide	3.337777	0.024422
mokC	regulatory protein for HokC	62.45439	0.002355
mreC	cell wall structural complex MreBCD transmembrane component MreC	3.16955	0.021326
mreD	cell wall structural complex MreBCD transmembrane component MreD	4.623171	0.02182

nadB	quinolinate synthase, L-aspartate oxidase (B protein) subunit	2.9327	0.028079
nagC	N-acetylglucosamine-inducible nag divergent operon transcriptional repressor	2.972479	0.025544
nrdH	hydrogen donor for NrdEF electron transport system; glutaredoxin-like protein	4.796089	0.034567
osmE	osmotically-inducible lipoprotein	8.729785	1.06E-05
pcnB	poly(A) polymerase	2.971236	0.048139
pdhR	pyruvate dehydrogenase complex repressor; autorepressor	2.719794	0.038463
pgaB	poly-beta-1,6-N-acetyl-D-glucosamine (PGA) N-deacetylase outer membrane export lipoprotein	3.84058	0.034643
pgrR	murein peptide degradation regulator	3.318453	0.046767
pitB	phosphate transporter	5.51483	0.012477
pnp	polynucleotide phosphorylase/polyadenylase	4.463405	0.001525
priB	primosomal protein N	7.538298	0.000998
purA	adenylosuccinate synthetase	2.748753	0.034027
rhsB	Rhs protein with DUF4329 family putative toxin domain; putative neighboring cell growth inhibitor	3.246389	0.024304
rmf	ribosome modulation factor	51.17602	8.01E-13
rof	modulator of Rho-dependent transcription termination	2.987261	0.019071
rplA	50S ribosomal subunit protein L1	2.731246	0.030338
rplI	50S ribosomal subunit protein L9	5.732205	0.000532
rplK	50S ribosomal subunit protein L11	2.582293	0.04626
rplQ	50S ribosomal subunit protein L17	4.581168	0.001313
rplT	50S ribosomal subunit protein L20	4.593493	0.001203
rpmA	50S ribosomal subunit protein L27	2.687881	0.035911
rpmB	50S ribosomal subunit protein L28	5.541826	0.00872
rpmG	50S ribosomal subunit protein L33	8.80233	0.000807
rpmI	50S ribosomal subunit protein L35	4.446064	0.003241
rpoA	RNA polymerase, alpha subunit	2.493898	0.045829
rpoE	RNA polymerase sigma E factor	3.447987	0.00876
rpsB	30S ribosomal subunit protein S2	3.134611	0.013402
rpsF	30S ribosomal subunit protein S6	4.51296	0.004869
rpsK	30S ribosomal subunit protein S11	2.499139	0.0493
rpsO	30S ribosomal subunit protein S15	3.409615	0.008777
rpsQ	30S ribosomal subunit protein S17	3.992406	0.004247
rpsR	30S ribosomal subunit protein S18	12.14387	0.009409
rpsT	30S ribosomal subunit protein S20	6.177334	0.000578
rpsU	30S ribosomal subunit protein S21	13.34723	4.18E-07
rraA	ribonuclease E (RNase E) inhibitor protein	3.913945	0.003444
rsmE	16S rRNA m(3)U1498 methyltransferase, SAM-dependent	4.760002	0.008624
rspB	putative Zn-dependent NAD(P)-binding oxidoreductase	3.044887	0.034497
ryfB	hypothetical protein	6.352104	0.002965
rzpD	DLP12 prophage; putative murein endopeptidase	4.418997	0.035098
secE	preprotein translocase membrane subunit	3.01071	0.026356
secG	preprotein translocase membrane subunit	8.602427	6.06E-05
sieB	phage superinfection exclusion protein, Rac prophage	3.941004	0.014502

skp	periplasmic chaperone	3.276121	0.012614
ssb	single-stranded DNA-binding protein	4.722417	0.001074
symE	toxic peptide regulated by antisense sRNA symR	5.51483	0.012477
tdcA	tdc operon transcriptional activator	7.886045	0.014032
torY	TMAO reductase III (TorYZ), cytochrome c-type subunit	3.415476	0.02446
trxA	thioredoxin 1	6.090052	0.000161
uxuB	D-mannonate oxidoreductase, NAD-dependent	2.959111	0.029658
vioB	VioB, involved in dTDP-N-acetylviosamine synthesis	3.51241	0.032995
waaV	putative beta1,3-glucosyltransferase	3.267396	0.030569
wcaD	putative colanic acid polymerase	17.00581	3.62E-05
xthA	exonuclease III	5.962049	0.000976
xylE	D-xylose transporter	2.712545	0.036868
yaaW	UPF0174 family protein	4.187669	0.02311
yadV	putative periplasmic pilin chaperone	7.886045	0.014032
ybcJ	ribosome-associated protein; putative RNA-binding protein	15.29454	3.15E-06
ybcK	DLP12 prophage; putative recombinase	6.206146	0.002108
ybgF	periplasmic TolA-binding protein	2.643229	0.0376
ybjS	putative NAD(P)H-dependent oxidoreductase	2.741351	0.030532
yceD	DUF177 family protein	3.246461	0.021017
ycgI	hypothetical protein	9.746308	0.023263
ycgX	DUF1398 family protein	5.16591	0.009699
yciC	UPF0259 family inner membrane protein	2.94429	0.04277
yciY	uncharacterized protein	42.77316	1.42E-09
ydcC	H repeat-associated putative transposase	5.40998	0.033148
ydcH	DUF465 family protein	2.735667	0.029241
ydeQ	putative fimbrial-like adhesin protein	62.45439	0.002355
ydfK	cold shock protein, function unknown, Qin prophage	3.27404	0.011718
ydhZ	uncharacterized protein	3.093031	0.037005
ydjH	putative kinase	9.746308	0.023263
yebO	putative inner membrane protein	2.834947	0.033672
yecH	DUF2492 family protein	3.603295	0.019449
yecT	uncharacterized protein	3.979716	0.034967
yegK	ser/thr phosphatase-related protein	4.534759	0.015553
yfbR	5'-nucleotidase	4.163844	0.035158
yfcJ	putative arabinose efflux transporter	10.33865	4.40E-05
yfcZ	UPF0381 family protein	14.93671	1.38E-06
yfeD	DUF1323 family putative DNA-binding protein	2.764708	0.028824
ygbA	uncharacterized protein	3.316346	0.024402
ygdR	DUF903 family verified lipoprotein	22.50025	5.14E-07
ygfK	putative Fe-S subunit oxidoreductase subunit	7.191291	0.000108
yggD	MtlR family putative transcriptional repressor	3.561411	0.016737
yggI	Zn-dependent metalloprotease-related protein	4.375114	0.022569
yggS	UPF0001 family protein, PLP-binding	3.818282	0.018485
yghR	putative ATP-binding protein	93.18158	0.000319
yghS	putative ATP-binding protein	16.939	0.001795

ygiN	quinol monooxygenase	3.899535	0.004836
ygiZ	inner membrane protein	7.538298	0.000998
ygjI	putative transporter	7.193954	0.009097
yhaB	uncharacterized protein	4.418997	0.035098
yhfX	putative pyridoxal 5'-phosphate binding protein	9.746308	0.023263
yhiKL	hypothetical protein	7.379136	0.001624
yiaB	YiaAB family inner membrane protein	9.746308	0.023263
yifE	UPF0438 family protein	5.89942	0.000473
yihD	DUF1040 protein YihD	4.968943	0.007961
yihL	putative DNA-binding transcriptional regulator	11.78675	0.001755
yjbE	extracellular polysaccharide production threonine-rich protein	16.939	0.001795
yjbJ	stress-induced protein, UPF0337 family	3.500016	0.006655
yjbL	uncharacterized protein	12.14387	0.009409
yjhX	UPF0386 family protein	13.08699	0.000935
yjjV	putative DNase	3.246266	0.030295
ykiA	hypothetical protein	4.619387	0.006433
ymdF	KGG family protein	4.678155	0.007337
ymdF	KGG family protein	3.863167	0.003654
ynaE	cold shock protein, Rac prophage	3.27404	0.011718
ynaJ	DUF2534 family putative inner membrane protein	8.532225	1.26E-05
yneL	putative transcriptional regulator	77.81799	0.000833
yniA	fructosamine kinase family protein	3.659973	0.005595
ynjD	putative ABC transporter ATPase	10.44608	0.000248
ynjI	inner membrane protein	13.08699	0.000935
yqfA	hemolysin III family HylIII inner membrane protein	2.615779	0.03955
yqiA	acyl CoA esterase	3.46148	0.032593
yqiB	DUF1249 protein YqiB	3.447072	0.02446
yraH	putative fimbrial-like adhesin protein	4.406606	0.004523
yraJ	putative outer membrane protein	13.59433	3.22E-07
yrbL	Mg(2+)-starvation-stimulated protein	3.052986	0.034929
ytfK	DUF1107 family protein	3.931535	0.00317
ytfP	GGCT-like protein	3.818045	0.004559
yzfA	hypothetical protein	16.98769	0.000167
zapA	FtsZ stabilizer	8.311171	1.39E-05
ECD_03695	Magnesium and cobalt transport protein corA	3.84058	0.034643
ECD_00840	hypothetical protein	3.379743	0.031841
ECD_03786	putative glycoporin	3.085895	0.02023
ECD_02855	hypothetical protein	3.113105	0.013798
ECD_02652	hypothetical protein	5.51483	0.012477
ECD_02621	hypothetical protein	3.595737	0.011338
ECD_00022	hypothetical protein	12.14387	0.009409
ECD_04314	hypothetical protein	4.474227	0.006136
ECD_00815	integrase for prophage	5.730951	0.000523
ECD_03459	hypothetical protein	93.18158	0.000319

RIP-seq at 45 °C			
aaeR	LysR-typ	2.70218	0.002386
abgR	putative	4.02184	0.000685
acnA	aconitat	2.65249	0.001428
acpT	holo-[ac	3.29464	0.001157
acrF	multidru	4.15713	0.00029
acrR	DNA-bind	2.78407	0.003
adiA	arginine	2.40171	0.005009
adiC	arginine	3.06149	0.00159
adk	adenylat	2.64996	0.003808
agaB	galactos	2.59212	0.003396
agaI	putative	2.40951	0.007147
ahpC	alkyl hy	3.13729	0.001461
aidB	putative	3.53302	0.002347
aldA	aldehyde	3.4241	0.001582
alkB	DNA oxid	3.90966	0.00056
allC	allantoa	4.81374	0.000278
allE	(S)-urei	3.96882	0.000801
alsA	D-allose	3.36181	0.000939
alsB	D-allose	3.93246	0.001152
alsE	D-allulo	2.02525	0.019469
ampE	protein	2.47811	0.005142
ampG	muropept	2.2025	0.008454
ansB	L-aspara	3.3805	0.001263
araJ	putative	4.73139	0.000139
arnB	UDP-4-am	3.06372	0.000651
arnT	lipid IV	2.67612	0.004389
aroK	shikimat	2.97643	0.001798
aroL	shikimat	5.06518	0.000299
arpA	regulato	2.9345	0.002394
arpB	EcoGene:	3.34382	0.000953
artJ	L-argini	3.38676	0.001889
artP	L-argini	2.75665	0.002606
ascG	DNA-bind	2.69596	0.002518
asnU	EcoGene:	3.34195	0.001145
asnV	EcoGene:	2.39627	0.003705
atoB	acetyl-C	3.14662	0.001326
atoC	DNA-bind	3.63521	0.000815
atoS	sensory	2.78907	0.002241
bacA	undecapr	2.57686	0.003297
barA	sensory	2.88664	0.001586
bcsE	c-di-GMP	2.73256	0.003566
betT	choline:	2.93847	0.001416
bglB	6-phosph	2.52141	0.007533
bglF	beta-glu	2.46536	0.005557

bglH	carbohyd		3.66982	0.000822
bglJ	DNA-bind		2.11218	0.014947
birA	DNA-bind		2.4006	0.004102
bisC	biotin s		4.80925	0.000369
blr	beta-lac		2.02835	0.01479
bolA	DNA-bind		2.36194	0.005649
bsmA	DUF1471		3.96098	0.00021
bssR	regulato		9.68467	3.62E-05
bssS	regulato		5.61678	7.05E-05
cadB	lysine:c		5.31585	0.000224
cadC	DNA-bind		4.1597	0.000466
caiB	gamma-bu		2.73003	0.001757
caiD	crotonob		2.88006	0.002891
caiE	putative		2.64433	0.004484
caiT	L-carnit		4.30431	0.000451
carA	carbamoy		2.79536	0.00266
carB	carbamoy		2.47381	0.008491
cbl	DNA-bind		4.07401	0.000808
cbrB	putative		4.38117	0.000736
cdgI	putative		2.073	0.018846
chaA	Na(+)/K(		4.38825	0.000578
chaB	putative		4.54168	0.000272
chbC	N,N'-dia		2.79104	0.006452
chbF	monoacet		3.98321	0.000681
cheR	chemotax		4.16165	0.000581
cheY	chemotax		5.25309	0.000204
cirA	ferric d		3.31654	0.001002
clsC	cardioli		3.82785	0.000814
cmtA		#N/A	3.58701	0.000659
coaD	pantethe		3.72575	0.000418
cobS	cobalami		2.84783	0.002597
codB	cytosine		2.72631	0.003585
creA	PF05981		3.19339	0.001958
csgC	inhibito		5.02313	0.000259
csiD	PF08943		3.17675	0.001703
cspH	CspA fam		2.60107	0.004479
csrA	carbon s		1.71929	0.02066
cueO	cuprous		4.44555	0.00069
curA	NADPH-de		5.66106	0.000254
cyuP	putative		2.42953	0.005765
dacC	D-alanyl		2.23972	0.015482
dacD	D-alanyl		2.05841	0.005227
dcuD	putative		2.15704	0.009668
dcuR	DNA-bind		3.35393	0.00119
ddpC	putative		2.916	0.001877

dedA	DedA fam	3.04368	0.00376
dgcE	putative	2.38774	0.008844
dgcP	diguanyl	2.89353	0.002796
dgcT	putative	4.18798	0.000354
dhaM	dihydrox	2.99837	0.00286
dinD	DNA dama	2.99214	0.002715
djlC	co-chape	2.24607	0.014512
dkgA	methylgl	3.02762	0.002004
dosP	oxygen-s	3.91871	0.002353
dpiB	sensory	2.64826	0.004124
dsbA	thiol:di	4.06533	0.000419
dsdA	D-serine	3.13886	0.001793
dsdX	D-serine	4.51171	0.000419
dtpC	dipeptid	2.9459	0.001551
eamB	cysteine	3.21816	0.001443
ebgA	evolved	2.45551	0.004941
ebgC	DUF386 d	3.48327	0.001275
ecpA	common p	1.79632	0.010079
efeB	heme-con	6.99246	3.67E-05
efeO	ferrous	2.12892	0.008518
elfC	putative	2.97661	0.003598
elfD	putative	3.35294	0.000921
emrE	multidru	3.30588	0.001783
emrY	triparti	2.27685	0.012723
envC	murein h	3.45236	0.000828
eptA	phosphoe	3.34937	0.001154
essD	DLP12 pr	3.58503	0.001005
evgA	DNA-bind	3.15432	0.001859
fadD	fatty ac	3.89773	0.000431
fadH	2,4-dien	1.92998	0.025425
fadJ	3-hydrox	6.0351	0.000134
fadR	DNA-bind	2.64872	0.003089
feaB	phenylac	2.35576	0.012609
fepE	polysacc	3.03501	0.002756
fes	enteroch	4.04475	0.000706
fhlA	DNA-bind	4.50111	0.000531
fimB	regulato	2.83876	0.004994
fimD	type I f	3.8474	0.00061
fimE	regulato	1.8954	0.035159
fimI	putative	5.65467	0.000136
fimZ	putative	4.17746	0.000284
focB	putative	2.31746	0.005396
folM	dihydrom	2.44774	0.009524
frlR	putative	2.69399	0.002818
frmB	S-formyl	2.21635	0.010534

frwC	putative	3.54824	0.001163
ftsW	putative	4.41123	0.000223
fucO	L-1,2-pr	2.93849	0.002153
fumA	fumarase	3.60624	0.001886
fumD	fumarase	3.07769	0.001758
gadC	L-glutam	3.59373	0.000506
gadY		3.13996	0.00195
galF	UTP:gluc	2.908	0.003627
gapC	EcoGene:	2.77467	0.003874
garP	galactar	3.11508	0.002264
gcl	glyoxyla	2.17704	0.013289
gfcB	lipoprot	3.15644	0.001686
gfcD	putative	3.11352	0.001843
glcA	glycolat	3.50144	0.001344
glcB	malate s	2.09708	0.016584
gloA	glyoxala	4.14622	0.00044
gloB	hydroxya	5.43372	3.88E-05
glpC	anaerobi	4.11824	0.000869
glpT	sn-glyce	2.47999	0.006683
glsB	glutamin	2.36569	0.004875
glyA	serine h	3.80796	0.000444
gnsA	putative	2.63495	0.004356
gsk	inosine/	4.80809	0.00054
gspC	Type II	1.85125	0.020354
gspL	Type II	3.06048	0.001622
gudD	D-glucar	3.19298	0.002558
gutM	DNA-bind	4.37677	0.000505
hcr	NADH oxi	3.48981	0.001403
hdeD	acid-res	1.78394	0.025241
hdhA	7-alpha-	2.24626	0.00514
hha	hemolysi	2.53505	0.005315
hinT	purine n	3.69142	0.000866
hipA	serine/t	1.83327	0.02744
hisG	ATP phos	1.74319	0.036255
holE	DNA poly	2.39872	0.009799
hprS	sensory	2.61356	0.004957
hscC	chaperon	3.99411	0.000319
hsdR	type I r	3.51326	0.001076
hupB	DNA-bind	1.95964	0.014522
hyaA	hydrogen	4.54402	0.000336
hyfD	hydrogen	3.27905	0.001268
hyi	hydroxyp	2.97323	0.002878
idnK	D-glucon	4.8864	0.000246
idnO	5-keto-D	4.22093	0.000407
inaA	putative	1.7063	0.037453

insD-3	CP4-44 p	3.55582	0.000755
insG	KpLE2 ph	3.27568	0.001066
insJ	insertio	3.19649	0.00186
insQ	putative	3.14375	0.001792
intD	DLP12 pr	2.22782	0.012958
intQ	EcoGene:	2.29266	0.008038
ivy	periplas	2.39657	0.009359
katE	catalase	3.33436	0.002529
kduD	putative	2.13233	0.019715
kefC	K(+) : H	3.69459	0.000955
kgtP	alpha-ke	2.16689	0.011347
kptA	RNA 2'-p	2.80717	0.001462
kup	K(+):H(+	3.40542	0.00224
lacA	galactos	3.99187	0.000867
lapB	lipopoly	2.25161	0.006516
ldcC	lysine d	2.72648	0.002275
leuE	leucine	2.79497	0.005382
leuO	DNA-bind	3.36947	0.003686
leuX	EcoGene:	3.25435	0.001623
lgoD	L-galact	3.83636	0.000705
ligB	DNA liga	3.82787	0.000311
lplA	lipoate-	3.13771	0.001553
lptG	lipopoly	2.30252	0.010983
lpxT	Kdo2-lip	2.56442	0.003247
lsrB	Autoindu	3.5847	0.001427
lsrF	3-hydrox	2.97408	0.002566
lysO	L-lysine	4.00853	0.000696
maeA	malate d	3.28258	0.001035
malK	maltose	2.55352	0.005871
malT	DNA-bind	3.91482	0.000522
marA	DNA-bind	3.56076	0.001286
mazG	nucleosi	2.3202	0.00734
mcrB	5-methyl	2.56304	0.007444
mdfA	multidru	2.39343	0.007827
mdtF	multidru	2.82678	0.003061
mdtI	multidru	3.45867	0.0012
melB	melibios	3.09716	0.002141
mepH	peptidog	3.85695	0.000523
mepS	peptidog	3.20293	0.000684
metF	5,10-met	2.88345	0.002622
mglB	D-galact	3.88476	0.000576
mglC	D-galact	3.79361	0.000465
mgtA	Mg(2(+))	4.6814	0.000306
mhpT	3-hydrox	2.12408	0.018052
mioC	flavopro	3.15141	0.000741

mlc	DNA-bind	3.19718	0.002274
mnmA	tRNA-spe	2.03552	0.014036
mntR	DNA-bind	3.72348	0.000632
mocA	molybden	2.18139	0.011053
mqsA	antitoxi	1.94406	0.03866
mrr	methylat	2.5297	0.009857
murB	UDP-N-ac	5.23451	0.000113
murJ	putative	4.33023	0.000451
mutH	DNA mism	2.81254	0.003705
mutS	DNA mism	2.551	0.004364
nadE	NAD synt	2.99299	0.001341
nagE	N-acetyl	2.4456	0.00484
nanS	N-acetyl	2.23442	0.016123
narU	nitrate/	3.51269	0.000744
narV	nitrate	2.29826	0.013573
nei	endonucl	2.20728	0.008705
nfsB	NAD(P)H	3.98317	0.000391
nhoA	arylamin	2.24048	0.008005
nimR	DNA-bind	4.12272	0.000548
nlpE	lipoprot	3.3203	0.000826
nrfA	cytochro	3.35355	0.001978
nth	endonucl	4.00807	0.000456
nudC	NADH pyr	2.17209	0.014075
nudI	pyrimidi	2.50241	0.004864
nupG	nucleosi	3.52195	0.001802
ompL	putative	3.05946	0.00223
ompW	outer me	3.35143	0.000801
omrA		3.28303	0.000849
opgG	osmoregu	3.03265	0.003698
oppA	oligopep	2.17627	0.008743
oppB	murein t	2.66589	0.006473
oppF	murein t	3.2729	0.001644
osmY	periplas	2.08277	0.013098
otsA	trehalos	2.34588	0.009072
otsB	trehalos	3.82569	0.000456
pagP	Lipid IV	2.63611	0.003879
patD	gamma-am	2.51373	0.006787
pdeA	putative	3.1388	0.002298
pdeC	c-di-GMP	2.78455	0.002362
pdeG	putative	3.22888	0.001687
pdeI	putative	4.13584	0.0005
pdxI	pyridoxi	2.64872	0.003089
pfkB	6-phosph	5.95236	4.29E-05
pgaA	partiall	2.64082	0.003284
pgaC	poly-N-a	3.31088	0.001266

pgaD	poly-N-a	3.28279	0.00094
pgpB	phosphat	4.4898	0.000471
pgrR	DNA-bind	2.72683	0.003104
phoE	outer me	2.77467	0.003874
phoR	sensory	2.87587	0.003837
phr	deoxyrib	2.6502	0.004361
plaP	putresci	2.9611	0.001127
plsY	putative	5.74664	0.000123
pnuC	nicotina	3.75346	0.000834
ppiB	peptidyl	2.43207	0.009286
pqiA	intermem	2.87167	0.004811
pqqL	putative	2.78079	0.003428
preA	NAD-depe	2.01713	0.019529
prkB	putative	2.28181	0.010895
prlF	antitoxi	3.58466	0.001249
proP	osmolyte	2.86009	0.004358
proY	putative	4.11058	0.00053
psd	phosphat	6.61404	0.000175
pspA	phage sh	3.23177	0.001068
pspE	thiosulf	2.4833	0.005857
psuG	pseudour	3.48637	0.001888
psuT	putative	2.70779	0.005987
purF	amidopho	3.90306	0.000833
purH	bifuncti	4.61519	0.000222
pyrF	orotidin	1.98051	0.024167
qseC	sensory	5.24953	0.000126
queD	6-carbox	3.91795	0.00044
ralA	EcoGene:	2.87159	0.00321
rapZ	RNase ad	3.4077	0.001156
rbn	ribonucl	6.15999	9.97E-05
rcdA	DNA-bind	3.62215	0.000653
rclA	putative	2.22594	0.010566
rclR	DNA-bind	2.24198	0.008997
rcsC	sensory	2.12031	0.017296
rhmD	L-rhamno	4.96733	0.00023
rhsA	rhs elem	2.67375	0.003906
rhsB	rhs elem	3.26683	0.0006
rhsC	rhs elem	4.34693	0.000534
rhsD	protein	4.91196	0.0002
rhsE	EcoGene:	2.91494	0.002578
rimK	ribosoma	3.07653	0.00191
rimL	ribosoma	2.88055	0.002023
rlhA	23S rRNA	2.7037	0.008025
rlmB	23S rRNA	3.81362	0.000814
rng	RNase G	2.97008	0.002521

rpiB	allose-6	3.81836	0.000766
rpnB	recombin	2.87497	0.003594
rpnC	recombin	2.2148	0.018134
rpnD	EcoGene:	2.14164	0.013103
rpsJ	30S ribo	4.18869	0.000746
rraB	ribonucl	3.56034	0.001452
rspB	putative	3.0562	0.001467
rssA	putative	6.15757	4.83E-05
rssB	regulato	1.95462	0.014175
rtcB	RNA-spli	4.74327	0.000225
rybA		6.45678	0.000155
sapD	putresci	2.95145	0.004519
sbmA	peptide	2.15825	0.015261
scpB	methylma	1.84282	0.037437
sdhC	succinat	2.01387	0.015101
setC	putative	2.50538	0.007359
sfmC	putative	4.12312	0.000194
sfmD	putative	3.19718	0.002274
sfsB	putative	3.2539	0.001033
shiA	shikimat	2.08937	0.011204
sieB	Rac prop	5.03026	0.00035
slp	starvati	3.38241	0.00158
smf	protein	3.50439	0.001094
sodA	superoxi	2.09705	0.007582
sohB	S49 pept	2.86765	0.00199
soxR	DNA-bind	2.01594	0.01786
speF	ornithin	2.42642	0.006401
speG	spermidi	3.01657	0.000864
srlA	sorbitol	3.063	0.001748
sseA	3-mercap	2.3313	0.005644
tag	3-methyl	3.64747	0.001033
talA	transald	2.6987	0.003712
tamA	transloc	2.04756	0.013563
tar	methyl-a	3.24284	0.001068
tdcE	2-ketobu	3.09253	0.001475
tdcR	DNA-bind	2.79554	0.004574
tehA	tellurit	3.83597	0.000199
tgt	tRNA-gua	4.29385	0.00063
thiI	tRNA uri	3.20155	0.001464
tktB	transket	2.43768	0.007453
tnaA	tryptoph	3.05191	0.002287
tonB	Ton comp	2.59089	0.008793
torT	periplas	2.76964	0.002405
treA	periplas	2.57815	0.005322
treF	cytoplas	2.66149	0.005414

trkG	Rac prop	2.77487	0.003659
trmA	tRNA m(5	3.44856	0.000879
tsgA	putative	3.43966	0.001517
tsr	methyl-a	4.01993	0.000456
ttcC	EcoGene:	2.3581	0.007955
tynA	copper-c	3.13056	0.00081
tyrB	tyrosine	2.5615	0.015441
tyrP	tyrosine	4.62469	0.000249
uacT	urate:H(	3.78689	0.000848
ubiG	bifuncti	3.00905	0.001453
ubiK	ubiquino	2.56497	0.004441
ucpA	putative	3.58325	0.000992
ugd	UDP-gluc	2.74641	0.003862
ugpB	sn-glyce	3.33416	0.001397
uidA	beta-glu	2.66597	0.003238
uidC	outer me	2.44501	0.00846
ulaR	DNA-bind	3.04758	0.001662
umuC	DNA poly	2.98827	0.001183
ung	uracil-D	2.89322	0.00361
upp	uracil p	3.33983	0.000712
ushA	5'-nucle	3.40939	0.00088
uvrB	excision	2.14442	0.015261
ves	HutD fam	2.77175	0.002219
wcaA	putative	3.69764	0.00103
wcaE	putative	3.76881	0.000814
wecH	O-acetyl	2.08117	0.019825
wza	outer me	3.13543	0.001316
wzzB	regulato	2.17309	0.013769
xanQ	xanthine	3.41631	0.000939
xdhC	putative	2.18533	0.007547
xseA	exodeoxy	2.75795	0.004798
xthA	exodeoxy	3.78206	0.000989
xylF	xylose A	3.86907	0.000859
xylH	xylose A	2.62649	0.005546
xylR	DNA-bind	2.24278	0.009004
yaaU	putative	6.97264	0.000186
yadG	putative	2.49027	0.006436
yadH	putative	6.95438	3.85E-05
yadM	putative	2.45536	0.006509
yadV	putative	3.55219	0.001078
yafN	antitoxi	2.46402	0.002794
yafT	lipoprot	4.44276	0.000407
yagU	inner me	3.94266	0.000475
yahD	ankyrin	7.41019	1.71E-05
yahJ	putative	2.5358	0.004461

yahO	DUF1471	2.36403	0.01313
yaiZ	DUF2754	3.92317	0.000276
ybaL	putative	9.59544	2.58E-05
ybaQ	putative	3.47356	0.001005
ybaV	helix-ha	3.5731	0.001019
ybbP	putative	2.66867	0.005122
ybbW	putative	4.76308	0.000132
ybcL	DLP12 pr	3.51187	0.000971
ybcW	DLP12 pr	2.03155	0.019642
ybdG	minicond	3.53188	0.001094
ybdK	carboxyl	2.85198	0.004026
ybdR	putative	2.21635	0.010534
ybeD	DUF493 d	2.00079	0.009804
ybeF	putative	3.17329	0.001867
ybeL	DUF1451	1.88971	0.039902
ybfL	EcoGene:	2.47781	0.005246
ybfO	EcoGene:	2.13937	0.010612
ybfP	lipoprot	4.17871	0.0004
ybjI	5-amino-	2.90529	0.002032
ybjM	putative	3.21655	0.000962
ycaK	putative	2.63074	0.004056
ycbU	putative	2.14057	0.009631
yccU	putative	3.02474	0.001739
ycdX	zinc-bin	2.58304	0.002878
ycdZ	putative	3.17246	0.002004
yceJ	putative	2.92188	0.001761
yceK	DUF1375	3.3895	0.001293
yceQ	DUF2655	4.78352	0.00021
ycfT	inner me	6.07512	0.000684
ycfZ	putative	3.8256	0.000827
ycgH	EcoGene:	2.81869	0.005253
ycgI	EcoGene:	2.43278	0.003602
ycgJ	PF05666	2.9801	0.000887
ycgL	PF05166	3.85364	0.000895
ycgN	PF03693	2.19793	0.008213
ycgV	putative	2.76087	0.003861
ycgX	uncharac	4.7812	0.000154
ycgZ	putative	2.45506	0.006649
yciQ	DUF2207	2.24838	0.012458
yciW	putative	3.06492	0.001831
ycjF	conserve	2.6452	0.002911
ycjG	L-Ala-D/	3.2317	0.00155
ycjM	glucosyl	3.72927	0.000668
ycjV	EcoGene:	2.11131	0.010623
ydbD	DUF2773	2.5218	0.006016

ydbH	PF11739	3.29383	0.001801
ydcR	fused pu	3.39211	0.001245
yddW	putative	3.27001	0.001567
ydeA	L-arabin	3.54505	0.000839
ydeE	dipeptid	2.19793	0.008213
ydeO	DNA-bind	2.72187	0.005115
ydeP	putative	2.31244	0.016087
ydeQ	putative	2.434	0.002594
ydfE	EcoGene:	4.76856	0.000487
ydfG	3-hydrox	3.17795	0.001451
ydfI	putative	2.68228	0.002523
ydgA	conserve	2.61903	0.001949
ydhK	putative	2.77487	0.003659
ydhP	putative	3.15226	0.0018
ydhQ	adhesin-	4.04564	0.001065
ydhR	putative	2.92637	0.002012
ydhV	putative	2.79472	0.00312
ydiF	putative	3.36923	0.001141
ydiK	putative	2.16654	0.012166
ydiM	putative	3.09896	0.00131
ydiP	putative	1.98064	0.018528
ydiV	anti-Flh	3.6627	0.00053
ydjE	putative	2.60445	0.004131
ydjF	putative	2.58201	0.004598
ydjG	NADH-dep	2.81792	0.002884
ydjI	putative	2.1403	0.014676
ydjJ	putative	3.54018	0.001039
ydjL	putative	2.81792	0.002884
ydjY	4Fe-4S f	4.18181	0.000371
yeaH	DUF444 d	3.62643	0.001193
yeaV	putative	6.64458	6.52E-05
yebV	protein	4.56427	0.000425
yeeA	putative	2.77485	0.002635
yeeD	putative	2.79844	0.004268
yeeJ	inverse	2.64797	0.003818
yeeN	putative	3.21816	0.001443
yeeX	DUF496 d	2.03919	0.010223
yegH	inner me	3.61657	0.000589
yegS	lipid ki	2.71126	0.004454
yehR	DUF1307	3.68315	0.00065
yehS	conserve	2.08162	0.015914
yeiG	S-formyl	3.60651	0.000386
yeiL	putative	3.55019	0.001102
yeiS	DUF2542	2.90474	0.001877
yejA	putative	6.1914	8.01E-05

yejE	putative	3.32037	0.002543
yejF	putative	3.27427	0.000998
yejM	putative	3.54813	0.000272
yfaA	DUF2138	5.31562	9.43E-05
yfaL	putative	3.37515	0.001288
yfaQ	tandem D	4.16266	0.000562
yfaZ	putative	2.75624	0.003522
yfcV	putative	2.50062	0.00351
yfdV	putative	4.23989	0.000488
yfdX	protein	2.64961	0.002402
yfeC	putative	5.6406	0.00011
yfeH	putative	3.35451	0.001517
yfeS	conserve	2.04393	0.017766
yfgG	protein	2.85672	0.001435
yfiE	putative	4.05255	0.000368
ygbE	conserve	4.51869	0.000413
ygcE	putative	3.01677	0.002447
ygdG	flap end	2.99763	0.001815
ygdQ	UPF0053	3.45008	0.000532
ygeR	LysM dom	2.92859	0.005268
ygeV	putative	3.80939	0.000745
ygeW	putative	3.55642	0.001877
ygfI	putative	3.32481	0.001343
ygfK	putative	2.32429	0.013677
ygfZ	folate-b	2.63917	0.007427
yggM	DUF1202	3.33072	0.001801
yggP	putative	4.89741	0.000152
yggT	uncharac	3.07368	0.003569
yghG	lipoprot	4.25311	0.000161
yghO	putative	2.47519	0.007884
yghQ	putative	1.88233	0.011884
ygiC	putative	2.41613	0.008853
ygjI	putative	3.39297	0.001651
ygjP	putative	2.29951	0.005966
yhaH	putative	2.50771	0.006408
yhcB	conserve	2.45846	0.004127
yhdJ	DNA aden	3.21335	0.001732
yhdU	DUF2556	3.14483	0.00195
yhdZ	putative	2.90849	0.005984
yhfL	DUF4223	3.34734	0.001003
yhfW	putative	2.68064	0.004364
yhgH	DNA util	4.30313	0.000192
yhhT	putative	2.31043	0.009637
yhhX	putative	3.03606	0.001768
yhhY	N-acetyl	2.82632	0.001954

yhiJ	DUF4049	2.48093	0.006391
yhjD	putative	2.47451	0.006189
yhjG	AsmA fam	3.42274	0.000756
yhjV	putative	6.70431	9.67E-05
yiaK	2,3-dike	3.36778	0.001966
yiaN	2,3-dike	3.12656	0.001006
yiaY	L-threon	3.11928	0.00171
yibJ	EcoGene:	2.5425	0.008925
yicH	AsmA fam	2.47472	0.008081
yicJ	putative	3.56727	0.00128
yicN	conserve	2.50241	0.004864
yicS	uncharac	4.48239	0.000301
yidE	putative	2.97367	0.002715
yidI	putative	3.49878	0.00094
yidL	putative	3.03331	0.004586
yidP	putative	4.16598	0.001063
yidX	putative	4.02963	0.000407
yidZ	putative	2.63835	0.004259
yifN	EcoGene:	2.91433	0.003186
yigE	DUF2233	2.67273	0.004997
yihN	putative	2.9491	0.002776
yihR	putative	2.62312	0.004706
yihW	putative	3.49843	0.00101
yiiE	putative	3.59039	0.00093
yiiG	DUF3829	2.58217	0.001683
yiiQ	DUF1454	2.90208	0.001397
yiiR	DUF805 d	3.38256	0.000756
yijE	cystine	4.18359	0.000544
yijF	conserve	4.38107	0.00016
yjaG	conserve	2.81668	0.001741
yjaZ	conserve	3.36615	0.002075
yjbD	conserve	2.3954	0.005977
yjbH	YjbH fam	5.8791	0.000198
yjbI	EcoGene:	2.77294	0.003386
yjbJ	putative	3.77683	0.000455
yjdI	PF06902	4.57505	0.000251
yjdM	conserve	2.9752	0.002186
yjeM	putative	4.5984	0.000398
yjeV	uncharac	2.71122	0.00456
yjgN	conserve	3.81835	0.000664
yjgX	EcoGene:	3.031	0.002335
yjhB	putative	2.79463	0.003428
yjhR	EcoGene:	4.12021	0.000662
yjhZ	EcoGene:	2.9207	0.000265
yjiK	uncharac	4.10718	0.001232

yjiM	putative		2.86133	0.001562
yjiV	EcoGene:		3.0817	0.002621
yjjB	putative		3.4033	0.000589
yjjQ	DNA-bind		4.38273	0.000417
ykgA	EcoGene:		3.49504	0.000461
ykgE	putative		2.50241	0.004864
ykgG		#N/A	2.8606	0.002597
ykgL	uncharac		4.09417	0.00045
ykgP	EcoGene:		2.5309	0.003151
ylaC	putative		2.75113	0.002758
ylcG	DLP12 pr		2.6828	0.004012
yliI	aldose s		3.2525	0.001681
ymdG	protein		3.94288	0.000384
ymgE	PF04226		2.88381	0.001886
ymgM	protein		3.24767	0.000932
ymiA	uncharac		3.01941	0.001337
ymjC	putative		5.07462	0.000298
ynbC	hydrolas		2.76299	0.002635
yncE	PQQ-like		2.28666	0.022358
yncG	putative		3.6268	0.000824
yneK	protein		4.67481	0.00022
yneO	EcoGene:		3.30369	0.001922
yneP	protein		2.7332	0.002923
ynfA	conserve		3.62576	0.000505
ynfE	putative		2.35721	0.005207
ynfF	putative		3.24001	0.001821
yniA	putative		3.21683	0.00211
ynjI	DUF1266		3.12589	0.002614
yohJ	PF03788		2.20389	0.010534
ypdI	colanic		2.26495	0.011241
ypfN	UPF0370		2.45846	0.004127
yphF	putative		5.35076	0.000163
yqcE	putative		2.40951	0.007147
yqcG	cell env		4.81087	0.00017
yqeA	putative		2.7201	0.00193
yqeG	putative		4.01743	0.000912
yqeH		#N/A	2.41641	0.003586
yqhA	uncharac		2.84179	0.001619
yqiH	putative		2.12153	0.019122
yraI	putative		2.35815	0.014839
yraJ	putative		3.39808	0.001326
yraK	putative		2.59243	0.004279
yrdA	protein		3.56932	0.000588
yrdD	putative		3.94701	0.000545
yrhD	uncharac		12.03588	6.44E-06

ysdD	protein	5.08069	0.000185
zapC	cell div	2.18883	0.012116
zinT	metal-bi	2.14845	0.008027
zitB	Zn(2(+))	4.24332	0.000253
zntB	Zn(2(+))	5.69192	0.000158
aaeR	LysR-typ	3.15807	0.00127
acnA	aconitat	2.37116	0.005175
acrF	multidru	5.47303	0.000176
aidB	putative	6.13982	7.4E-05
arnT	lipid IV	3.72316	0.001272
atoC	DNA-bind	2.32875	0.008729
dgcE	putative	3.81112	0.000476
dosP	oxygen-s	7.02668	4.24E-05
katE	catalase	3.07545	0.00168
ompW	outer me	2.96956	0.00347
pdeI	putative	4.11056	0.000615
pdeI	putative	3.36731	0.00158
pqqL	putative	6.89165	0.001264
rbn	ribonucl	2.55844	0.000926
sfmD	putative	3.31242	0.0014
sohB	S49 pept	2.66602	0.002279
tehA	tellurit	2.3202	0.00734
tktB	transket	2.34083	0.004759
treF	cytoplas	3.28943	0.001072
tsgA	putative	4.15513	0.00056
ybdR	putative	5.01687	0.00019
ycgV	putative	2.24195	0.010447
ydeE	dipeptid	4.10551	0.000536
yeaV	putative	2.11507	0.002093
yeeJ	inverse	2.50304	0.005352
ygeR	LysM dom	3.13815	0.001332
yjhR	EcoGene:	2.85879	0.001632
zntB	Zn(2(+))	4.37755	0.000382

Supplementary Table 7. Bacterial strains used in this study

Strain	Relevant characteristic(s)	Reference or source
B. coagulans 2-6	Wild type	ref. 22
E. coli DH5α	*supE44ΔlacU169* (*Φ80 lacZΔM15*) *hsdR17 recA1 endA1 gyrA96 thi-1 relA1*	Novagen
E. coli BL21 (DE3)	*F- ompT hsdSB (rB-mB-) gal (λ c I 857 ind1 Sam7 nin5 lacUV5 T7gene1) dcm (DE3)*	Novagen
P. putida KT2440	*rmo- mod+*	ATCC
S. cerevisiae INVSc1	*MATa his3D1 leu2 trp1-289 ura3-52 MAT his3D1 leu2 trp1-289 ura3-52*	ATCC
ATCC31280::pLQ856	wild-type harboring pLQ856	Ning, Wang *et al*., 2017
ATCC31280::pLQ856-cspL	wild-type harboring pLQ856-*cspL*	This study
DH5α	*E. coli* DH5α harboring pUC19 empty vector	This study
DH5α-*cspL*	*E. coli* DH5α harboring pUC19-*cspL*	This study
DH5α-*cspD*	*E. coli* DH5α harboring pUC19-*cspD*	This study
DH5α-*BCO26_YkuS*	*E. coli* DH5α harboring pUC19-*BCO26_ykuS*	This study
DH5α-*BCO26_2915*	*E. coli* DH5α harboring pUC19*BCO26_2915*	This study
DH5α-*BCO26_Dps*	*E. coli* DH5α harboring pUC19*BCO26_dps*	This study
DH5α-*BCO26_GsiB*	*E. coli* DH5α harboring pUC19*BCO26_gsiB*	This study
DH5α-*BCO26_YbfB*	*E. coli* DH5α harboring pUC19*BCO26_ybfB*	This study
DH5α-*BCO26_MntH*	*E. coli* DH5α harboring pUC19*BCO26_mntH*	This study
DH5α-*BCO26_2932*	*E. coli* DH5α harboring pUC19*BCO26_2932*	This study
DH5α-*BCO26_GabD*	*E. coli* DH5α harboring	This study

	pUC19*BCO26_gabD*	
DH5α-*BCO26_GroEL*	*E. coli* DH5α harboring pUC19*BCO26_groEL*	This study
DH5α-*BCO26_HrcA*	*E. coli* DH5α harboring pUC19*BCO26_hrcA*	This study
DH5α-*BCO26_ClpE*	*E. coli* DH5α harboring pUC19*BCO26_clpE*	This study
DH5α-*BCO26_1771*	*E. coli* DH5α harboring pUC19*BCO26_1771*	This study
DH5α-*BCO26_DnaK*	*E. coli* DH5α harboring pUC19*BCO26_dnaK*	This study
DH5α-*BCO26_GroES*	*E. coli* DH5α harboring pUC19*BCO26_groES*	This study
DH5α-*BCO26_0541*	*E. coli* DH5α harboring pUC19*BCO26_0541*	This study
DH5α-*BCO26_YkzI*	*E. coli* DH5α harboring pUC19*BCO26_ykzI*	This study
DH5α-*BCO26_KatE*	*E. coli* DH5α harboring pUC19*BCO26_katE*	This study
DH5α-*BCO26_0399*	*E. coli* DH5α harboring pUC19*BCO26_0399*	This study
DH5α-*BCO26_2461*	*E. coli* DH5α harboring pUC19*BCO26_2461*	This study
DH5α-*BCO26_LevG*	*E. coli* DH5α harboring pUC19*BCO26_levG*	This study
DH5α-*BCO26_YqiG*	*E. coli* DH5α harboring pUC19*BCO26_yqiG*	This study
DH5α-*BCO26_2340*	*E. coli* DH5α harboring pUC19*BCO26_2340*	This study
DH5α-*BCO26_YflT*	*E. coli* DH5α harboring pUC19*BCO26_yflT*	This study
DH5α-*BCO26_ArgI*	*E. coli* DH5α harboring pUC19*BCO26_argL*	This study
DH5α-*BCO26_SucC*	*E. coli* DH5α harboring pUC19*BCO26_sucC*	This study
DH5α-*BCO26_2370*	*E. coli* DH5α harboring pUC19*BCO26_2370*	This study
DH5α-*BCO26_CitZ*	*E. coli* DH5α harboring pUC19*BCO26_citZ*	This study
DH5α-*BCO26_RsbV*	*E. coli* DH5α harboring	This study

	pUC19*BCO26_rsbV*	
DH5α-*BCO26_Hag*	*E. coli* DH5α harboring pUC19*BCO26_rhag*	This study
DH5α-*BCO26_YhgD*	*E. coli* DH5α harboring pUC19*BCO26_yhgD*	This study
DH5α-*BCO26_1679*	*E. coli* DH5α harboring pUC19*BCO26_1679*	This study
DH5α-*BCO26_RsbW*	*E. coli* DH5α harboring pUC19*BCO26_rsbW*	This study
DH5α-*BCO26_YteA*	*E. coli* DH5α harboring pUC19*BCO26_yteA*	This study
DH5α-*BCO26_2825*	*E. coli* DH5α harboring pUC19*BCO26_2825*	This study
DH5α-*BCO26_LevE*	*E. coli* DH5α harboring pUC19*BCO26_levE*	This study
DH5α-*BCO26_SigB*	*E. coli* DH5α harboring pUC19*BCO26_sigB*	This study
DH5α-*BCO26_2573*	*E. coli* DH5α harboring pUC19*BCO26_2573*	This study
DH5α-*BCO26_1484*	*E. coli* DH5α harboring pUC19*BCO26_1484*	This study
BL21-*cspL*	*E. coli* BL21(DE3) harboring pET28a-*cspL*	This study
BL21-*cspL*-M11	*E. coli* BL21(DE3) harboring pET28a-*cspL* with mutated G14 Y15 G16 F17 I18 E19 R20 V26 F27 V28 H29	This study
BL21-*cspL*-M7	*E. coli* BL21(DE3) harboring pET28a-*cspL* with mutated G14 Y15 G16 F17 I18 E19 R20	This study
INVSc1	*S. cerevisiae* INVSc1 harboring pYES2 empty vector	This study
INVSc1-*cspL*	*S. cerevisiae* INVSc1 harboring pYES2-*cspL*	This study
KT2440	*P. putida* KT2440 harboring pME6032 empty vector	This study
KT2440-*cspL*	*P. putida* KT2440 harboring pME6032-*cspL*	This study
DH5α-*cspA*	*E. coli* DH5α harboring pUC19-*cspA*	This study

Supplementary Table 8. Plasmids used in this study

Plasmid	Relevant characteristic(s)[a]	Reference or source
pUC19	Amp^R, *pMB1 ori*, P*lacZ*	Novagen
pET28a(+)	Kan^R, *pBR322 ori*, PT7	Novagen
pUC19-*cspL*	pUC19 harboring *cspL* from *B. coagulans 2-6*	This study
pUC19-*BCO26_cspD*	pUC19 harboring *BCO26_cspD* from *B. coagulans 2-6*	This study
pUC19-*BCO26_ykuS*	pUC19 harboring *BCO26_ykuS* from *B. coagulans 2-6*	This study
pUC19-*BCO26_2915*	pUC19 harboring *BCO26_2915* from *B. coagulans 2-6*	This study
pUC19-*BCO26_dps*	pUC19 harboring *BCO26_dps* from *B. coagulans 2-6*	This study
pUC19-*BCO26_gsiB*	pUC19 harboring *BCO26_gsiB* from *B. coagulans 2-6*	This study
pUC19-*BCO26_ybfB*	pUC19 harboring *BCO26_ybfB* from *B. coagulans 2-6*	This study
pUC19-*BCO26_mntH*	pUC19 harboring *BCO26_mntH* from *B. coagulans 2-6*	This study
pUC19-*BCO26_2932*	pUC19 harboring *BCO26_2932* from *B. coagulans 2-6*	This study
pUC19-*BCO2_gabD*	pUC19 harboring *BCO26_gabD* from *B. coagulans 2-6*	This study
pUC19-*BCO2_groEL*	pUC19 harboring *BCO26_groEL* from *B. coagulans 2-6*	This study
pUC19-*BCO26_hrcA*	pUC19 harboring *BCO26_hrcA* from *B. coagulans 2-6*	This study
pUC19-*BCO26_clpE*	pUC19 harboring *BCO26_clpE* from *B. coagulans 2-6*	This study
pUC19-*BCO26_1771*	pUC19 harboring *BCO26_1771* from *B. coagulans 2-6*	This study
pUC19-*BCO26_dnaK*	pUC19 harboring *BCO26_dnaK* from *B. coagulans 2-6*	This study
pUC19-*BCO26_groES*	pUC19 harboring *BCO26_groES* from	This study

	B. coagulans 2-6	
pUC19-*BCO26_0541*	pUC19 harboring *BCO26_0541* from *B. coagulans 2-6*	This study
pUC19-*BCO26_ykzI*	pUC19 harboring *BCO26_ykzI* from *B. coagulans 2-6*	This study
pUC19-*BCO26_katE*	pUC19 harboring *BCO26_katE* from *B. coagulans 2-6*	This study
pUC19-*BCO26_0399*	pUC19 harboring *BCO26_0399* from *B. coagulans 2-6*	This study
pUC19-*BCO26_2461*	pUC19 harboring *BCO26_2461* from *B. coagulans 2-6*	This study
pUC19-*BCO26_levG*	pUC19 harboring *BCO26_levG* from *B. coagulans 2-6*	This study
pUC19-*BCO26_yqiG*	pUC19 harboring *BCO26_yqiG* from *B. coagulans 2-6*	This study
pUC19-*BCO26_2340*	pUC19 harboring *BCO26_2340* from *B. coagulans 2-6*	This study
pUC19-*BCO26_yflT*	pUC19 harboring *BCO26_yflT* from *B. coagulans 2-6*	This study
pUC19-*BCO26_argI*	pUC19 harboring *BCO26_argI* from *B. coagulans 2-6*	This study
pUC19-*BCO26_sucC*	pUC19 harboring *BCO26_sucC* from *B. coagulans 2-6*	This study
pUC19-*BCO26_2370*	pUC19 harboring *BCO26_2370* from *B. coagulans 2-6*	This study
pUC19-*BCO26_citZ*	pUC19 harboring *BCO26_citZ* from *B. coagulans 2-6*	This study
pUC19-*BCO26_rsbV*	pUC19 harboring *BCO26_rsbV* from *B. coagulans 2-6*	This study
pUC19-*BCO26_hag*	pUC19 harboring *BCO26_hag* from *B. coagulans 2-6*	This study
pUC19-*BCO26_yhgD*	pUC19 harboring *BCO26_yhgD* from *B. coagulans 2-6*	This study
pUC19-*BCO26_1679*	pUC19 harboring *BCO26_1679* from *B. coagulans 2-6*	This study
pUC19-*BCO26_rsbW*	pUC19 harboring *BCO26_rsbW* from *B. coagulans 2-6*	This study
pUC19-*BCO26_yteA*	pUC19 harboring *BCO26_yteA* from *B. coagulans 2-6*	This study
pUC19-*BCO26_2825*	pUC19 harboring *BCO26_2825* from	This study

	B. coagulans 2-6	
pUC19-*BCO26_levE*	pUC19 harboring *BCO26_levE* from *B. coagulans 2-6*	This study
pUC19-*BCO26_sigB*	pUC19 harboring *BCO26_sigB* from *B. coagulans 2-6*	This study
pUC19-*BCO26_2573*	pUC19 harboring *BCO26_2573* from *B. coagulans 2-6*	This study
pUC19-*BCO26_1484*	pUC19 harboring *BCO26_1484* from *B. coagulans 2-6*	This study
pET28a-*cspL*	pET28a harboring *BCO26_cspL* from *B. coagulans* 2-6	This study
pET28a-*cspL*-M11	pET-28a harboring *BCO26_cspL* from *B. coagulans 2-6* with mutated G14 Y15 G16 F17 I18 E19 R20 V26 F27 V28 H29	This study
pET28a-*cspL*-M7	pET-28a harboring *BCO26_cspL* from *B. coagulans 2-6* with mutated G14 Y15 G16 F17 I18 E19 R20	This study
pYES2-*cspL*	pYES2 harboring *BCO26_cspL* from *B. coagulans 2-6*	This study
pME6032-*cspL*	pME6032 harboring *BCO26_cspL* from *B. coagulans 2-6*	Novagen
pUC19-*cspA*	pUC19 harboring *cspA* from *E. coli*	This study
pLQ856	pDR3 derivative	Ning, Wang *et al.*, 2017
pLQ856-*cspL*	pLQ856 derivative with inserted *cspL* under the control of kasOp*	This study

[a]Amp^R and Kan^R resistance to ampicillin and kanamycin, respectively.

Supplementary Table 9. Sequences of primers used in this study

Primer	Sequence (5' > 3')
CspL-F	CGCGGATCC atggaacatggtacagtaaa
CspL-R	CCGGAATTC ttagtcttctttttgaacat
BCO26_CspD-F	CGCGGATCC atgcaaaacggtaaagtaaa
BCO26_CspD-R	CCGGAATTC ttatgaaagttttgttacat
BCO26_YkuS-F	CGCGGATCC atggctgtaatcggtgtaga
BCO26_YkuS-R	CCGGAATTC ttacattctgctcgccactt
BCO26_2925-F	CCCAAGCTT atgaaaaagcgggcaattgt
BCO26_2925-R	CGCGGATCC tcaggaaaccactgcctttt
BCO26_Dps-F	CGCGGATCC atggcagaaaacgaacaatt
BCO26_Dps-R	CCGGAATTC ttaccgctttccaagaaagg
BCO26_GsiB-F	CCCAAGCTT atggcagacaaagataaaaa
BCO26_GsiB-R	CGCGGATCC ttaatcttcaccgttgtttt
BCO26_YbfB-F	CGCGGATCC atgttagaacgaaaagcaaa
BCO26_YbfB-R	CCGGAATTC ttaatgcgaatgctgggcac
BCO26_MntH-F	CCCAAGCTT atgagtgaaaaaatgatgag
BCO26_MntH-R	CCGGAATTC ttatataaacgtatcaatca
BCO26_2932-F	CCCAAGCTT atgagcatcagacagggaaa
BCO26_2932-R	CGCGGATCC ttaccggtaggccggttctt
BCO26_GabD-F	CGCGGATCC atggaagactatttgatgta
BCO26_GabD-R	CCGGAATTC ttataaaccgagggaaatat
BCO26_GroEL-F	CGCGGATCC atggcaaaagaaattaaatt
BCO26_GroEL-R	CCGGAATTC ttacatcatgccgcccatgc
BCO26_HrcA-F	CGCGGATCC atggcggatcttgaggaact
BCO26_HrcA-R	CCGGAATTC ctatctgtcatacaatttcg
BCO26_ClpE-F	CGCGGATCC atgttatgtgacaaatgcca
BCO26_ClpE-R	CCGGAATTC ttatttttccgcgatggcaa

BCO26_1771-F	CCCAAGCTT atgtttgatttaatgccatt
BCO26_1771-R	CGCGGATCC ttattgaatttcaatccttt
BCO26_DnaK-F	CGCGGATCC atgagcaaaattatcggcat
BCO26_DnaK-R	CCGGAATTC ttattttttattatcatcga
BCO26_GroES-F	CGCGGATCC atgtttcacgtgttaaaacc
BCO26_GroES-R	CCGGAATTC ttattccacaaccgccagaa
BCO26_0541-F	CGCGGATCC atggtaggaattatcattgc
BCO26_0541-R	CCGGAATTC ttatttttgtttgttcagct
BCO26_YkzI-F	CGCGGATCC atgaaacaagtaatcccttc
BCO26_YkzI-R	CCGGAATTC ttacatggctttcattttca
BCO26_KatE-F	CTAGTCTAGA atgagtagtgaacggaaact
BCO26_KatE-R	CGAGCTC tcatatcaaacgcctgtccc
BCO26_0399-F	CCCAAGCTT atgccattggaactggtaat
BCO26_0399-R	CTAGTCTAGA tcaatgctttccccccctcta
BCO26_2461-F	CGCGGATCC atgatgaaaaaatcaatggc
BCO26_2461-R	CCGGAATTC tcactttttcgcgcctgcaa
BCO26_LevG-F	CCCAAGCTT atggcacaagaactaaaatt
BCO26_LevG-R	CTAGTCTAGA ttacattaagtgaattaaat
BCO26_YqiG-F	CGCGGATCC atgagcaaatacgataaact
BCO26_YqiG-R	CCGGAATTC tcactctgcaaacgggaacc
BCO26_2340-F	CCCAAGCTT atgaaccgaaatttttgtaa
BCO26_2340-R	CGCGGATCC ttatctttcggcactccgca
BCO26_YflT-F	CGCGGATCC atgcataaagtagaagtggt
BCO26_YflT-R	CCGGAATTC ttatagcaggtgttcaggcc
BCO26_ArgI-F	CGCGGATCC atggagaaacatattgcaat
BCO26_ArgI-R	CCGGAATTC ttaaagaagtttttcccga
BCO26_SucC-F	CGCGGATCC atgaatattcacgagtatca
BCO26_SucC-R	CCGGAATTC ttagctgaccagttcgacaa
BCO26_2370-F	CGCGGATCC atgagatcgattgtaaatga

BCO26_2370-R	CCGGAATTC ttaatacagcgtaatttcct
BCO26_CitZ-F	CGCGGATCC atgacagcaacaagaggtct
BCO26_CitZ-R	CCGGAATTC ttaccggtcttcgagcggaa
BCO26_RsbV-F	CGCGGATCC atggactttgaagtagatgt
BCO26_RsbV-R	CCGGAATTC tcacactccaccttctattt
BCO26_Hag-F	CCCAAGCTT atgattatcaatcacaacat
BCO26_Hag-R	CGCGGATCC ttaacgcaacaattgcaata
BCO26_YhgD-F	ACATGCATGC atggaaacggaccgcaggct
BCO26_YhgD-R	CTAGTCTAGA tcagttctgcagcccttttta
BCO26_1679-F	CTAGTCTAGA atgtctgttgcttcgacaga
BCO26_1679-R	TCCCCCGGG ttacaagccgcattttaatt
BCO26_RsbW-F	CGCGGATCC atggaggagtttgatcatat
BCO26_RsbW-R	CCGGAATTC tcaggttgaggcagttttga
BCO26_YteA-F	CCCAAGCTT atgctgacaaaagaacaact
BCO26_YteA-R	CGCGGATCC tcatttcttttttttctcgt
BCO26_2825-F	CGCGGATCC atgggggcgattcagatcat
BCO26_2825-R	CCGGAATTC tcatgcccggcccccccctt
BCO26_LevE-F	CGCGGATCC atggcattggatatacggct
BCO26_LevE-R	CCGGAATTC tcatggatgaagcagtttat
BCO26_SigB-F	CGCGGATCC atgtcaaaactgcctcaacc
BCO26_SigB-R	CCGGAATTC ctaattctccacatgttgca
BCO26_2573-F	ACATGCATGC atggaaaacattaaaatgct
BCO26_2573-R	CTAGTCTAGA tcaatgcttttccccctcta
BCO26_1484-F	CGCGGATCC atggaacaaggtaaagtaaa
BCO26_1484-R	CCGGAATTC ttataatttcgaaacgttcg
pET28a-CspL-F	CATGCCATGGca atggaacatggtacagtaaa
pET28a-CspL-R	CCGCTCGAG ttagtcttctttttgaacat
CspA-F	CGCGGATCC atgtccggtaaaatgactgg
CspA-R	CCGGAATTC ttacaggctggttacgttac

pYES2-CspL-F	CGCGGATCC atggaacatggtacagtaaa
pYES2-CspL-R	CCGGAATTC ttagtcttctttttgaacat
pME6032-CspL-F	CCGGAATTC atggaacatggtacagtaaa
pME6032-CspL-R	CATGCCATGG ttagtcttctttttgaacat
77-cspL-F	CGGTTGGTAGGATCCACATATGGAGCACGGCA CCGTGAAGT
77-cspL-R	TATGACATGATTACGAATTCAGTCCTCCTTCTG CACGTTGGCG

Supplementary Table 10. Sequences of oligonucleotide used in this study

RNA	Sequence (5’ > 3’)
RNA 1	CGGGAGAGGCGGUUUGCGUAUUGU
RNA 2	GGAGAGGCGGUUUGCGUAUUGU
RNA 3	GGGAGAGGCGGUUUGCGUAU
RNA 4	GGAGAGGCGGUUUGCGU
RNA 5	GCAUUAAUGAAU
RNA 6	GCAUUAAUGAA
RNA 7	CAUUAAUGAA
RNA 8	AUUAAUGAAU
RNA 9	AUUAAUGA
RNA 10	UAAUGA
RNA 11	UAAUG
RNA 12	AAUG
RNA 13	GAAUG
RNA 14	TAAUG
RNA 15	AAUGC
ssDNA 1	AGGTACCCGGGGATCCTCTAGAGTCGTC
ssDNA 2	TGTACCCGGGGATCCTCTAGAGTCGC
ssDNA 3	GTACCCGGGGATCCTCTAGAGTCG
ssDNA 4	GGGAGACCGGAATTCGAGCTCG
ssDNA 5	GAGACCGGAATTCGAGCT
ssDNA 6	GACCGGAATTCGAG
ssDNA 7	CCGGAATTCG
ssDNA 8	GGAATT
ssDNA 9	GAAT
ssDNA 10	GAATC
ssDNA 11	CGAAT
ssDNA 12	TGAAT

Supplementary Table 11. Synthetic gene sequences used in this study

Name	Sequence (5′ > 3′)
cspL-M11	ccatggca gaa cat ggt aca gta aaa tgg ttt aac agt gaa aaa gca gca gca gca gca gca gca gaa ggc gga gac gac gca gca gca gca ttc tcg gcc atc cag ggt gaa ggc tat aaa acg ctt gaa gaa ggc cag aaa gta tca ttt gat gtg gaa gaa gga tca cgc ggc ccg cag gcg gca aat gtt caa aaa gaa gac ctcgag
cspL-M7	ccatggca gaa cat ggt aca gta aaa tgg ttt aac agt gaa aaa gca gca gca gca gca gca gca gaa ggc gga gac gac gtg ttt gtc cat ttc tcg gcc atc cag ggt gaa ggc tat aaa acg ctt gaa gaa ggc cag aaa gta tca ttt gat gtg gaa gaa gga tca cgc ggc ccg cag gcg gca aat gtt caa aaa gaa gac ctcgag
